# Dynamics in zebrafish development define transcriptomic specificity after angiogenesis inhibitor exposure

**DOI:** 10.1007/s00204-024-03944-7

**Published:** 2025-01-09

**Authors:** Julia Nöth, Paul Michaelis, Lennart Schüler, Stefan Scholz, Janet Krüger, Volker Haake, Wibke Busch

**Affiliations:** 1 Department of Ecotoxicology, Helmholtz Centre for Environmental Research—UFZ, Permoserstraβe 15, 04318 Leipzig, Germany; 2 Department of Monitoring and Exploration Technologies, Helmholtz Centre for Environmental Research—UFZ, Permoserstraβe 15, 04318 Leipzig, Germany; 3 BASF Metabolome Solutions GmbH, Tegeler Weg 33, 10589 Berlin, Germany

**Keywords:** Dynamic transcriptome, Zebrafish, Vascular disruption

## Abstract

Testing for developmental toxicity is an integral part of chemical regulations. The applied tests are laborious and costly and require a large number of vertebrate test animals. To reduce animal numbers and associated costs, the zebrafish embryo was proposed as an alternative model. In this study, we investigated the potential of transcriptome analysis in the zebrafish embryo model to support the identification of potential biomarkers for key events in developmental toxicity, using the inhibition of angiogenesis as a proof of principle. Therefore, the effects on the zebrafish transcriptome after exposure to the tyrosine kinase inhibitors, sorafenib (1.3 µM and 2.4 µM) and SU4312 (1 µM, 2 µM, and 5 µM), and the putative vascular disruptor compound rotenone (25 nM and 50 nM) were analyzed. An early (2 hpf—hours post fertilization) and a late (24 hpf) exposure start with a time resolved transcriptome analysis was performed to compare the specificity and sensitivity of the responses with respect to anti-angiogenesis. We also showed that toxicodynamic responses were related to the course of the internal concentrations. To identify differentially expressed genes (DEGs) the time series data were compared by applying generalized additive models (GAMs). We observed mainly unspecific developmental toxicity in the early exposure scenario, while a specific repression of vascular related genes was only partially observed. In contrast, differential expression of vascular-related genes could be identified clearly in the late exposure scenario. Rotenone did not show angiogenesis-specific response on a transcriptomic level, indicating that the observed mild phenotype of angiogenesis inhibition may represent a secondary effect.

**Supplementary Information:**

The online version contains supplementary material available at 10.1007/s00204-024-03944-7.

## Introduction

Exposure to drugs, synthetic and environmental chemicals during pregnancy can have severe consequences for the prenatal development of the embryo in humans and other mammals. Chemicals that are transferred from the mother to the child via the placenta may disrupt developmental processes and lead to the death of the embryo or rigorous birth defects (teratogenesis) in the surviving offspring. The severity of adverse effects is determined by factors such as the critical window of exposure, concentration at the target site, exposure duration, the genetic susceptibility of the embryo, and the mechanism by which the chemical interferes with development (Carney et al. 2004). The most prominent examples are the adverse effects caused by prenatal exposure to alcohol, or the drugs thalidomide and valproic acid. Most severe adverse effects are caused within the first trimester of pregnancy (Mahnke et al. 2019; Parnell et al. 2014). Even though many of the most critical organs and systems develop during early pregnancy, significant consequences of chemical exposure are also described for the last trimester of pregnancy (e.g., for exposures with nicotine or caffeine) (Kirkinen et al. 1983; Vik et al. 2003).

Until now, the assessment of teratogenicity is performed on pregnant model organisms with mainly two different species, namely rats and rabbits. This is time and cost intensive and subject to ethical concerns due to animal experiments. To overcome those limitations, the zebrafish embryo was proposed as an alternative model for providing evidence for teratogenic effects of chemicals. Its rapid development and 70% homology (Howe et al. 2013) to the human genome indicate its suitability for hazard and risk assessment studies. Standardized testing protocols for zebrafish embryos, however, are so far only established for acute fish embryo toxicity (OECD test guideline 236). The protocols for exposure and effect assessment in the zebrafish exhibit a wide variety of exposure scenarios ranging from 0 h to several days post-fertilization (dpf) to acute short exposures of a few hours in later developmental stages depending on the objectives of the study. The experiments are typically performed up to 5 dpf, related to European animal welfare regulations, which consider early stages as non-protective life stage and alternatives to animal testing (Strähle et al. 2012).

For the present study, anti-angiogenesis was selected as a case study, because the formation of the cardiovascular system is a complex biological process and disturbance of vasculogenesis and angiogenesis are linked to developmental toxicity (Knudsen and Kleinstreuer 2011). The blood vessel system is one of the first structures differentiating during embryogenesis and is important for the transport of oxygen and nutrients through the organism. Several studies have shown that the zebrafish embryo is suitable to investigate blood vessel development and quantify anti-angiogenic effects caused by chemicals (Nöth et al. 2024; McCollum et al. 2017; Tal et al. 2017) and the formation of the vascular system in the developing zebrafish is already described in detail (Isogai et al. 2001). Vascularization of the embryo is initiated within the first 24 hpf (hours post fertilization). A simple single circulating loop consisting of aorta and vein is already build by vasculogenesis. Subsequently, the formation of new vessels out of pre-existing ones are developing via sprouting angiogenesis. Within 48 hpf the blood circulation is established. The development of the vascular system is initially not required for oxygen supply until a stage of around 7–14 dpf (Cha and Weinstein 2007; Jacob et al. 2002; Rombough 2002). This may be considered as an advantage over vertebrate model organisms as oxygen supply as a confounding factor for toxicity can be excluded (Lahnsteiner 2008).

Various drugs and chemicals are known to interfere with angiogenesis and provoke teratogenic effects. Thalidomide, a drug still applied to treat different type of diseases, is a prominent example of an angiogenesis inhibitor causing teratogenic effects when applied during pregnancy. It inhibits the growth of naive, newly forming blood vessels and caused severe birth defects in around 10,000 children due to its initially unknown teratogenic potential (Vargesson 2015; D'Amato et al. 1994). Other chemicals that interfere with estrogen hormone levels (e.g. bisphenol A), antagonists for native endothelin receptors, as well as retinoids, metals and compounds of cigarette smoke (Knudsen and Kleinstreuer 2011) have been shown to disrupt the formation of the vascular system via different mechanisms.

Anti-angiogenic drugs are studied with increasing interest, because they also have a potential in clinical treatment of various cancers and diseases (Carmeliet and Jain 2000; Adams and Leggas 2007). While anti-angiogenesis represents the therapeutic effect for these drugs, the same compounds can be applied to investigate and develop assays for the detection of angiogenesis inhibition and teratogenicity. A known mechanism for anti-angiogenesis is the inhibition of tyrosine kinases, which are involved in a wide spectrum of developmental processes from very early events of fertilization and gastrulation to later events such as organogenesis (Challa et al. 2013). They are involved in the signaling pathways of cell proliferation, differentiation, migration, metabolism, and apoptosis. Especially, the development of the cardiovascular system is regulated by two tyrosine kinases: vascular endothelial growth factor receptor (VEGFR) and platelet-derived growth factor receptor (PDGFRβ). In zebrafish, two orthologue genes are known to encode VEGFR2, *kdr* and *kdrl*. VEGFR binds the ligand vegfa and is described to have similar functions as the human receptors (Toselli et al. 2019; Vogrin et al. 2019).

In our previous research (Nöth et al. 2024), we established concentration-dependent inhibition of angiogenesis for four tyrosine kinase inhibitors (TKIs) and demonstrated the impact on zebrafish embryo development. Based on our morphological observations and high anti-angiogenic specificity the TKIs SU4312 and sorafenib were selected, to study the transcriptional response of anti-angiogenic compounds. Both compounds bind to the ATP-binding pocket of the kinase domain of the VEGFR2 and respective anti-angiogenic effects have been shown using cell-based and zebrafish embryo assays (Tran et al. 2007; Nöth et al. 2024). SU4312 and sorafenib showed a dose-dependent depletion of intersegmental blood vessels in 96 hpf old zebrafish embryos, which suggests them as promising model compounds for the identification of molecular markers of angiogenic disruption (Nöth et al. 2024). Both compounds led to an angiogenesis inhibition phenotype, i.e., malformation or loss of functional ISVs. When exposure was initiated at 2 hpf additional severe malformations were observed. A later exposure start at 24 hpf caused anti-angiogenic effects at the same concentrations, but caused less morphological side effects and lethality. The morphological observations indicated that assessment of transcriptional responses to identify the underlying key events may be confounded by gross morphological development effects. We hypothesized that using a late exposure start could result in higher specificity of the anti-angiogenetic response and that the transcriptional response could change over exposure duration time.

Therefore, we measured transcriptional responses in different exposure scenarios, in a time-resolved manner and developed an analysis pipeline for the statistical comparison of time-resolved gene expression data at the whole transcriptome scale. In addition to the TKIs described above, sorafenib and SU4312, we included rotenone, an insecticide often described as potential vascular disruptor (Pung et al. 2013; McCollum et al. 2017). Rotenone is an inhibitor of mitochondrial complex 1 and can cause a reduced ratio of active mitochondria, increased ROS production, and decreased ATP production, and it is also used in research to mimic Parkinson disease. By comparing the toxicogenomic responses of the different chemicals across the different time points, we aimed to identify molecular markers of angiogenesis disruption in zebrafish. The aim was to identify markers that allow to identify and confirm key events contributing to the existing adverse outcome pathway for angiogenesis disruption via VEGFR inhibition (AOP43) as predictive markers for developmental toxicity (Knudsen and Kleinstreuer 2011; McCollum et al. 2017; Tal et al. 2017).

## Materials and methods

### Chemicals

The two tyrosine kinase inhibitors SU4312 (CAS No. 5812-07-7), sorafenib (CAS No. 284461-73-0) and the insecticide rotenone (CAS No. 83-79-4) were purchased from Sigma Aldrich. Stock solutions were prepared in 100% dimethyl-sulfoxide (DMSO). Therefore, a 10,000-fold stock solution was prepared, stored at room temperature until usage and diluted in ISO water as specified in ISO 7346-3 (1996) [80 mM CaCl_2_·2H_2_O, 20 mM MgSO_4_·7H_2_O, 31 mM NaHCO_3_, 3.1 mM KCl] on the day of the experiment. Final pH was adjusted to 7.4 (pH 7110, InoLab), a minimum oxygen saturation of 80% was verified using WTW Oxi 340 Oximeter and concentration of DMSO was 0.01% (v/v) in all test concentrations and control.

### Zebrafish husbandry, embryo collection

A wild type adult zebrafish strain (*D. rerio*, strain OBI/WIK, generation F3) originating from an in-house cross of zebrafish strains was used for all experiments. Fish were fed twice daily *ad libitum* with commercial dry food (SDS-400, Special Diets Services, England) and *Artemia sp*. The strain was cultured under a photoperiod of 14 h light/10 h dark in 20 L aquaria with 1–2 fish per liter density (tap water, 26.5 ± 1.0 °C). Physicochemical parameters of the aquaria water were frequently measured (pH 7–8; water hardness 2–3 mmol/L, conductivity 540–560 S/cm, nitrate < 2.5 mg/L, nitrite < 0.025 mg/L, ammonia < 0.6 mg/L, oxygen saturation 87–91%). Facilities for breeding and the production of embryos were licensed by the local government agency (Landesdirektion Leipzig, Aktenzeichen 75–9185.64). Spawning trays were inserted 4–6 h before the end of the light cycle. Spawning was initiated by the onset of light and eggs were collected within 1 h after spawning. Fertilized and normally developed embryos were selected according to (Kimmel et al. 1995) with a dissection microscope (Olympus SZx7-ILLT). For the later exposure starting point (24 hpf) 50 embryos were stored in 100 mL of ISO-water for 24 h at 26 °C, shaken at 75 rpm using a horizontal agitator (Edmund Bühler GmbH, SM–30 control) until exposure start.

### Exposure conditions in zebrafish embryos

The exposure concentrations were phenotypically anchored to the observed effect concentration at two different exposure scenarios (C_low_, C_medium_, and C_high_, representing EC10, EC50, and EC90, respectively) at 96 hpf (Nöth et al. 2024), because we observed differences in phenotypical effects regarding the exposure start of tyrosine kinase inhibitors. Therefore, an early exposure scenario reflects severe developmental toxic effects, while a late exposure does not result in a lot of malformations/lethality and seems to be suitable to identify genes related to blood vessel formation. Based on this observation for both exposure scenarios concentrations were selected: a high, medium and low dose, respectively, as described in Table 1.

**Table 1:** Exposure concentrations of SU4312, sorafenib and rotenone

	C_low_	C_medium_	C_high_
SU4312	1 µM	2 µM	5 µM
Sorafenib	1.3 µM		2.4 µM
Rotenone	25 nM		50 nM

**Fig. 1:**
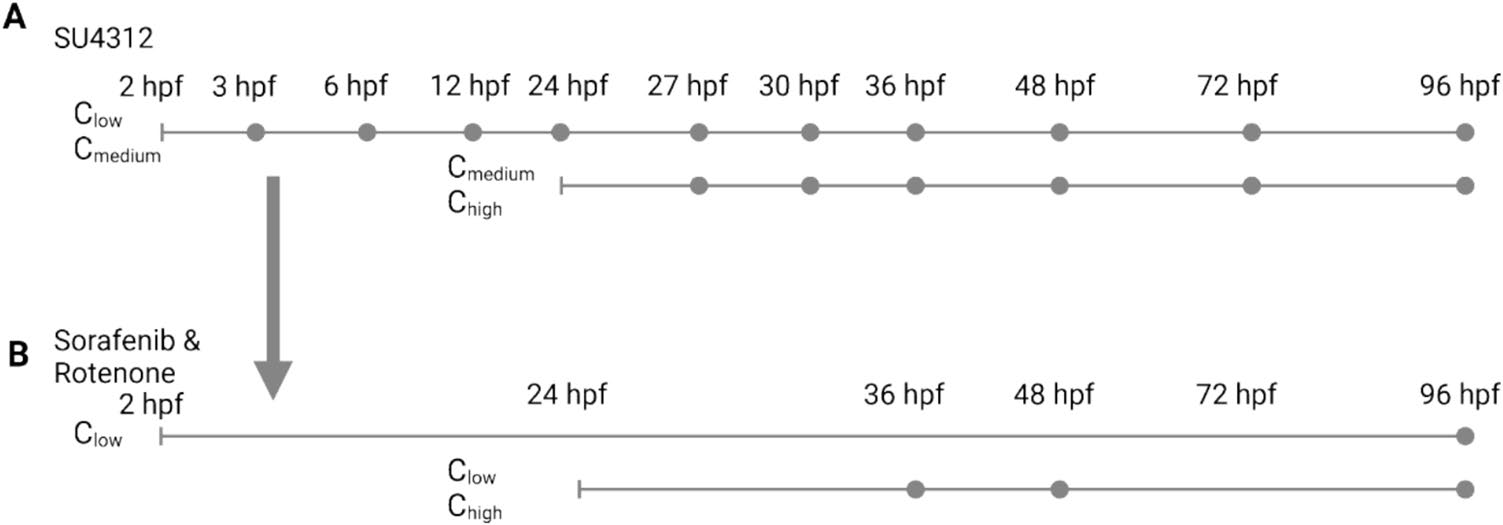
The exposure scenario for (**A**) SU4312 exposure was conducted as a proof of principle experiment with a high-resolution time series. (**B**) Based on the first exposure scenario indicated by the arrow, a reduced transcriptomic scenario was chosen for sorafenib and rotenone. Early exposure (2 hpf) and late exposure (24 hpf) sampling points refer to the same exposure durations. Concentrations are described in Table 1

The eggs were exposed from 2 h (early) after fertilization (16 to 128 cell stages) or from 24 hpf (late). To obtain the time- and concentration-dependent transcriptome and internal concentrations of exposed zebrafish embryos, two replicates of control and one replicate of treatment were used. The experimental design was giving preference for multiple concentrations versus many replicates with few time points to allow a time-resolved modeling. In total 10 embryos were exposed to 4 mL of exposure or control solution and incubated for the desired exposure time in 7.5 mL GC vials (VWR International, Darmstadt, Germany) closed with aluminum lid and aluminum coated septum (Supelco Analytical, Munich, Germany). Two vials were pooled as one replicate (in total 20 embryos). Vials were incubated at 26 °C with a 12:12 h light:dark photoperiod and shaken at 75 rpm using a horizontal agitator (SM–30 control, Edmund Bühler GmbH, Bodelshausen, Germany) (Fig. 1).

### Internal concentrations measurement of SU4312

A calibration was performed concentration range of 5 to 160 mg/ml SU4312. The calibration was used to determine the substance concentration in the study samples.

Internal concentration was measured in samples of 20 pooled zebrafish embryos by extraction with acetonitrile using a ball mill (Bead Ruptor, Biolabproducts GmbH, Bebensee, Germany). After centrifugation (11,290*g* (12,000 rpm), 10 min at 15 °C), 100 µl of the supernatant were transferred to a new vial. A 5 µl aliquot of the supernatant was subjected to LC–MS/MS analysis using a biphenyl column (Raptor Biphenyl 50 × 2.1 mm l.D. 2.7 µm, Restek) followed by MS/MS detection (AB Sciex QTrap 6500 +) in positive ionization mode. Gradient elution was performed with 5% acetonitrile in water with 0.5% (w%) formic acid (A) and acetonitrile with 0.5% (w%) formic acid (B) (0 min 80% A, 1:30 min 35% A, 2:10 min 25% A, 2:20 min 0% A, 700 µl/min, 40 °C). As reference, samples from the incubation solutions were diluted in water (1:1 w/w).

For visualization, loess splines were used as implemented the R stats package, the data can be found in the supplement (Supplementary file 17).

### RNA extraction

Two vials (20 zebrafish embryos in total) were harvested per replicate. Embryos were transferred into FastPrep tubes (MP Biomedicals), exposure media was removed, 150 µL ice-cold Trizol was added and stored at − 80 °C until further usage. Homogenization was performed by a FastPrep 5G (MP Biomedicals) for 30 s at 6.0 m/s two times. In between both steps the samples were incubated on ice for 1 min. The samples were centrifuged for 5 min at 4 °C at 120000 g. Supernatant was transferred into a fresh 1.5 mL Eppendorf tube. RNA isolation was performed using a pipetting robot (Microlab Star, Hamilton Life Science Robotics) following the manual provided for Total RNA Extraction Kit MagMAX 96 for microarrays and conducted in an 1.5 mL Eppendorf tube. RNA was eluted with a volume of 50 µL. The quality of isolated RNA was assessed using a Nanodrop and Bioanalyzer (Agilent 2100 Technologies) with the Agilent RNA 6000 Nano Kit. RNA samples were used for further processing if the RNA integrity number (RIN) values derived from ribosomal RNA absorption adopted values > 7 and calculated concentrations exceeded 25 ng/μL.

### Gene expression preparation

All RNA samples of SU4312 exposed whole zebrafish embryos were diluted to a concentration level of 10 ng/μL (50 μL in total) by adding RNAse free water. 2.3 μL were used as the starting amount of RNA for the spike mix preparation. Transcript abundance was measured with microarray analysis using Oaklabs ArrayXS Zebrafish microarray slides (XS-200,104, Oaklabs; National Center for Biotechnology Information Gene Expression Omnibus platform accession: GPL19785). The microarray experiments were performed using the Agilent Low Input Quick Amp WT Labeling Kit according to the Agilent One-Color Microarray-Based Exon Analysis Protocol. This protocol included the introduction of spike-in RNA, RNA transcription, and amplification into complementary DNA (cDNA), and cDNA transcription and amplification into cRNA with simultaneous incorporation of Cy3 (fluorescently labeled cytidine nucleotide). The cRNA was fragmented and hybridized to Oaklabs ArrayXS Zebrafish microarray slides using the Agilent hybridization kit and protocol as well as Agilent hybridization oven and chambers. Subsequently, microarray slides were washed and scanned with the Agilent high-resolution microarray scanner according to the Agilent protocol. The intensity values were extracted from captured images using Agilent Feature Extraction software (version 11.5.1.1).

The laboratory changed from microarrays to a RNA-sequencing approach for transcriptome assessment of sorafenib and rotenone exposure. All RNA samples were diluted to a concentration level of 25 ng/μL (10 μL in total) by adding RNAse free water. Sequencing libraries of sorafenib and rotenone exposed whole zebrafish embryos were generated using NEBnext Ultra II stranded RNA library preparation kit for Illumina (NEB, Beverly, MA, USA), and index codes were added to attribute sequences to each sample. The library preparations were sequenced using the Illumina NovaSeq 6000 platform (Illumina Inc., San Diego, CA, USA) with a paired-end sequencing length of 100 bp (PE100). The sequencing was performed at CRTD, Deep Sequencing Group (Dresden, Germany).

All transcriptome data is available at GEO with accession GSE270294 for the SU4312 dataset and GSE270785 for the sorafenib and rotenone dataset.

### Data processing

Quality control was performed on the samples using four metrics: the Kolmogorov–Smirnov test, the sum of all expression values per array, the interquartile range (IQR), and the Euclidean distance. A sample was flagged as an outlier if it did not fall within the range of the 25% and 75% quantile ± 3 × IQR (1 × IQR for Euclidean distance). One sample (DMSO, exposure start at 0 hpf, 36 hpf) was flagged as an outlier and removed from the dataset. Subsequently, the data were log2 transformed and normalized between arrays using the cyclic loess method and, where applicable, the median was calculated for replicate probes. All of the aforementioned steps were carried out using the toxprofileR2 package (https://git.ufz.de/iTox/toxprofiler2), which mainly utilizes functions from the limma package (version 3.55.10) (Ritchie et al. 2015).

For RNA sequencing, FASTQ files were processed using Galaxy (The Galaxy platform for accessible, reproducible and collaborative biomedical analyses: 2022 update 2022). HISAT2 (version 2.1.0) (Kim et al. 2019) was used to align reads to the zebrafish genome GRCz11. Quality of alignments were filtered for mapping quality (MAPQ > 30) and featureCounts (version 2.0.3) (Liao et al. 2014) was used to count reads. Samtools (version 2.0.4) (Danecek et al. 2021) was used for the filtering as well as to read out information of relevant steps, which was then compiled using MultiQC (version 1.11) (Ewels et al. 2016). All further processing was done in R. Using principal component analysis two samples were identified as outliers (DMSO, exposure start at 0 hpf, 96h and DMSO, exposure start at 24 hpf, 48 hpf) and were omitted from the dataset (see Supplementary file 1 for further details). In the case of the 0 to 96 hpf exposures, DESeq2 (version 1.40.1) (Love et al. 2014) was used to calculate logFCs and p-values. The genes were determined differentially expressed genes (DEGs) when they exhibited a *p*-value cutoff of 0.05, after *p*-value adjustment using the Benjamini & Hochberg method. For the 24 hpf exposure samples counts were transformed to log2-counts per million using limmas voom method (Law et al. 2014). With this method, microarray and RNA-sequencing data could be compared and modeled over time using the same models.

### Modeling of expression over time

**Fig. 2:**
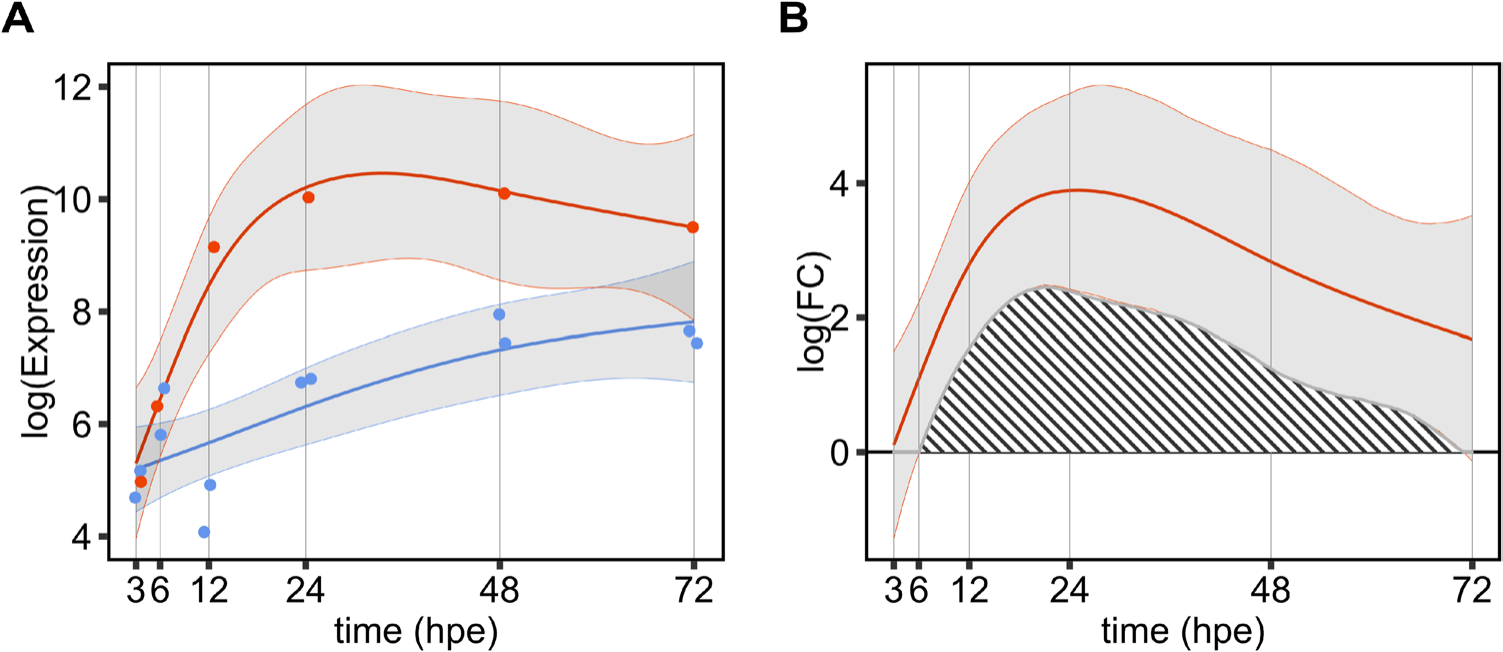
Generalized additive models fitted to data and simulated difference to control over time for cyp1c1. **A** Log transformed normalized gene expression data were modeled individually over time using GAMs, blue represents control data, red the data for concentration level C_median_, grey are the 95% confidence bands. **B** The shaded area represents the differences between control and treatment over time and is calculated as area between the respective 95% confidence intervals and used as a measure of the effect size (AUCI_95%_)

To model gene expression over time generalized additive models (GAMs, as implemented in the R package mgcv) (Hastie und Tibshirani 1986) were used as outlined in (Eq. 1):

1 $$E \sim concentration{\text{\_}}level + s\left( {time} \right),$$

where *E* is the log(Expression) of a gene, *concentration_level* is a categorical variable describing the treatment and *s(time)* is the penalized smooth term for the trend over time, with a replicate smooth defined for each concentration level. To account for differing numbers if time points, dimension *k* of the basis used to represent the smooth terms, which relates to the degrees of freedom of the smooth, was chosen to be the number of time points of the respective experiment. This way, models were allowed to accurately represent the data, even in the sections where sampling points were in close proximity (Fig. 2A). To assess the deviation of the treatment curves from their respective control curves, for each gene, the models were simulated 10,000 times with model parameters sampled from a multivariate normal distribution according to the initial model. The control curves were subtracted from their respective treatment curves, which allowed us to calculate the average difference and confidence interval over time. To create a meaningful effect measure of differential expression, we calculated the signed area under the 95% confidence interval of the difference to the control (AUCI_95%_ Fig. 2B). To compare different time frames, the AUCI_95%_ was normalized by the exposure duration in hours, resulting in the AUCI_95%_/h.

### Differential expression analysis

To obtain meaningful sets of DEGs, we decided to use a cutoff on the |AUCI_95%_/h| to determine differential expression. To find the optimal cutoff, we generated curves for 300 randomly selected genes (150 from each exposure start) and visually assessed whether they were differentially expressed or not differentially expressed. Matching the classification of the curves to their |AUCI_95%_/h| gave us a discrete cost function of wrongly classified curves given a |AUCI_95%_/h| cutoff. As the parameter space of this function is only one-dimensional, we used the brute force method implemented in SciPy (version 1.11.1) to minimize the function and find the optimal cutoff for the |AUCI_95%_/h|. This resulted in optimal cutoffs of 0.05 for the early exposure, 0.047 for the late exposure and 0.046 for all curves combined. Given that the values between early and late exposure do not differ greatly, we chose to use the combined cutoff of 0.046 for all subsequent analyses. We also want to point out that the calculated optimal cutoff lies in a very distinct minimum of the cost function, giving us confidence in this method (Supplementary file 4).

### Clustering

For clustering, hierarchical clustering as implemented in the hclust function from the R stats package (version 4.3.0) was applied, using Euclidean distance to compute the distance matrix.

### Overrepresentation analysis

The sets of DEGs were determined for the several exposure conditions and in various time frames. To gain insight into the biological functions that these DEGs represent we used overrepresentation analysis (ORA) (Boyle et al. 2004), implemented in the R package clusterProfileR (Wu et al. 2021). This method uses the hypergeometric distribution to calculate probabilities that the occurrence of biological functions among a set of DEGs could be due to chance. ORA was conducted using gene sets of five databases: ZFIN (Bradford et al. 2022), Interpro (Mitchell et al. 2019), Reactome (Fabregat et al. 2018), Gene Ontology (Ashburner et al. 2000), and MSigDB (Liberzon et al. 2011). In the case of MSigDB, which does not contain zebrafish gene sets, several collections of human gene sets were downloaded (see Supplementary file 1) and biomaRt (version 2.56.1) (Durinck et al. 2005) was used to find zebrafish orthologs. Gene sets were excluded if fewer than 50% of the human genes had zebrafish orthologs. *P*-values were adjusted using the Benjamini and Hochberg method and terms with adjusted p-values < 0.05 were considered significant.

### Availability of source code and requirements

All statistical analyses were conducted in R, apart from the determination of the AUCI_95%_/h cutoff, which was conducted in Python (version 3.7.6). The complete analysis is available as an R Markdown document at https://codebase.helmholtz.cloud/ufz/tb3-cite/etox/itox/2022_zfaop_omics. This document includes further explanations and information on used packages. A compiled HTML-version of the document can be found in the supplement (Supplementary file 1).

## Results

### SU4312 exposure causes dynamic responses in the zebrafish embryo transcriptome

**Fig. 3:**
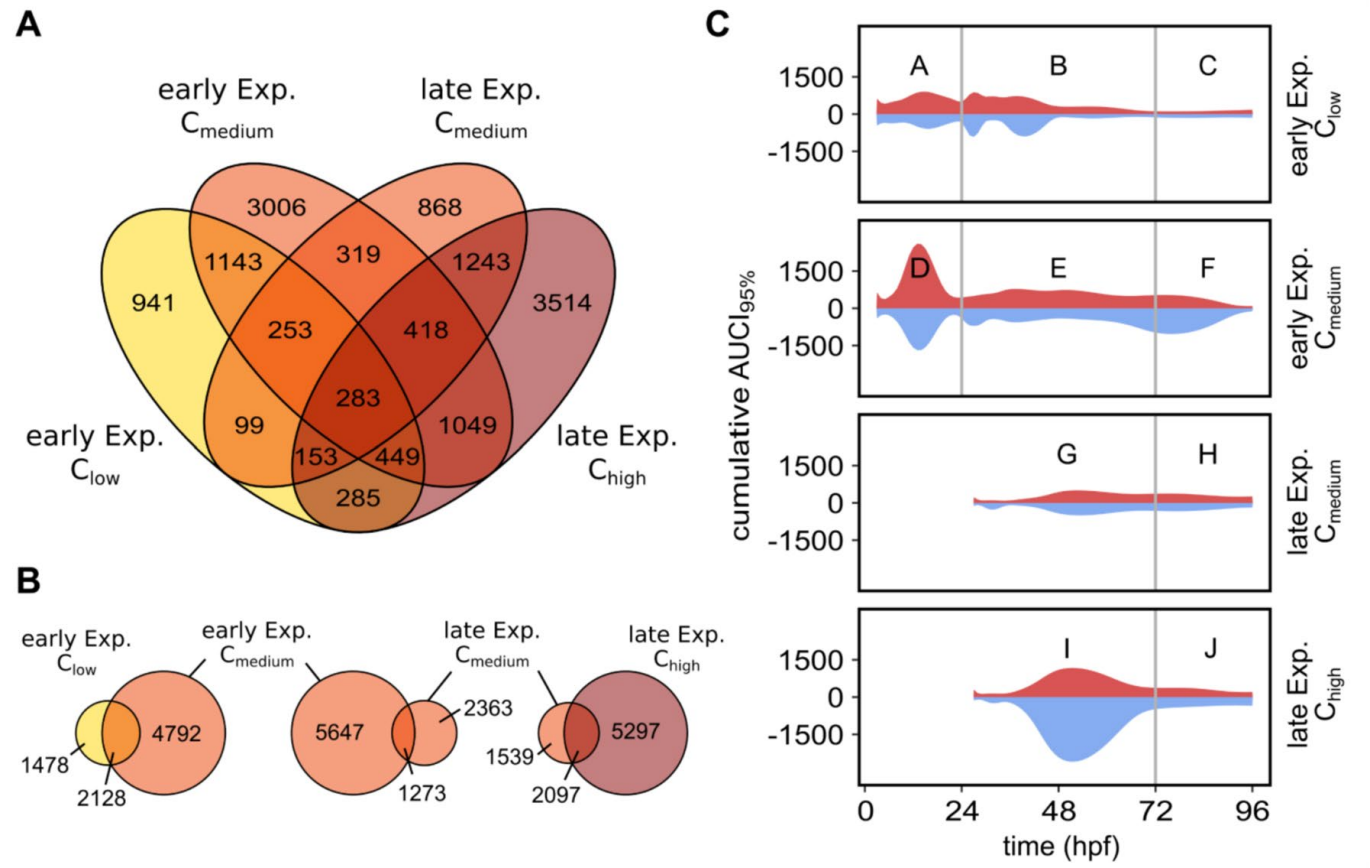
Numbers of DEG after SU4312 exposure. **A** Venn Diagram showing overlapping number of DEG for all tested concentrations (C_low_ = 1 µM, C_medium_ = 2 µM and C_high_ = 5 µM) in early (2 hpf) and late exposure (24 hpf) scenario showing a total overlap of 283 genes across all test conditions. **B** Three Venn diagrams comparing only conditions in early exposure, same concentration or only late exposure scenario. (**C**) The amount of DEGs is changing dynamically. AUCI_95__%_ profiles for all genes were added to create these patterns. Areas above zero in red relate to up-regulated genes, areas below zero in blue to down-regulated genes. Time frames are indicated as 0–24 hpf (**A** and **D**), 24–72 hpf (**B, E, G, I**), and 72–96 hpf (**C, F, H, I**)

To determine specific molecular markers for anti-angiogenesis in zebrafish and to reveal a suitable exposure window, we exposed zebrafish embryos from an early (2 hpf) and late (24 hpf) exposure start time point with two concentrations of SU4312, respectively (early: C_low_ = 1 µM, C_medium_ = 2 µM; late: C_medium_ = 2 µM and C_high_ = 5 µM) and obtained samples at 6 and 10 different time points, respectively. Based on the above-described data analysis workflow which considers all time points within the statistical analysis, we found 3603 differentially expressed genes (DEGs) with C_low_ and 6920 DEGs with C_medium_ for the early exposure scenario (Fig. 3A). Following the later exposure with C_medium_ and C_high_ we identified 3636 and 7394 DEGs, respectively (Supplementary file 13). These numbers indicate a concentration-dependent increase in global gene expression in the organism in both scenarios, early and late. DEGs obtained in exposure scenarios with different concentrations but with the same exposure start point exhibited proportionally larger overlaps (25% for early exposure with C_low_ vs. C_medium_, and 23% for late exposure with C_medium_ vs. C_high_) than the overlaps of DEGs measured after the different exposure starts (ranging from 9 to 14%). 240 genes (1.6% of all DEGs) were differentially expressed in all conditions (Fig. 3A). Another relevant comparison is early C_medium_ (6920 DEGs) versus late C_medium_ (3636 DEGs), as these scenarios share the same exposure concentration. The overlap is relatively small (11%) (Fig. 3B), but the number of DEGs is almost twice as large in the early exposure scenario compared to the late one, indicating a strong effect of exposure within the first 24 h of development (Fig. 3B).

**Fig. 4:**
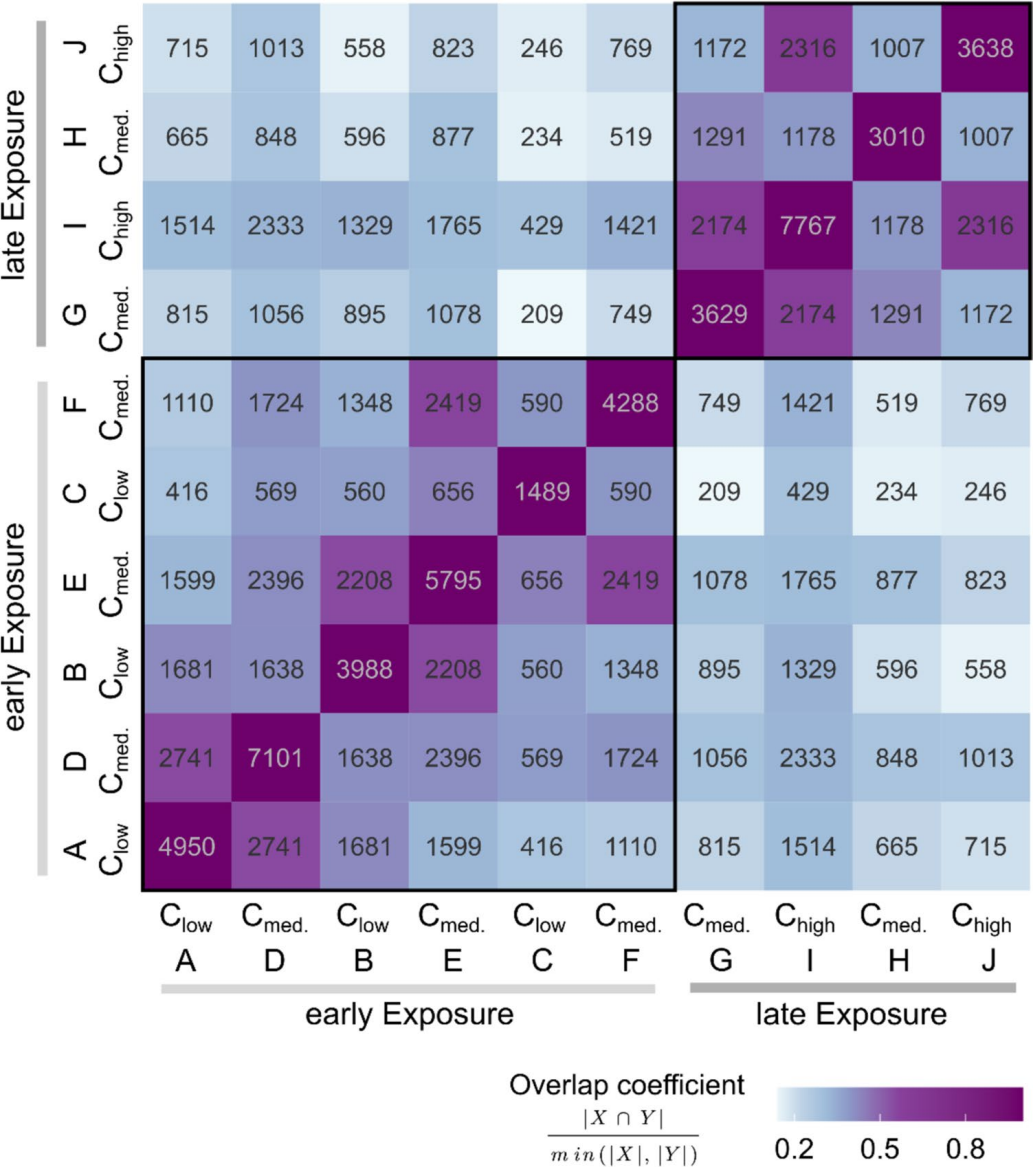
Overlaps of SU4312 DEG sets. Numbers within the cells are jointly affected genes or the DEG set sizes (diagonal). The larger the overlap coefficient (size indicated with the blue to purple colours) the larger is the proportion of genes that are affected in both of the compared conditions

Next to the pure amount of DEGs in each condition, we aimed to illustrate the dynamics of the respective transcriptomic changes. Therefore, we combined the AUCI_95%_-profiles of all genes per condition (exposure start point and concentration) to gain a picture of the global time-dependent dynamics and quantity of up- and down-regulation in the zebrafish transcriptome (Fig. 3C). For a more detailed analysis of genes and pathways we defined three time frames. Time frames A and D, spanning the first 24 hpf are only present in the early exposure dataset. While time frames from 24 to 72 hpf (B, E, G and I) and 72 to 96 hpf (C, F, H and J) are congruent in the early and late exposure scenario (Supplementary file 9). In the sections A and D (Fig. 3C) a distinct peak of transcriptional activity can be observed, especially with C_medium_, the higher concentration, which then levels off to a lower level of regulation for the rest of the exposure duration (B, C, E, F). Similarly, there is a peak of transcriptional activity at 48 hpf following exposure C_high_ in the late exposure scenario (Fig. 3C (I)), which is not present at this time point in the early exposure scenario. At 96 hpf the smallest number of genes are affected regardless of the exposure condition (Fig. 3C (C, F, H, J), and Fig. 4). The number of up- and down-regulated genes was similar for each time point, and independent of time points or concentrations.

Figure 4 shows the numbers of DEGs in more detail for the different time windows A–J, colors indicate the overlap. The samples were ordered based on exposure start and concentration to pronounce similarities. Particularly two larger clusters that separate the early and late exposure scenarios from each other, were identified. Hence, the major contributor to the determination of variance in expressed genes can be explained by early and late exposure start. Within the same time frame, e.g. early or late, overlaps are larger for same exposure durations than for same concentrations, exceptions are here the respective longest exposure scenarios with the respective highest concentrations (C and F in the early exposure time frame with a larger overlap coefficient between F and E, and C and E; and H and J in the late exposure time frame with a larger overlap coefficient between I and J, and I and H). The least similarities can be observed comparing early with late exposure scenario and a separation between different concentrations (Fig. 4).

### Exposure start impacts on gene expression patterns after SU4312 exposure in zebrafish embryos

**Fig. 5:**
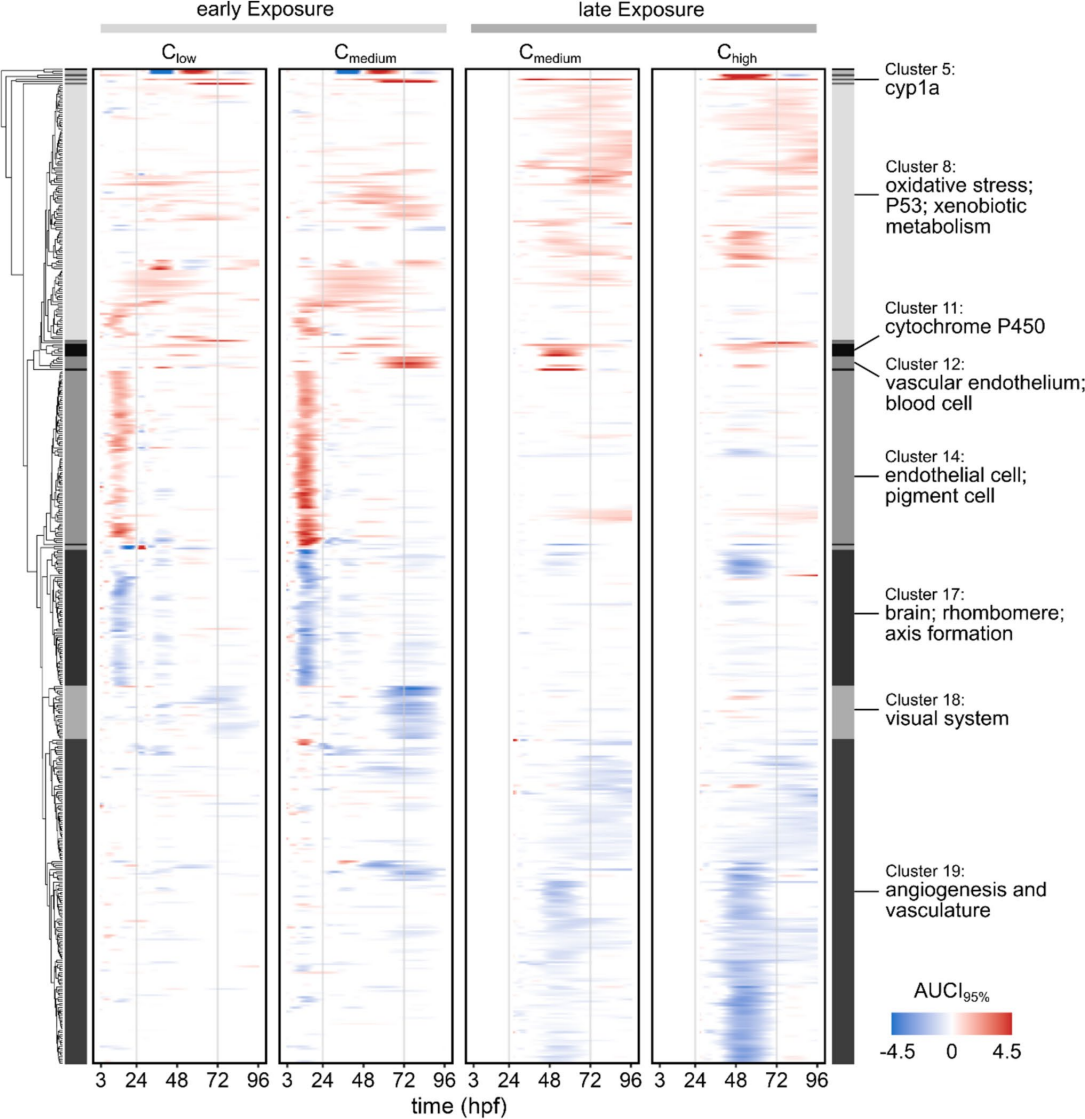
Dynamic expression patterns of 484 top DEGs in response to SU4312 exposure. 19 clusters were identified by hierarchical clustering based on GAM modelled gene expression across all exposure scenarios. Functional annotations are shown for the most prominent clusters

Figure 5 shows the expression patterns of the top 250 differentially expressed genes of the early exposure scenario, selected by largest absolute cumulative AUCI_95%_/h across all time frames and concentrations, and the top 250 genes of the late exposure scenario. The clustered heatmap consists of 484 genes, while early and late exposure scenarios share 16 genes. Within the 484 genes, 296 are differentially expressed in both exposure scenarios, while 106 are differentially expressed in the early exposure scenario but not in the late and vice versa for 82 genes (Supplementary file 7). Using hierarchical clustering, we identified 19 clusters based on the expression patterns across all exposure scenarios. We then performed an ORA with all clusters containing at least five DEGs (Supplementary file 15). Key terms per cluster are highlighted in Fig. 5. Cluster 8 is one of the larger clusters and contains 124 mainly up-regulated genes. Biological process enrichment connected those to oxidative stress response and xenobiotic metabolism. However, the pattern of regulation is different between the early and late exposure scenarios. Cluster 11 is rather small and consists of six genes coding for cytochrome P450 enzymes, which are up-regulated mostly in the late exposure scenario. Additionally, cyp1a, which builds its own cluster, is strongly up-regulated after 24 h in all conditions.

In cluster 17 (rhombomere, brain and axis formation) most genes are down-regulated within the first 24 hpf in the early exposure condition (time frame A and D). Additionally, 26 genes related to the visual system (Cluster 18) and eye development are also mainly down-regulated in the early exposure scenario but not at the beginning but rather at the end of the exposure duration. At the same time points some of those genes are also affected in the late exposure scenario.

Cluster 14 (endothelial cells and pigment cells) and cluster 12 (vascular endothelium and blood cells) together consist of 90 genes, which are up-regulated predominantly within the first 24 hpf in the early exposure scenario. The biggest cluster (19) consists of 158 genes related to angiogenesis and vascular formation. Notably, most of those genes are down-regulated in the late exposure scenario between 30 and 72 hpf. This is an important angiogenesis related time window during development.

**Fig. 6:**
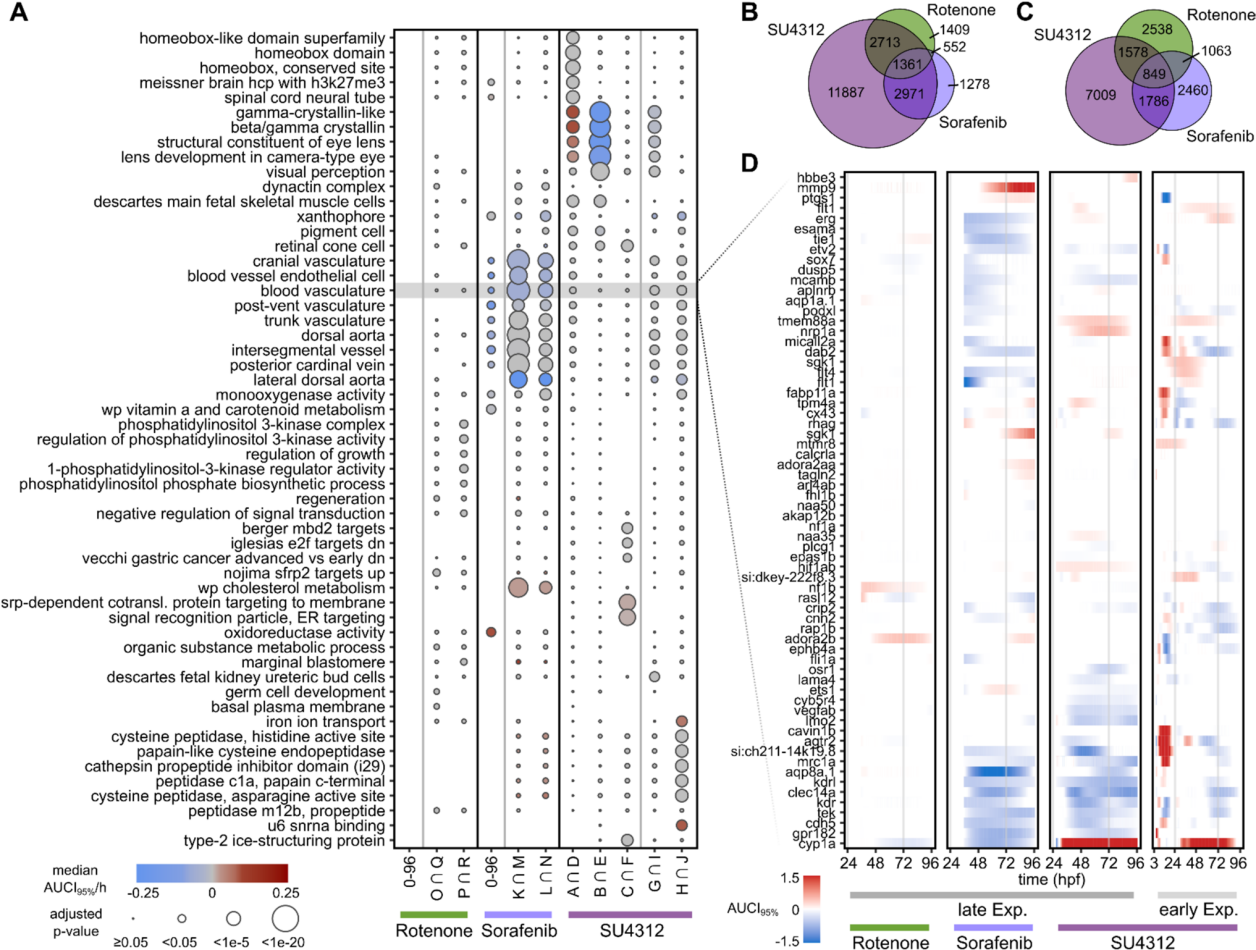
**A** Significant functional terms after rotenone, sorafenib and SU4312 exposure. Overrepresentation analysis of five databases (ZFIN, Interpro, Reactome, Gene Ontology, MSigDB) representing biological processes. The x-axis represents test substance, exposure condition (early, late) divided in exposure duration (A and D = 0–24 hpf, B, E, G, I, K, M, O, Q = 24–72 hpf, C, F, H, J, L, N, P, R = 72–96 hpf). size of the bubbles indicates the p-adj, and color represents the trend of down- (blue) or up-regulation (red) in a gene set, calculated as the median AUCI_95%_/h. Grey indicates no change in median, meaning up and down-regulation is equal. Letters describing the columns denote the different time frames and exposure concentrations as shown in Fig. 4 and Fig. 7. **B** Venn diagram of DEGs across all compounds and exposure scenarios. **C** Venn diagram of DEGs for SU4321, sorafenib and rotenone in the late exposure scenario. **D** Transcriptional profiles of modelled gene expressions comprising the ZFIN term ‘blood vasculature’ (ZFA:0001079). Gene expression effect size compared to control is shown by the colors, measured over time for exposures of zebrafish embryos with the three chemicals SU4312, sorafenib and rotenone

Next to this heatmap of the most prominent DEGs we conducted an ORA on all DEGs using genes sets of five databases (ZFIN, Interpro, Reactome, Gene Ontology, MSigDB) and compared the results between the different exposure conditions, and, later on, also with those of two additional compounds (Fig. 6A). Tables of resulting significantly over-represented terms (adj. *p*-value < 0.05) are provided in Supplementary file 11 and Supplementary file 10, as well as a table outlining numbers of DEGs and over-represented terms for the respective conditions (Supplementary file 14).

In the early exposure scenario with SU4312, time frame A and D (0–24 hpf) had the most over-represented functional terms out of all SU4312 enrichments that have been carried out. These time frames also showed the largest numbers of DEGs (see also Fig. 3 and Fig. 4). Some highly significant terms are related to early development (Homeobox gene sets were among the most significant terms) and especially to structures related to brain development, such as ‘rhombomere, midbrain, thalamus, forebrain’. Eye and lens related gene sets were over-represented as well, with genes being predominantly up-regulated within this time-frame. An angiogenesis-specific response could not be observed within this early time-frame. Only a few angiogenesis-related terms were barely significant with adjusted p-values just below 0.05, while other related terms were just over the cutoff. Terms related to the development of eye and lens were still among the most over-represented ones in time frame B and E (24–72 hpf) following the early exposure to SU4312. However; at this stage genes were predominantly down-regulated. Unlike in A and D, angiogenesis-specific terms were not significantly over-represented. In time frames C and F (72–96 hpf) of the early SU4312 exposure, terms related to endoplasmic reticulum targeting, RNA processing and, to a lesser extent than in the time frames before, visual perception were over-represented. Again, angiogenesis-related terms were not over-represented in this gene set.

Following the late exposure starting at 24 hpf we looked at two time-frames for DEGs and associated biological functions. In time-frames G and I (24–72 hpf) after SU4312 exposure, terms related to the visual system were significantly enriched as in B and E, the same window analyzed after early exposure. Contrasting to the findings of the early exposure scenario, we identified functional terms related to angiogenesis and vasculogenesis as significantly over-represented in the late exposure scenario with SU4312. In time-frames H and J (72–96 hpf) of the late exposure scenario terms related to general cell maintenance functions, such as ‘histone folding’ and ‘chromosome’ were over-represented. At this stage angiogenesis-related terms were still significant, but also other terms related to metabolism and specific families of proteins could be observed. This includes for example ‘iron ion transport’, the ‘cytochrome P450 family’ or ‘cysteine peptidases’.

### Specific identification of genes and gene sets related to effects on the vascular system after exposure with TKI in ZFE

Based on these findings, we aimed to investigate whether the down-regulation of angiogenesis-related genes and the over-representation of respective terms can also be detected for other vascular disruptor compounds, namely the TKI sorafenib and the insecticide rotenone considering early and late exposure scenarios (Supplementary file 8). Therefore, we exposed zebrafish embryo at the early (2 hpf) and the late (24 hpf) time point and measured changes on the transcriptome after exposure for different durations (96 h for the early exposure, and 12, 24, 48 and 72 h for the late exposure). The exposure concentrations were selected based on effect concentrations observed for lethality and morphology (Supplementary file 2).

Comparing the results for the three different chemicals showed little effects of sorafenib and rotenone obtained in the early exposure scenario, but an overlap of a substantial number of genes for the late exposure scenario, where 849 DEGs were found to be affected with all three compounds (Fig. 6C, Supplementary file 5) and 1361 DEGs were found to be regulated in the exposures to all three compounds in any exposure scenario (Fig. 6B).

For the top 400 DEGs out of the 849 DEGs, determined by cumulative AUCI_95%_ across all substances, an ORA was performed. This revealed effects on hypoxia gene sets, the development of eye, lens and retina and TNF alpha signaling via NFKB (Supplementary file 6). The temporal profiles of differential expression were visualized in a heatmap (Supplementary file 12).

Additionally, we performed an ORA for all DEGs per compound and found that the terms ‘intersegmental vessel’ (ZFA:0001285) or ‘post-vent vasculature’ (ZFA:0005037) (figure 6A) were significantly over-represented after the early exposure to sorafenib in the gene set obtained after 96 h. For the rotenone exposure, no terms were over-represented in the early exposure scenario. The terms of ‘vessel formation’ were also present in the sorafenib dataset of the late exposure scenario (24–72 hpf) with low p-values. Few other functions were significantly affected, including the ‘cholesterol and steroid metabolic process’ or ‘Cytochrome P450’. For the rotenone exposure a specific response to angiogenesis could not be observed, however ‘endoplasmic reticulum targeting’, ‘hormone activity’ and molecular response processes such as ‘histone folding’ were among the over-represented terms. Later stages (72–96 hpf) when exposure was initiated at 24 hpf revealed angiogenesis-related terms among the most significantly over-represented ones for sorafenib but not for rotenone. For the rotenone dataset, some retina- and brain-related gene sets were affected, however, *p*-values were generally high. A summary of the most significantly over-represented terms across all three substances is visualized in figure 6A. To select these terms, the top 7 terms, selected by lowest adjusted p-value, were taken from the ORAs from the intersections of the DEGs of both concentrations at all time frames and substances, resulting in 55 terms.

To obtain more detailed information about effects on specific vascular genes we analyzed the blood vasculature gene set of the ZFIN database (ZFA: 0001079) and the respective expression in zebrafish after exposures to the three substances. Figure 6D shows the expression of those possible biomarkers in every exposure condition over time. While the down-regulation of anti-angiogenic response after SU4312 exposure was strong downregulated in the late exposure scenarios, it might also be detected as significantly affected in the early exposure scenario, however only in the first 24 h after exposure and most of the time up-regulated. After 24 hpf, the regulation cannot be observed anymore. In the corresponding ORA, this response could easily be overlooked due to the large amount of other effects and gene sets affected in this time frame. Figure 6D also shows that that different genes of the blood vasculature gene set are affected in different time frames. While there are several genes that exhibit prolonged down-regulation in the late exposure with the TKIs (e.g. kdrl, clec14a, aqp8a.1) with almost no differential expression in the early exposure, there are genes that are regulated in the early but not in the late exposure scenario with SU4312 (e.g., ptgs1, micall2a, cavin1b), or genes that are regulated in opposite directions and at different ages of the embryo (e.g., si:ch211-14k19.8, mrc1a, kdr). While we describe this exemplarily for genes of the blood vasculature gene set, this was also observed for genes related to other biological functions. Rotenone exposure showed the lowest number of affect genes and terms over all scenarios, and only few angiogenesis-related genes were differentially expressed.

### Relation of gene expression and internal concentrations

**Fig. 7:**
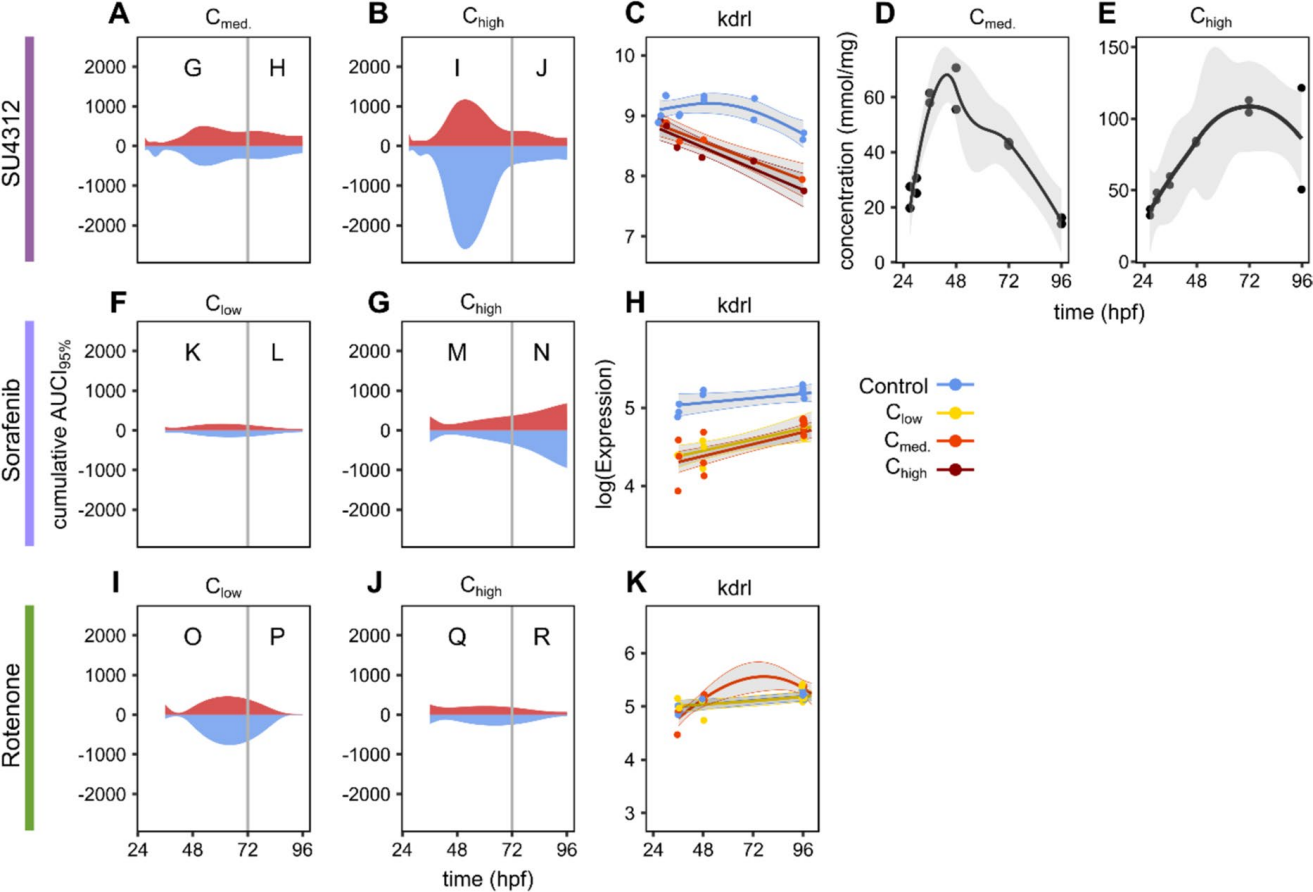
Global profile of temporal transcriptional changes and bioaccumulation following exposure to **A**–**E** SU4312, **F**–**H** sorafenib and **I**–**K** rotenone. Cumulative AUCI_95%_ profiles added for all genes. Areas above zero in red relate to up-regulated genes, areas below zero in blue to down-regulated genes. Bioaccumulation for late exposure condition (start at 24 hpf) of SU4312 over time. Expression profiles of *kdrl* in response to **C** SU4312, **H** sorafenib and **K** rotenone exposure. Colors indicate exposure concentrations

We measured the uptake SU4312 into the ZFE over time and compared the internal concentration time course with the gene expression patterns (Fig. 7, Supplementary file 17). The analysis was limited to SU4312 due to weak sensitivity of the analytical protocols of the other compounds. SU4312 showed a faster uptake with a peak between 48 and 72 hpf and a subsequent reduction of internal concentration (Fig. 7D, E). Comparing the gene expression patterns of all affected genes with the internal concentration dynamics indicates a limited relation. SU4312 showed the highest number of differentially expressed genes within 48 and 72 hpf and a decline afterwards (Fig. 7A, B). This aligns with the dynamics of the measured respective internal concentration dynamics that show the highest concentrations in this time frame (Fig. 7D, E). As observed earlier for SU4312, it becomes obvious and remarkable also for sorafenib and rotenone, that up-regulation and down-regulation seem to always be balanced in the ZFE.

The TKIs, SU4312 and sorafenib are designed to bind to the human VEGFR2 with high affinity. As the structure of the kinase domain is conserved across species, it can be assumed that the TKIs also bind to the zebrafish receptor. The gene expression of *kdrl*, the zebrafish ortholog, measured after exposure with the two TKIs and with rotenone, a potential angiogenesis disruptor that does not directly bind to the receptor, is shown in Fig. 7. While no differential expression was observed in the early scenario with SU4312 (Supplementary file 3), a down-regulation with all concentrations was observed in the late exposure scenarios with SU4312 as well as with sorafenib (Fig. 7C and H). While SU4312 caused a down-regulation that is decreasing over the exposure duration, the effect of sorafenib on *kdrl* expression is rather stable over time. No significant effect was observed with rotenone on the expression of *kdrl* (Fig. 7K). Next to the interference of chemicals with the proteins and respective pathways, such expression patterns can also be determined by the internal concentrations of compounds and their toxicokinetic properties.

## Discussion

The effects of chemicals are mediated via an initial molecular interaction and are translated via key events to adverse outcomes. This principle, as described by the adverse outcome pathway (AOP) concept (Ankley et al. 2018), allows to predict adverse effects from the measurement of key events. Various molecular initiating events often converge in single key events and assessment of the molecular responses, e.g., via transcriptional profiling, can support in unraveling the underlying mechanisms and increase confidence in the key events and their translation into adverse effects. Furthermore, transcriptional responses could represent biomarkers for the involved key events (Saarimäki et al. 2023). Transcriptional responses can be very dynamic and may change as a function of exposure concentration and time. Most transcriptomic studies perform static exposure scenarios, where only one time point and additionally, long exposure durations are used and the single transient transcriptomic pattern is then measured at the end of the exposure period (Oh et al. 2014; Gloss et al. 2017). Our results showed that transcriptional responses change dynamically and that focus on a selected stage of analysis may not reveal the responses reflecting the mechanism of action and could lead to incorrect conclusions. The method developed and applied here, makes use of generalized additive models (GAMs), which are flexible enough to capture dynamics of gene expression over time, while also yielding informative model parameters should they be of interest. Other methods to analyze time series gene expression data often use an F-statistic on the relevant coefficients of a model to determine differential expression, and while this results in a list of DEGs, it does not give information on directionality or exact time frames of regulation (Oh and Li 2021; Michna et al. 2016). Existing methods that detect time frames of differential gene expression are not implemented in gene expression analysis tools and are not further developed (Heinonen et al. 2015). By calculating average differences over time between control and treatment curves from GAMs, it allowed us to determine differential expression with high temporal resolution as well as to compare up- and down-regulation over time. Several studies have highlighted the dynamic transcriptomic approach during zebrafish embryogenesis (Aanes et al. 2014; Rauwerda et al. 2017), however, only few studies explain the dynamic changes after chemical exposure (Schüttler et al. 2019).

Therefore, in this study, we investigated the responses of the zebrafish transcriptome over time using anti-angiogenesis as a case study for the assessment of potential teratogenic effects. Zebrafish embryos were exposed to the model anti-angiogenic TKIs SU4312, sorafenib. Despite their primary design to bind VEGFR2, sorafenib and SU4312 are multi-targeted kinase inhibitors with affinities for other targets, such as PDGFR, EGFR, HER-2, IGF (SU4312), and FGFR1, KIT, and c-KIT (sorafenib) (Gotink and Verheul 2010; Sun et al. 1998). Both TKIs are categorized as type 2 kinase inhibitors, and indirectly compete with ATP by occupying the hydrophobic ATP binding pocket (Sun et al. 1998). We observed similarities and differences in transcriptomic responses driven by different exposure scenarios. Similarities were noted between SU4312 and sorafenib in the late exposure scenario but not in the early exposure scenario, likely due to their above-mentioned differing binding affinities.

The magnitude and type of chemical effects in a developing organism can largely depend on the time window of exposure (Wilson 1965). In an earlier study, we observed that exposure to anti-angiogenic chemicals caused less gross morphological effects and lethality, when the exposure is induced after the first 24 h of development (Nöth et al. 2024), yet inhibition of angiogenesis was prominently affected regardless of exposure start time. We hypothesized that transcriptional profiling with later exposure start times would result in more specific effects due to weaker confounding changes in the transcriptome. Indeed, the transcriptional changes in the late exposure scenario showed a stronger association with the known anti-angiogenic mode of action and less unspecific effects, and hence, confirms the specificity that was observed earlier with the morphological effects. Comparing the functional terms in the SU4312 exposure duration from 2 to 24 hpf with a meta-analysis of 60 compounds targeting different modes of action (Schüttler et al. 2017) showed similar functional terms related to homeobox, musculature, and crystallin as in our findings. Especially within the first 24 h axis patterning, germ layer formation, and organogenesis is taking place and is a highly conserved mechanism. Additionally, dynamic changes in metabolites were observed between early (2hpf) and 24 hpf old zebrafish and are clearly stage-specific during further development (Dhillon et al. 2019). Disruption of these conserved developmental processes might result in similar developmentally toxic profiles regardless of the compounds’ mode of action. Although we observed an anti-angiogenic response after an early sorafenib exposure, it was not observed after an early exposure to SU4312. Therefore, we concluded that this exposure design has a limited capacity to indicate inhibition of angiogenesis.

We compared the responses of the TKI inhibitors to the insecticide rotenone, a potential inhibitor of angiogenesis (McCollum et al. 2017). The zebrafish embryo model indicates only a mild inhibition of angiogenesis in the tested concentration range (Supplementary file 2). However, we observed additional morphological changes similar to those observed by Melo et al. (2015) who described cardiac edema, tail deformities, loss of equilibrium, and a general developmental delay characterized by lack of tail detachment, delayed somite formation, yolk sac absorption, and lack of pigmentation. The known major mode of action of rotenone is the inhibition of complex I in the electron transport chain of mitochondria (Li et al. 2003). It causes a mild depletion on ATP-level, oxidative stress and death caused by specific interaction to complex I. rotenone-induced oxidative stress is described to play a role in angiogenesis (Lu et al. 2015). This suggests a potential link between ATP depletion and angiogenesis disruption. Furthermore, the production of reactive oxygen species (ROS), which is influenced by ATP levels, is involved in angiogenesis (Li et al. 2003). This agrees with our data, as we observed differentially expressed genes that are connected to oxidative stress and GTH depletion after rotenone exposure. However, only few blood-vessel-specific genes were significantly differentially expressed after rotenone exposure, indicating that the anti-angiogenic effect may rather represent a secondary effect.

Transcriptional change can also reflect changes in the internal concentrations. Peak concentration and a decline with prolonged exposure indicate an induction of biotransformation (Kühnert et al. 2013; Edwards et al. 2002). This was indicated by the internal concentration profile of SU4312, supported by the increase in gene expressions related to metabolic pathways (liver X receptor (LXR) and pregnane X receptor (PXR)) and a strong up-regulation of CYP1a. Especially sorafenib and rotenone show activation of LXR and PXR metabolic pathways. They are involved in transcription of lipid metabolism and inflammatory responses with a main function in liver protection (Timsit and Negishi 2007; Zelcer and Tontonoz 2006). For some TKIs a metabolization via the cytochrome P450 has been described (Hartmann et al. 2009). For example, sorafenib and sunitinib have been shown to be metabolized via CYP3A4, a target gene of the PXR, while the parent compound and active metabolite have similar biochemical activity and potency (Li et al. 2003; Houk et al. 2009). The changes in the expression profiles, particularly reduced magnitude of the responses, hence, may partially also reflect the decline in internal concentrations, but it is difficult to disentangle between a time-dependent expression dynamics and concentration-dependent effects.

CYP1a expression does start around 24 hpf in developing zebrafish (Saad et al. 2016; Kühnert et al. 2017), and the highest mRNA levels can be found in the cardiovascular system (Otte et al. 2010). This indicates a connection between CYP1a expression and the vascular system is underlined, e.g., by observations with TCDD exposures that induced CYP1A levels and vascular malformations in early zebrafish development. Nevertheless, CYP1A is known as a general response after xenobiotic chemical exposure (Esteves et al. 2021) and therefore not a suitable angiogenesis specific biomarker candidate.

We observed changing numbers of up- and down-regulated DEGs over time. This might be due to fast changes of transcriptional patterns during early embryogenesis (Samrani et al. 2023). In this study, two basic expression patterns can often be observed. First impulse patterns where up- or down-regulation is followed by a return to basal levels over time. The SU4321 early exposure showed this type of expression for several genes within the first 24 h of exposure. Second, in sustained patterns the gene remains up- or down-regulated mainly observed in late exposure scenarios of SU4312, sorafenib and rotenone. These findings reflect the complex and highly dynamic gene expression responses after chemical exposure, which can only be observed with a time-resolved study design. Furthermore, the internal concentrations of our test compounds and the number and level of response of DEGs appear to be correlated. The decreases of SU4312 and rotenone concentrations, potentially due to the above described metabolization, led to a decline of DEGs with longer exposure durations. The uptake of sorafenib was slower, and hence, the highest number of DEGs was observed at 96 hpf. How the biotransformation and potential transformation products influence the overall gene expression, the angiogenesis disruption, and specific transcriptomic responses needs further investigation. Comparing up- and down-regulation over time, we observed that the number of genes differentially expressed in both directions were typically balanced (see also Figs. 3 and 7). Exceptions were seen within the first 24 hpf following the SU4312 early exposure where more up-regulated than down-regulated genes were found, while the peak around 48 hpf in the late exposure scenario was dominated by down-regulated genes. Potentially, this may be a mechanism in an organism with a finite amount of available energy, where the up-regulation of chemical stress response processes is compensated by the down-regulation of developmental processes that are set on hold. Indeed, developmental delay is one of the most common phenotypic morphological observations in zebrafish embryos after chemical exposure (Teixidó et al. 2013). So far, this effect on the transcriptome has not yet been described and requires further investigations as it provides the potential to separate specific and unspecific responses in more detail in future.

Our data indicate that transcriptome analysis in the zebrafish embryo model has the potential to support the identification of the key event “Reduction of angiogenesis” (https://aopwiki.org/aops/43) and enable to identify interaction of chemicals of high relevance for human health. Mainly unspecific developmental toxicity in the early exposure scenario was observed, while a specific repression of vascular related genes could be identified clearly in the late exposure scenario. If the angiogenesis effect is a secondary effect, caused by another mode of action, the prediction of anti-angiogenesis biomarkers is limited. Furthermore, transcriptional responses are highly dynamic and identification of specific response patterns linked to key events depend on the selected exposure window and require a time resolved assessment. This study used anti-angiogenesis as proof of principle but the findings—with different sets of transcriptional responses—may apply for other mode of actions as well.

## Conflict of interest

The authors declare no conflict of interest.

## Supplementary Information

Below is the link to the electronic supplementary material.

Supplementary file1 (HTML 4466 KB)

Supplementary file2 (PNG 1632 KB)

Supplementary file3 (PNG 92 KB)

Supplementary file4 (PNG 58 KB)

Supplementary file 5 (TSV 4466 KB)

Supplementary file 6 (TSV 4466 KB)

Supplementary file 7 (TSV 4466 KB)

Supplementary file8 (XLSX 1148 KB)

Supplementary file9 (XLSX 3009 KB)

Supplementary file10 (XLSX 1784 KB)

Supplementary file11 (XLSX 21502 KB)

Supplementary file12 (PNG 1472 KB)

Supplementary file13 (XLSX 5366 KB)

Supplementary file 14 (TSV 4466 KB)

Supplementary file15 (XLSX 99 KB)

Supplementary file 16 (TSV 4466 KB)

Supplementary file 17 (TSV 4466 KB)

## Data Availability

Transcriptomic datasets generated for this study were submitted to GEO (Accessions: GSE270294 and GSE270785). All other data is published as part of the supplement of this publication. All code used to conduct the analyses is available at https://codebase.helmholtz.cloud/ufz/tb3-cite/etox/itox/2022_zfaop_omics.
