# Correction to: Dynamics in zebrafish development define transcriptomic specificity after angiogenesis inhibitor exposure

**DOI:** 10.1007/s00204-025-03998-1

**Published:** 2025-03-19

**Authors:** Julia Nöth, Paul Michaelis, Lennart Schüler, Stefan Scholz, Janet Krüger, Volker Haake, Wibke Busch

**Affiliations:** 1 Department of Ecotoxicology, Helmholtz Centre for Environmental Research—UFZ, Permoserstraβe 15, 04318 Leipzig, Germany; 2 Department of Monitoring and Exploration Technologies, Helmholtz Centre for Environmental Research—UFZ, Permoserstraβe 15, 04318 Leipzig, Germany; 3 BASF Metabolome Solutions GmbH, Tegeler Weg 33, 10589 Berlin, Germany

**Correction to: Archives of Toxicology** 10.1007/s00204-024-03944-7

In this article, the citation of supplementary information file were incorrect and should have been as given below:

supplementary_methods.html should have been Supplementary file 1

SI_fig2_Rotenone should have been Supplementary file 2

SI_fig4_kdrl) should have been Supplementary file 3

SI_fig1_cost_function_DEG should have been Supplementary file 4

SI_tab10_SUSORO_intersection_DEGs) should have been Supplementary file 5

SI_tab11_SUSORO_late_intersection_top400_enrichment should have been Supplementary file 6

SI_tab4_SU_top500_ should have been Supplementary file 7

(SI_tab9_SORO_DEGs) should have been Supplementary file 8

SI_tab3_SU4312_DEGs should have been Supplementary file 9

SI_tab7_enr ichments_ORA_gam_Rotenone_ Sorafenib should have been Supplementary file 10

SI_tab6_enrichments_ORA_SU4312 should have been Supplementary file 11

SI_fig3_SUSORO_intersection_heatmap_top should have been Supplementary file 12

(SI_tab2_SUSORO_DEG_summary should have been Supplementary file 13

SI_tab8_SUSORO_no_of_degs_terms) should have been Supplementary file 14

SI_tab5_SU4312_top500_cluster_enrichments should have been Supplementary file 15

Supplementary file 16 is removed from the Original article.

SI_tab1_internal_dose_SU4312 should have been Supplement file 16

The original article has been corrected.

