# Supplementary material for: Dynamics in zebrafish development define transcriptomic specificity after angiogenesis inhibitor exposure
Source: Arch Toxicol. 2025 Jan 9;99(4):1561–78. doi: 10.1007/s00204-024-03944-7 (PMC11968557; doi:10.1007/s00204-024-03944-7)
Supplement: Supplementary file 1 — Supplementary file1 (HTML 4466 KB) [file 204_2024_3944_MOESM1_ESM.html]

Supplementary methods to “Dynamics in Zebrafish Development define Transcriptomic Specificity after Angiogenesis Inhibitor Exposure”


Code 

- Show All Code
- Hide All Code

# Supplementary methods to “Dynamics in Zebrafish Development define Transcriptomic Specificity after Angiogenesis Inhibitor Exposure”

#### Paul Michaelis, Julia Nöth, Janet Krüger, Stefan Scholz, Volker Haake, Stephan Schreiber, Lennart Schüler, Wibke Busch

```
library(limma)
library(mgcv)
library(splines)
library(enrichplot)
library(data.table)
library(igraph)
library(RCy3)
library(reticulate)
library(tidyverse)
library(DESeq2)
library(openxlsx)
library(ggh4x)
library(PoiClaClu)
library(gplots)
library(heatmap3)
library(cowplot)
library(eulerr)
library(reticulate)

knitr::opts_chunk$set(echo = T)
knitr::opts_chunk$set(message = F)
knitr::opts_chunk$set(warning = F)
knitr::opts_chunk$set(fig.align = 'center')
knitr::opts_knit$set(root.dir = '../')
knitr::knit_engines$set(python = reticulate::eng_python)
use_python('/usr/bin/python3')
```

```
t_theme = function(l_size = 1,
                   t_size = 12,
                   face = 'plain',
                   family = NULL,
                   legend_position = 'bottom',
                   title_size = 12) {
  
  t = element_text(size = t_size, colour = 'black', face = face, family = family)
  theme_classic() +
  theme(
    axis.line = element_blank(),
    axis.ticks = element_line(linewidth = 0.5),
    axis.text = t,
    axis.title = t,
    plot.title = element_text(face = face, size = title_size, family = family),
    legend.position = legend_position,
    legend.title = t,
    legend.text = t,
    strip.text = t,
    panel.border = element_rect(fill = NA, colour = 'black', size = l_size),
    panel.grid = element_blank(),
    strip.background = element_rect(fill = NA, colour = 'black', size = l_size),
    panel.background = element_blank(),
    axis.ticks.length = unit(0.6, 'mm')
  )
}
theme_set(t_theme())

# adds a worksheet to an excel file and writes a table into it (uses openxlsx)
add_data = function(wb, sheet_name, x) {
  addWorksheet(wb, sheet_name)
  writeData(wb, sheet_name, x = x)
}

# colours for plotting
conc_cols = list(C0 = 'cornflowerblue', C1 = 'gold1', C2 = 'orangered2', C3 = 'darkred')
conc_cols_vector = c(C0 = 'cornflowerblue', C1 = 'gold1', C2 = 'orangered2', C3 = 'darkred')
substance_cols = c(SU4312 = 'mediumorchid4', Sorafenib = 'lightslateblue', Rotenone = 'chartreuse4')
```

# 

## About

This document outlines the computational analysis for the paper
**Dynamics of Zebrafish Development after Angiogenesis Inhibitor
Exposure**. In this study, temporal differential expression
patterns for SU4312, Sorafenib (both tyrosine kinase inhibitors, binding
to the kinase domain of VEGFR2) and Rotenone (an insecticide and
potential vascular disruptor) were compared amongst each other and
across different exposure scenarios and concentrations. The document is
structured in four parts:

- In *Modelling* generalized additive models (GAMs) are
  computed for appropriate time-series data and differentially expressed
  genes (DEGs) are determined.
- *Global analysis* visualizes whole-transciptome expression
  patterns and bio-accumulation data
- *Enrichments* performs over-representation analyses (ORAs)
  for sets of DEGs and visualizes the results
- *Information* contains used packages and their versions

## Modelling

### SU4312 data

SU4312 Elists are loaded. There are separate ELists for early and
late exposure samples, as well as logFC ELists where logFC have been
calculated with respect to the mean of the relevant controls.

Probes with highest IQR are chosen as representatives of genes when
multiple probes map to the gene.

```
iqr = data_0$genes %>% 
  dplyr::select(ProbeName, ensembl_gene_id, external_gene_name) %>% 
  add_column(
    iqr_0 = data_0$E %>% apply(1, IQR),
    iqr_24 = data_24$E %>% apply(1, IQR)
  )
iqr = iqr %>% drop_na(ensembl_gene_id) %>% group_by(ensembl_gene_id) %>% 
  summarise(
    probe_0 = ProbeName[which.max(iqr_0)],
    probe_24 = ProbeName[which.max(iqr_24)]
  )

saveRDS(iqr, 'data/iqr_probes.rds')
```

Subsetting the ELists.

```
data_0 = data_0[rownames(data_0) %in% iqr$probe_0, ]
rownames(data_0) = data_0$genes$ensembl_gene_id
data_24 = data_24[rownames(data_24) %in% iqr$probe_24, ]
rownames(data_24) = data_24$genes$ensembl_gene_id

data_0_logfc = data_0_logfc[rownames(data_0_logfc) %in% iqr$probe_0, ]
rownames(data_0_logfc) = data_0_logfc$genes$ensembl_gene_id
data_24_logfc = data_24_logfc[rownames(data_24_logfc) %in% iqr$probe_24, ]
rownames(data_24_logfc) = data_24_logfc$genes$ensembl_gene_id
```

Several functions for fitting and plotting the models, and other
viualisations are loaded.

```
source('scripts/functions/fit_splines.R')
source('scripts/functions/enrichment.R')
source('scripts/functions/visualisations.R')
```

The Generalized Additive Models (GAMs) are fitted for both datasets.
`fit_gam_batch()` uses `mgcv::gam()` to model each
gene’s expression over time for each concentration level. *k*, as
the parameter defining the number of dimensions of the basis of the
smooth term of the GAMs is chosen to be the same as the number of time
points in the data.

```
fits_0_gam = fit_gam_batch(data_0, rownames(data_0), k = 10)
saveRDS(fits_0_gam, file = 'data/gam/gam_fits_0_probes.rds')

fits_24_gam = fit_gam_batch(data_24, rownames(data_24), k = 6)
saveRDS(fits_24_gam, file = 'data/gam/gam_fits_24_probes.rds')
```

The models calculated above are used to simulate 10.000 curves for
each model, using model parameters pulled from a multivariate normal
distribution according to estimated parameters of the original model.
Treatment curves are then subtracted from control curves to calculate
the simulated mean difference to the control over time, as well as its
95% confidence interval (CI).

```
diff_0_gam = gam_sim_diff_batch(data_0, rownames(data_0), k = 10)
saveRDS(diff_0_gam, 'data/gam/gam_diff_0_probes.rds')

diff_24_gam = gam_sim_diff_batch(data_24, rownames(data_24), k = 6)
saveRDS(diff_24_gam, 'data/gam/gam_diff_24_probes.rds')
```

Using the CIs from the simulations, the difference of the lower band
of the CI (or upper band if the gene is down-regulated) to zero is
calculated at each hour in the exposure duration. These values can then
be aggregated to get the AUCI95%, the area under the 95% CI,
which is used as an effect measure in this study.

```
ci_diff_0_gam = get_sim_CI_diffs(diff_0_gam, rownames(data_0)) %>% 
  add_column(exposure = 'early') %>% 
  add_column(substance = 'SU4312') %>% 
  add_column(ensembl_gene_id = .$gene)
saveRDS(ci_diff_0_gam, 'data/gam/gam_ci_diff_0.rds')

ci_diff_24_gam = get_sim_CI_diffs(diff_24_gam, rownames(data_24)) %>% 
  add_column(exposure = 'late') %>% 
  add_column(substance = 'SU4312') %>% 
  add_column(ensembl_gene_id = .$gene)
saveRDS(ci_diff_24_gam, 'data/gam/gam_ci_diff_24.rds')
```

Gene annotation is added.

```
ci_diff_0_gam = ci_diff_0_gam %>% 
  left_join(data_0$genes[, c('ProbeName', 'ensembl_gene_id', 'external_gene_name')],
            by = c('gene' = 'ensembl_gene_id'))
ci_diff_24_gam = ci_diff_24_gam %>%
  left_join(data_0$genes[, c('ProbeName', 'ensembl_gene_id', 'external_gene_name')],
            by = c('gene' = 'ensembl_gene_id'))
```

Visualisation of the data using principal component analysis. Samples
cluster by time point instead of concentration level, indicating this is
the strongest variable determining expression.

```
pca_data = function(elist) {
    pca = prcomp(t(na.omit(elist$E)))
    df = pca$x[, 1:3] %>% as_tibble()
    df
}

pca_0 = pca_data(data_0) %>% 
  add_column(time_hpf = data_0$targets$time_hpf,
             exposure = as.factor(data_0$targets$start_hpf),
             c_lvl = data_0$targets$concentration_level)

pca_24 = pca_data(data_24) %>% 
  add_column(time_hpf = data_24$targets$time_hpf,
             exposure = as.factor(data_24$targets$start_hpf),
             c_lvl = data_24$targets$concentration_level)

plot_grid(
  pca_0 %>% ggplot(aes(x = PC1, y = PC2, colour = factor(time_hpf), label = c_lvl)) +
    geom_point(size = 5) +
    geom_text(colour = 'black', size = 3) +
    ggtitle('PCA SU4312 early exposure'),
  pca_24 %>% ggplot(aes(x = PC1, y = PC2, colour = factor(time_hpf), label = c_lvl)) +
    geom_point(size = 5) +
    geom_text(colour = 'black', size = 3) +
    ggtitle('PCA SU4312 late exposure'),
  ncol = 2
)
```

The model for the late exposure of cyp1c1 is visualised below.
Compared to the control curve in blue, in the SU4312 treatment cyp1c1 is
up-regulated for most of the exposure duration. The second subfigure
shows the mean difference over time from the simulations and the hatched
area signifies the AUCI95%.

```
g = 'ENSDARG00000101195' # cyp1c1
c_lvl = 'C2'
plot = cowplot::plot_grid(
  plot_gam(g, data_24, c_lvl, start_hpf = 24, time = 'time_hpe') + ggtitle('') + theme(legend.position = 'none'),
  plot_gam_diff(g, data_24, c_lvl) +
    ggpattern::geom_area_pattern(
      data = ci_diff_24_gam[ci_diff_24_gam$gene == g &
                             ci_diff_24_gam$contrast == c_lvl,],
      mapping = aes(x = t, y = diff),
      stat = 'identity',
      fill = 'white',
      colour = 'grey70',
      # width = 1,
      pattern = 'stripe',
      pattern_density = 0.05,
      pattern_fill = 'grey70',
      pattern_spacing = 0.02,
      pattern_angle = -45
    ) +
    ggtitle('') +
    theme(legend.position = 'none'),
  labels = LETTERS[1:2],
  scale = 0.95
)

ggsave('results/figures_final/fig0_gam_example/splines_example_cyp1c1_C2.svg', plot = plot, 'svg', width = 18, height = 8, units = 'cm', bg = 'white')

plot
```

The CI differences calculated above are summarised. For each gene and
concentration of SU4312 these tables contain the AUCI95%, the
AUCI95%/h, which is the AUCI95% normalised with
the exposure duration in hours, the duration of differential expression,
the time point at maximal differential expression and the direction of
differential expression.

```
sig_summ_0_p = ci_diff_0_gam %>% 
  filter(diff != 0) %>% 
  group_by(gene, contrast) %>% 
  summarise(
    auci = sum(diff),
    auci_per_h = sum(diff) / 93,
    duration = sum(diff != 0),
    tmax = t[which.max(abs(diff))],
    direction = diff[which.max(abs(diff))] %>% sign(),
    .groups = 'drop'
  ) %>% 
  left_join(data_0$genes[, c('ProbeName', 'ensembl_gene_id', 'external_gene_name')],
            by = c('gene' = 'ensembl_gene_id'))

sig_summ_24_p = ci_diff_24_gam %>% 
  filter(diff != 0) %>% 
  group_by(gene, contrast) %>% 
  summarise(
    auci = sum(diff),
    auci_per_h = sum(diff) / 69,
    duration = sum(diff != 0),
    tmax = t[which.max(abs(diff))],
    direction = diff[which.max(abs(diff))] %>% sign(),
    .groups = 'drop'
  ) %>% 
  left_join(data_0$genes[, c('ProbeName', 'ensembl_gene_id', 'external_gene_name')],
            by = c('gene' = 'ensembl_gene_id'))

saveRDS(sig_summ_0_p, 'data/gam/gam_sig_summ_0.rds')
saveRDS(sig_summ_24_p, 'data/gam/gam_sig_summ_24.rds')
```

To determine differentially expressed genes a cutoff on the
AUCI95%/h is chosen. To do this, a random selection treatment
and control curves were generated and labelled as differentially
expressed or not differentially expressed by visual inspection. With
this boolean variable and the associated AUCI95%/h values,
one can determine an optimal cutoff for the AUCI95%/h,
i.e. the AUCI95%/h where the fewest curves are wrongly
classified. Since there is only one variable over which to optimise this
function, this was done using a brute force algorithm.

```
samples_0 = sig_summ_0_p %>% dplyr::select(gene, contrast, auci_per_h) %>% 
  slice_sample(n = 150) %>% 
  add_column(id = paste(.$gene, .$contrast, sep = '.')) %>% 
  add_column(exp_start = 0)
samples_24 = sig_summ_24_p %>% dplyr::select(gene, contrast, auci_per_h) %>% 
  slice_sample(n = 150) %>% 
  add_column(id = paste(.$gene, .$contrast, sep = '.')) %>% 
  add_column(exp_start = 24)

write_tsv(samples_0, 'data/find_cutoff/sample_0.tsv')
write_tsv(samples_24, 'data/find_cutoff/samples_24.tsv')

pdf('data/find_cutoff/sample_0.pdf')
for (g in samples_0$gene) {
  print(plot_gam(g, data_0, c('C0', as.character(samples_0$contrast[samples_0$gene == g]))))
}
dev.off()
pdf('data/find_cutoff/sample_0.pdf')
for (g in samples_24$gene) {
  print(plot_gam(g, data_24, c('C0', as.character(samples_24$contrast[samples_24$gene == g]))))
}
dev.off()
```

```
#!/usr/bin/env python3
from pathlib import Path
import numpy as np
import pandas as pd
from scipy.optimize import brute
import matplotlib.pyplot as plt

VERBOSE = False
PATH = Path('data/find_cutoff/')
FILENAME_0 = PATH / 'sample_0.tsv'
FILENAME_24 = PATH / 'sample_24.tsv'

# initial cutoff guess
CUTOFF_0 = 0.01
# cutoff bounds for the estimation
BOUNDS = ((0.0, 1.0),)
# step size (calc. as `CUTOFF_0 * DIFF_STEP`)
# important for descrete problems, like this one
DIFF_STEP = 0.01

def read_data(filename: Path) -> pd.DataFrame:
    """Read data from tsv file `filename`."""
    df = pd.read_csv(filename, sep='\t')
    df['is_sig'] = df['is_sig'].map({'T': True, 'F': False})
    return df

def test_cutoff(
    cutoff: list,
    values: np.ndarray,
    significance: np.ndarray
) -> float:
    """Calculate no. of wrong significances, given `cutoff`.

    The fct. `brute` minimizes functions, therefore this fct. returns
    the no. of wrong significances.
    """
    if type(cutoff) != type([1]):
        cutoff = [cutoff]
    return float(len(values) - ((values > cutoff[0]) == significance).sum())

def post_processing(df, cutoff, verbose):
    wrong_sig = df[~(df.ci_per_h > cutoff) == df.is_sig]
    print(
        f'{len(wrong_sig)}/{len(df)} significance '
         'values could not be matched with optimal cutoff value.'
    )
    if verbose:
        print(f'Not matching:\n{wrong_sig}')

def manual_fit(df: pd.DataFrame) ->  None:
    """Manually test different cutoff values, just for testing."""
    min_c = np.nan
    min_value = len(df.ci_per_h)
    # adjust values here
    for c in np.arange(0, 0.3, 0.001):
        val = test_cutoff([c], df.ci_per_h, df.is_sig)
        if val < min_value:
            min_c = c
            min_value = val
    
    print(f'Min value = {min_value}, Best cutoff value: {min_c:.3}')

def get_opt_cutoff(cutoff_start, bounds, df):
    """Wrapper for least squares method"""
    res = brute(
            test_cutoff,
            ranges=bounds,
            args=(np.array(df.ci_per_h), np.array(df.is_sig))
    )
    return res[0]

dfs = {
    'Early Exposure': read_data(FILENAME_0),
    'Late Exposure': read_data(FILENAME_24),
}
# append the df's and add another entry to dict `dfs` to test all
dfs['All'] = pd.concat(dfs.values()).reset_index(drop=True)

for n in dfs:
    print(n)
    res = brute(
        test_cutoff,
        ranges=BOUNDS,
        args=(np.array(dfs[n].ci_per_h), np.array(dfs[n].is_sig)),
    )
    cutoff_opt = res[0]

    print(f'Optimal cutoff value: {cutoff_opt:.3}')
    post_processing(dfs[n], cutoff_opt, VERBOSE)
    print()
```

```
## Early Exposure
## Optimal cutoff value: 0.05
## 34/150 significance values could not be matched with optimal cutoff value.
## 
## Late Exposure
## Optimal cutoff value: 0.0474
## 20/150 significance values could not be matched with optimal cutoff value.
## 
## All
## Optimal cutoff value: 0.0467
## 53/300 significance values could not be matched with optimal cutoff value.
```

```
# data frame with a sequence of potential starting cutoffs
opt_cutoff_df = pd.DataFrame()
opt_cutoff_df['cutoff_0'] = np.arange(0.0001, 0.5, 0.001)
# optimal cutoffs are calculated for the sequence of starting values
opt_cutoff_df['opt_cutoff'] = opt_cutoff_df['cutoff_0'].apply(
    get_opt_cutoff,
    args = (BOUNDS, dfs['All'])
)
# number of wrongly classified curves using the sequence of starting values as cutoffs
opt_cutoff_df['class_wrong_n'] = opt_cutoff_df['cutoff_0'].apply(
    test_cutoff,
    args = (np.array(dfs['All'].ci_per_h), np.array(dfs['All'].is_sig))
)
# getting the cutoffs with the lowest number of misclassified curves
min_cutoffs = list(opt_cutoff_df['cutoff_0'][opt_cutoff_df['class_wrong_n'] == 
    min(opt_cutoff_df['class_wrong_n'])])

# plot of starting guesses vs number of wrongly classified curves
plt.figure('Wrongly classified curves vs. cutoff')
plt.plot(opt_cutoff_df['cutoff_0'], opt_cutoff_df['class_wrong_n'])
plt.xlabel('cutoff')
plt.ylabel('number of wrongly classified curves')
plt.axvspan(
    xmin = min_cutoffs[0],
    xmax = min_cutoffs[len(min_cutoffs)-1],
    alpha = 0.5
)
plt.annotate(
    f'Optimal cutoff range: {min_cutoffs[0]:.3} to {min_cutoffs[len(min_cutoffs)-1]:.3}',
    (0.1, 150.0)
)
plt.savefig('results/figures/optimal_AUCI_cutoff.png')
```

Considering that the calculated optimal cutoffs for the early, late
and combined exposures are very similar, a single cutoff of 0.0467
|AUCI95%/h| is chosen to determine significance.

```
c = 0.0467
```

### Sorafenib & Rotenone

The data analysis of the Sorafenib and Rotenone datasets largely
mirrors that of the SU4312 data. A difference is the initial analysis
with DESeq2, as the reduced experimental design for the 0 hpf exposure
does not allow modelling over time.

#### **DESeq2**

The counts and sample information is loaded.

```
# annotation
ma_genes = data_0$genes %>% dplyr::select(ensembl_gene_id, external_gene_name) %>% unique()

samples = read_tsv('data/rotenone_sorafenib/rot_sora_samples.tsv') %>% 
  # this sample (DMSO, early, 96h) only has 19 reads, should be fine as there are 3 replicates left
  filter(!LibraryID %in% c('L119346'))

counts = read_tsv('data/rotenone_sorafenib/counts.tsv') %>% as.data.frame()
genes = counts[, 1]
counts = counts[, -1]
rownames(counts) = genes

samples$c = paste(samples$substance, samples$exposure_scenario, samples$concentration_level, samples$time_hpf, sep = '_') %>%
  as.factor()
d.all = DESeqDataSetFromMatrix(
  countData = counts[, samples$LibraryID],
  colData = samples,
  design = ~ 0 + c
)
```

A PCA is done. Again, most of the variance is determined by time.
Samples form three distinct clusters for each time point.

```
pca_data = function(deseq) {
    pca = prcomp(t(vst(counts(deseq))))
    df = pca$x[, 1:3] %>% as_tibble()
    df = cbind(df, colData(deseq))
    df
}

df = pca_data(d.all)

df %>% ggplot(aes(
    x = PC1,
    y = PC2,
    colour = substance,
    shape = exposure_scenario,
    label = paste(concentration_level, time_hpf, LibraryID, sep = '\n')
  )) +
  geom_point(size = 2) +
  geom_text()
```

However, it is clear that sample `L119368` is an outlier,
it is removed before further analysis. The DESeq object is modelled
again, without this sample. In the sample file a ‘condition’ column is
created as the combination of substance, exposure start, concentration
level and observation time. This is used to define groups for the design
of the model.

```
samples = samples %>% dplyr::filter(!LibraryID %in% 'L119368')

samples$c = paste(samples$substance, samples$exposure_scenario, samples$concentration_level, samples$time_hpf, sep = '_') %>%
  as.factor()

d.all = DESeqDataSetFromMatrix(
  countData = counts[, samples$LibraryID],
  colData = samples,
  design = ~ 0 + c
)
```

DESeq fits the model and contrasts of interest are defined. These are
treatment vs. control within the respective substance, time point and
exposure scenario. Additionally, contrasts that compare the mean of the
two treatment concentrations to the control are defined.

```
d.all = DESeq(d.all)

mean_contrasts = makeContrasts(
  (cRotenone_late_C1_36 + cRotenone_late_C2_36)/2 - cDMSO_late_C0_36,
  (cRotenone_late_C1_48 + cRotenone_late_C2_48)/2 - cDMSO_late_C0_48,
  (cRotenone_late_C1_96 + cRotenone_late_C2_96)/2 - cDMSO_late_C0_96,
  (cSorafenib_late_C1_36 + cSorafenib_late_C2_36)/2 - cDMSO_late_C0_36,
  (cSorafenib_late_C1_48 + cSorafenib_late_C2_48)/2 - cDMSO_late_C0_48,
  (cSorafenib_late_C1_96 + cSorafenib_late_C2_96)/2 - cDMSO_late_C0_96,
  levels = resultsNames(d.all)
)

deseq_to_df = function(data, genes) {
  data %>%
    as.data.frame() %>%
    add_column(ensembl_gene_id = rownames(.)) %>%
    left_join(genes, by = 'ensembl_gene_id') %>%
    arrange(padj, pvalue)
}

results_SR = list(
  # early results
  Sorafenib.e.96.C2 = results(d.all, contrast = list('cSorafenib_early_C2_96', 'cDMSO_early_C0_96'), alpha = 0.05) %>%
    deseq_to_df(ma_genes),
  Rotenone.e.96.C1 = results(d.all, contrast = list('cRotenone_early_C1_96', 'cDMSO_early_C0_96'), alpha = 0.05) %>%
    deseq_to_df(ma_genes),
  
  # late results
  Sorafenib.l.36.C1 = results(d.all, contrast = list('cSorafenib_late_C1_36', 'cDMSO_late_C0_36'), alpha = 0.05) %>% 
    deseq_to_df(ma_genes),
  Sorafenib.l.48.C1 = results(d.all, contrast = list('cSorafenib_late_C1_48', 'cDMSO_late_C0_48'), alpha = 0.05) %>% 
    deseq_to_df(ma_genes),
  Sorafenib.l.96.C1 = results(d.all, contrast = list('cSorafenib_late_C1_96', 'cDMSO_late_C0_96'), alpha = 0.05) %>% 
    deseq_to_df(ma_genes),
  
  Sorafenib.l.36.C2 = results(d.all, contrast = list('cSorafenib_late_C2_36', 'cDMSO_late_C0_36'), alpha = 0.05) %>% 
    deseq_to_df(ma_genes),
  Sorafenib.l.48.C2 = results(d.all, contrast = list('cSorafenib_late_C2_48', 'cDMSO_late_C0_48'), alpha = 0.05) %>% 
    deseq_to_df(ma_genes),
  Sorafenib.l.96.C2 = results(d.all, contrast = list('cSorafenib_late_C2_96', 'cDMSO_late_C0_96'), alpha = 0.05) %>% 
    deseq_to_df(ma_genes),
  
  Sorafenib.l.36.mean = results(d.all, contrast = mean_contrasts[, 4], alpha = 0.05) %>% 
    deseq_to_df(ma_genes),
  Sorafenib.l.48.mean = results(d.all, contrast = mean_contrasts[, 5], alpha = 0.05) %>% 
    deseq_to_df(ma_genes),
  Sorafenib.l.96.mean = results(d.all, contrast = mean_contrasts[, 6], alpha = 0.05) %>% 
    deseq_to_df(ma_genes),
  
  Rotenone.l.36.C1 = results(d.all, contrast = list('cRotenone_late_C1_36', 'cDMSO_late_C0_36'), alpha = 0.05) %>% 
    deseq_to_df(ma_genes),
  Rotenone.l.48.C1 = results(d.all, contrast = list('cRotenone_late_C1_48', 'cDMSO_late_C0_48'), alpha = 0.05) %>% 
    deseq_to_df(ma_genes),
  Rotenone.l.96.C1 = results(d.all, contrast = list('cRotenone_late_C1_96', 'cDMSO_late_C0_96'), alpha = 0.05) %>% 
    deseq_to_df(ma_genes),
  
  Rotenone.l.36.C2 = results(d.all, contrast = list('cRotenone_late_C2_36', 'cDMSO_late_C0_36'), alpha = 0.05) %>% 
    deseq_to_df(ma_genes),
  Rotenone.l.48.C2 = results(d.all, contrast = list('cRotenone_late_C2_48', 'cDMSO_late_C0_48'), alpha = 0.05) %>% 
    deseq_to_df(ma_genes),
  Rotenone.l.96.C2 = results(d.all, contrast = list('cRotenone_late_C2_96', 'cDMSO_late_C0_96'), alpha = 0.05) %>% 
    deseq_to_df(ma_genes),
  
  Rotenone.l.36.mean = results(d.all, contrast = mean_contrasts[, 1], alpha = 0.05) %>% 
    deseq_to_df(ma_genes),
  Rotenone.l.48.mean = results(d.all, contrast = mean_contrasts[, 2], alpha = 0.05) %>% 
    deseq_to_df(ma_genes),
  Rotenone.l.96.mean = results(d.all, contrast = mean_contrasts[, 3], alpha = 0.05) %>% 
    deseq_to_df(ma_genes)
)

lapply(names(results), function(n) {
  data.frame(
    name = n,
    DEGs_all = results[[n]] %>% filter(padj < 0.05) %>% nrow()
  )
}) %>% do.call(rbind, .)

saveRDS(d.all, 'data/rotenone_sorafenib/deseq_d_all.rds')
saveRDS(results_SR, 'data/rotenone_sorafenib/deseq_DEGs.rds')
rm(ma_genes, samples, counts, genes)
```

The results are saved into an excel file.

```
wb = createWorkbook()
for (n in names(results)) {
  add_data(wb, n, results[[n]][1:500, ])
}
saveWorkbook(wb, 'results/top_tables/top_genes_Rotenone_Sorafenib.xlsx', overwrite = T)
rm(wb)
```

#### **Spline modelling**

In this section, for the 24 hpf exposure data of Sorafenib and
Rotenone, modelling with GAMs is done. In order to do this, the counts
are log(cpm)-transformed using the `limma::voom` method.

```
counts = counts[, samples$LibraryID]
counts = counts[edgeR::filterByExpr(counts), ]

samples_sora = samples[samples$substance != 'Rotenone' & samples$exposure_scenario == 'late', ] %>% 
      dplyr::rename(start_hpf = exposure_start_hpf) %>% 
      add_column(end_hpf = .$time_hpf)
samples_sora = samples[samples$substance != 'Rotenone', ] %>% 
      dplyr::rename(start_hpf = exposure_start_hpf) %>% 
      add_column(end_hpf = .$time_hpf)
voom_sora = voom(
  counts = edgeR::DGEList(
    counts = counts[, samples_sora$LibraryID],
    samples = samples_sora,
    genes = data.frame(ensembl_gene_id = rownames(counts), ProbeName = rownames(counts))
  ),
  normalize.method = 'cyclicloess'
)

samples_rot = samples[samples$substance != 'Sorafenib' & samples$exposure_scenario == 'late', ] %>% 
      dplyr::rename(start_hpf = exposure_start_hpf) %>% 
      add_column(end_hpf = .$time_hpf)
samples_rot = samples[samples$substance != 'Sorafenib', ] %>% 
      dplyr::rename(start_hpf = exposure_start_hpf) %>% 
      add_column(end_hpf = .$time_hpf)
voom_rot = voom(
  counts = edgeR::DGEList(
    counts = counts[, samples_rot$LibraryID],
    samples = samples_rot,
    genes = data.frame(ensembl_gene_id = rownames(counts), ProbeName = rownames(counts))
  ),
  normalize.method = 'cyclicloess'
)

voom_sora_logfc = toxprofileR2::calc_logfc(voom_sora)
voom_rot_logfc = toxprofileR2::calc_logfc(voom_rot)
```

Fitting of the models. Again, as three time points were measured,
*k* is chosen to be three here.

```
fits_sora_gam = fit_gam_batch(voom_sora, voom_sora$genes$ensembl_gene_id, k = 3)
saveRDS(fits_sora_gam, file = 'data/gam/gam_fits_24_sora.rds')

fits_rot_gam = fit_gam_batch(voom_rot, voom_rot$genes$ensembl_gene_id, k = 3)
saveRDS(fits_rot_gam, file = 'data/gam/gam_fits_24_rot.rds')
```

Calculating the simulated differences with confidence intervals.

```
diff_sora_gam = gam_sim_diff_batch(voom_sora, voom_sora$genes$ensembl_gene_id, k = 3)
saveRDS(diff_sora_gam, 'data/gam/gam_diff_24_sora.rds')

diff_rot_gam = gam_sim_diff_batch(voom_rot, voom_rot$genes$ensembl_gene_id, k = 3)
saveRDS(diff_rot_gam, 'data/gam/gam_diff_24_rot.rds')
```

Calculating CI differences.

```
ci_diff_sora = get_sim_CI_diffs(diff_sora_gam, names(diff_sora_gam)) %>% 
  left_join(data_0$genes[, c('ensembl_gene_id', 'external_gene_name')],
            by = c('gene' = 'ensembl_gene_id')) %>% 
  add_column(ensembl_gene_id = .$gene) %>% 
  add_column(exposure = 'late') %>% 
  add_column(substance = 'Sorafenib') %>% 
  add_column(ProbeName = NA)
saveRDS(ci_diff_sora, 'data/gam/gam_ci_diff_24_sora.rds')

ci_diff_rot = get_sim_CI_diffs(diff_rot_gam, names(diff_rot_gam)) %>% 
  left_join(data_0$genes[, c('ensembl_gene_id', 'external_gene_name')],
            by = c('gene' = 'ensembl_gene_id')) %>% 
  add_column(ensembl_gene_id = .$gene) %>% 
  add_column(exposure = 'late') %>% 
  add_column(substance = 'Rotenone') %>% 
  add_column(ProbeName = NA)
saveRDS(ci_diff_rot, 'data/gam/gam_ci_diff_24_rot.rds')
```

Summarising the CI differences to calculate the AUCI95%
values.

```
sig_summ_sora = ci_diff_sora %>% 
  filter(diff != 0) %>% 
  group_by(gene, contrast) %>% 
  summarise(
    auci = sum(diff),
    auci_per_h = sum(diff) / 93,
    duration = sum(diff != 0),
    tmax = t[which.max(abs(diff))],
    direction = diff[which.max(abs(diff))] %>% sign(),
    .groups = 'drop'
  ) %>% 
  left_join(unique(data_0$genes[, c('ensembl_gene_id', 'external_gene_name')]),
            by = c('gene' = 'ensembl_gene_id')) %>% 
  add_column(ensembl_gene_id = .$gene)

sig_summ_rot = ci_diff_rot %>% 
  filter(diff != 0) %>% 
  group_by(gene, contrast) %>% 
  summarise(
    auci = sum(diff),
    auci_per_h = sum(diff) / 93,
    duration = sum(diff != 0),
    tmax = t[which.max(abs(diff))],
    direction = diff[which.max(abs(diff))] %>% sign(),
    .groups = 'drop'
  ) %>% 
  left_join(unique(data_0$genes[, c('ensembl_gene_id', 'external_gene_name')]),
            by = c('gene' = 'ensembl_gene_id')) %>% 
  add_column(ensembl_gene_id = .$gene)

saveRDS(sig_summ_sora, 'data/gam/gam_sig_summ_sora.rds')
saveRDS(sig_summ_rot, 'data/gam/gam_sig_summ_rot.rds')
```

### Overlaps

Venn diagrams of the SU4312 DEG sets are made.

```
venn_data = list(
    early.C1 = sig_summ_0_p %>% filter(contrast == 'C1' & abs(auci_per_h) > c) %>% pull(gene) %>% unique(),
    early.C2 = sig_summ_0_p %>% filter(contrast == 'C2' & abs(auci_per_h) > c) %>% pull(gene) %>% unique(),
    late.C2 = sig_summ_24_p %>% filter(contrast == 'C2' & abs(auci_per_h) > c) %>% pull(gene) %>% unique(),
    late.C3 = sig_summ_24_p %>% filter(contrast == 'C3' & abs(auci_per_h) > c) %>% pull(gene) %>% unique()
  )

# size of text in circles
t_size = 3
# size of set labels
s_size = 3
# scaling of circles to set sizes?
auto = T
# upper y limit for the plots
ymax = 1.4

show_perc = F

# combinations of all conditions
e.C1_e.C2 = ggvenn::ggvenn(
  data = venn_data[c(1, 2)], 
  fill_color = c(conc_cols$C1, conc_cols$C2),
  stroke_size = 0.5,
  show_percentage = show_perc,
  text_size = t_size,
  set_name_size = s_size,
  auto_scale = auto
) + ylim(-1, ymax)
e.C1_l.C2 = ggvenn::ggvenn(
  data = venn_data[c(1, 3)], 
  fill_color = c(conc_cols$C1, conc_cols$C2),
  stroke_size = 0.5,
  show_percentage = show_perc,
  text_size = t_size,
  set_name_size = s_size,
  auto_scale = auto
) + ylim(-1, ymax)
e.C1_l.C3 = ggvenn::ggvenn(
  data = venn_data[c(1, 4)], 
  fill_color = c(conc_cols$C1, conc_cols$C3),
  stroke_size = 0.5,
  show_percentage = show_perc,
  text_size = t_size,
  set_name_size = s_size,
  auto_scale = auto
) + ylim(-1, ymax)
e.C2_l.C2 = ggvenn::ggvenn(
  data = venn_data[c(2, 3)], 
  fill_color = c(conc_cols$C2, conc_cols$C2),
  stroke_size = 0.5,
  show_percentage = show_perc,
  text_size = t_size,
  set_name_size = s_size,
  auto_scale = auto
) + ylim(-1, ymax)
e.C2_l.C3 = ggvenn::ggvenn(
  data = venn_data[c(2, 4)], 
  fill_color = c(conc_cols$C2, conc_cols$C3),
  stroke_size = 0.5,
  show_percentage = show_perc,
  text_size = t_size,
  set_name_size = s_size,
  auto_scale = auto
) + ylim(-1, ymax)
l.C2_l.C3 = ggvenn::ggvenn(
  data = venn_data[c(3, 4)], 
  fill_color = c(conc_cols$C2, conc_cols$C3),
  stroke_size = 0.5,
  show_percentage = show_perc,
  text_size = t_size,
  set_name_size = s_size,
  auto_scale = auto
) + ylim(-1, ymax)

# combining into one plot
space = 0.1
p_2 = cowplot::plot_grid(
  plotlist = list(e.C1_e.C2, e.C2_l.C2,
                  NA, NA,
                  e.C1_l.C2, e.C2_l.C3,
                  NA, NA,
                  e.C1_l.C3, l.C2_l.C3),
  rel_heights = c(1, space, 1, space, 1, 1, space, 1, space, 1),
  ncol = 2,
  scale = 0.95
)

# overlaps of all four conditions
p_venn_4 = ggvenn::ggvenn(
  data = venn_data,
  show_percentage = show_perc,
  fill_color = c(conc_cols$C1, conc_cols$C2, conc_cols$C2, conc_cols$C3),
  stroke_size = 0.5,
  text_size = t_size,
  set_name_size = s_size,
) + xlim(-2.1, 2.1)

p = cowplot::plot_grid(p_venn_4, p_2, rel_widths = c(1.2, 1), scale = 0.9, labels = LETTERS[1:2])

ggsave('deg_venn_4.svg', p_venn_4, 'svg', 'results/figures_final/fig1_venn_global_trans_pattern/', width = 8, height = 8, unit = 'cm', bg = 'white')
ggsave('deg_venn_4_and_pairwise.svg', p, 'svg', 'results/figures_final/fig1_venn_global_trans_pattern/', width = 16, height = 10, unit = 'cm', bg = 'white')
```

## Global analysis

To get a high level overview over the data, the AUCI95%
profiles of all genes are aggregated into a single plot.

The exposure durations are also subdivided into several time frames.
These intervals and their labels are defined here.

```
cuts_SU = data.frame(exp = c(rep('Early exposure', 4), rep('Late exposure', 2)),
                  contrast = c(rep(c('C1', 'C2'), 2), rep(c('C2', 'C3'), 1)),
                  xintercept = rep(c(24, 72, 72), each = 2))

set_names_SU = data.frame(
  name = rep(c('A', 'B', 'C', 'B', 'C'), each = 2),
  x = rep(c(13.5, 48, 85, 48, 85), each = 2),
  y = 675,
  exp = c(rep('Early exposure', 6), rep('Late exposure', 4)),
  contrast = c(rep(c('C1', 'C2'), 3), rep(c('C2', 'C3'), 2)),
  direction = NA
) %>% arrange(exp, contrast, name) %>% 
  add_column(letter = LETTERS[1:nrow(.)])

cuts_SORO = data.frame(exp = c(rep('Late exposure', 1)),
                  contrast = c(rep(c('C1', 'C2'), 1)),
                  xintercept = rep(c(72), 1))

set_names_SORO = data.frame(
  name = rep(c('B', 'C'), 2),
  x = rep(c(48, 85), 2),
  y = 675,
  exp = c(rep('Late exposure', 4)),
  contrast = c(rep(c('C1', 'C2'), each = 2)),
  direction = NA
)

cuts_all = data.frame(
  substance = c(rep('SU4312', 6), rep('Sorafenib', 2), rep('Rotenone', 2)),
  exp = c(rep('Early exposure', 4), rep('Late exposure', 6)),
  contrast = c('C1', 'C1', 'C2', 'C2', 'C2', 'C3', 'C1', 'C2', 'C1', 'C2'),
  xintercept = c(rep(c(24, 72), 2), rep(72, 6))
) %>% 
  mutate(substance = factor(.$substance, levels = c('SU4312', 'Sorafenib', 'Rotenone')))

set_names_all = data.frame(
  substance = c(rep('SU4312', 10), rep('Sorafenib', 4), rep('Rotenone', 4)),
  exp = c(rep('Early exposure', 6), rep('Late exposure', 12)),
  contrast = c(
    rep('C1', 3),
    rep('C2', 3),
    rep('C2', 2),
    rep('C3', 2),
    rep('C1', 2),
    rep('C2', 2),
    rep('C1', 2),
    rep('C2', 2)
  ), 
  x = c(rep(c(13.5, 48, 85), 2), rep(c(48, 85), 6)),
  y = 675,
  name = c(rep(c('A', 'B', 'C'), 2), rep(c('B', 'C'), 6))
) %>% 
  mutate(substance = factor(.$substance, levels = c('SU4312', 'Sorafenib', 'Rotenone'))) %>% 
  add_column(letter = LETTERS[1:nrow(.)])
```

The internal concentration data is loaded and several plots
visualising the data are made.

```
df_internal = read_tsv('data/internal_dose_SUSORO.tsv') %>%
  add_column(mmol_mg = (.$concentration / (.$mol_mass / 1000000))/1000)

# internal dose plot for SU4312
internal_dose_SU = df_internal %>% 
  filter(substance == 'SU4312') %>% 
  ggplot(aes(x = time_hpf, y = concentration)) +
  geom_point() +
  geom_smooth(fill = 'grey80', formula = y ~ x, method = 'loess', colour = 'grey20') +
  facet_wrap(vars(exposure, concentration_level), ncol = 1) +
  # ggtitle('Internal concentrations') +
  scale_x_continuous(breaks = c(0, 24, 48, 72, 96)) +
  xlab('time (hpf)') +
  ylab('concentration (ng/mg)') +
  theme(
    legend.position = 'none',
    panel.grid = element_blank()
  )

# internal dose plot for Rotenone
internal_dose_ROT = df_internal %>% filter(substance == 'Rotenone') %>%
  ggplot(aes(x = time_hpf, y = concentration)) +
  geom_point() +
  geom_smooth(fill = 'grey80', formula = y ~ x, method = 'loess', colour = 'grey20') +
  facet_wrap(vars(exposure, dose), ncol = 1) +
  # ggtitle('Internal dose Rotenone') +
  xlab('time (hpf)') +
  ylab('concentration (ng/egg)') +
  scale_x_continuous(breaks = c(0, 24, 48, 72, 96), limits = c(0, 96)) +
  theme(
    legend.position = 'none',
    panel.grid = element_blank()
  )

# internal dose plot for Sorafenib
internal_dose_SOR = df_internal %>% filter(substance == 'Sorafenib') %>%
  ggplot(aes(x = time_hpf, y = concentration)) +
  geom_point() +
  geom_smooth(fill = 'grey80', formula = y ~ x, method = 'loess', colour = 'grey20') +
  facet_wrap(vars(exposure, dose), ncol = 1) +
  # ggtitle('Internal dose Sorafenib') +
  xlab('time (hpf)') +
  ylab('concentration (ng/egg)') +
  scale_x_continuous(breaks = c(0, 24, 48, 72, 96), limits = c(0, 96)) +
  theme(
    legend.position = 'none',
    panel.grid = element_blank()
  )

# internal dose plot for SU4312, Rotenone, Sorafenib, late exposure, lower concentration
internal_l_C1 = df_internal %>% 
  filter(
    exposure == 'Late exposure' & (substance == 'SU4312' &
                                     concentration_level == 'C2') |
      (substance != 'SU4312' & concentration_level == 'C1')
  ) %>% 
  mutate(substance = factor(substance, levels = c('SU4312', 'Sorafenib', 'Rotenone'))) %>% 
  ggplot(aes(x = time_hpf, y = mmol_mg)) +
  geom_point(size = 0.75) +
  geom_smooth(fill = 'grey80', formula = y ~ x, method = 'loess', colour = 'grey20', linewidth = 0.75) +
  facet_wrap(
    vars(substance, concentration_level),
    ncol = 1,
    labeller = label_wrap_gen(multi_line = F, width = 50),
    scales = 'free_y'
  ) +
  xlab('time (hpf)') +
  ylab('concentration (mmol/mg)') +
  scale_x_continuous(breaks = c(24, 48, 72, 96), limits = c(24, 96)) +
  theme(
    legend.position = 'none',
    panel.grid = element_blank()
  )
# internal dose plot for SU4312, Rotenone, Sorafenib, late exposure, higher concentration
internal_l_C2 = df_internal %>% 
  filter(
    exposure == 'Late exposure' & (substance == 'SU4312' &
                                     concentration_level == 'C3') |
      (substance != 'SU4312' & concentration_level == 'C2')
  ) %>% 
  mutate(substance = factor(substance, levels = c('SU4312', 'Sorafenib', 'Rotenone'))) %>% 
  ggplot(aes(x = time_hpf, y = mmol_mg)) +
  geom_point(size = 0.75) +
  geom_smooth(fill = 'grey80', formula = y ~ x, method = 'loess', colour = 'grey20', linewidth = 0.75) +
  facet_wrap(
    vars(substance, concentration_level),
    ncol = 1,
    labeller = label_wrap_gen(multi_line = F, width = 50),
    scales = 'free_y'
  ) +
  xlab('time (hpf)') +
  ylab('concentration (mmol/mg)') +
  scale_x_continuous(breaks = c(24, 48, 72, 96), limits = c(24, 96)) +
  theme(
    legend.position = 'none',
    panel.grid = element_blank()
  )
```

The plots of the global transcriptional patterns are created.

```
# for SU4312 early and late
p_summ = ci_diff_0_gam %>% 
  add_column(exp = 'Early exposure') %>% 
  rbind(ci_diff_24_gam %>% add_column(exp = 'Late exposure') %>% mutate(t = t + 24)) %>% 
  add_column(direction = sign(.$diff)) %>% 
  group_by(exp, t, contrast, direction) %>% 
  summarise(auci = sum(diff), .groups = 'drop') %>% 
  filter(direction != 0) %>% 
  ggplot(aes(x = t, y = auci, fill = factor(direction), colour = factor(direction))) +
  # geom_vline(xintercept = c(3, 6, 12, 24, 27, 30, 36, 48, 72, 96), colour = 'grey70') +
  geom_area() +
  geom_path() +
  geom_vline(data = cuts_SU, mapping = aes(xintercept = xintercept), colour = 'grey70') +
  geom_text(
    data = set_names_SU,
    mapping = aes(x = x, y = y + 1300, label = letter),
    inherit.aes = F,
    size = 3.5
  ) +
  facet_wrap(vars(exp, contrast), ncol = 1, labeller = label_wrap_gen(multi_line = F), strip.position = 'right') +
  scale_fill_manual(values = c('cornflowerblue', 'firebrick3'), name = 'Direction of regulation', labels = c('down', 'up')) +
  scale_colour_manual(values = c('cornflowerblue', 'firebrick3')) +
  guides(colour = 'none') +
  # ggtitle('Overall AUCI differences over time') +
  scale_x_continuous(breaks = c(0, 24, 48, 72, 96)) +
  scale_y_continuous(breaks = c(-1500, 0, 1500), expand = expansion(add = 1000)) +
  xlab('time (hpf)') +
  ylab(expression(cumulative~AUCI[95*'%'])) +
  t_theme(t_size = 10) +
  theme(
    legend.position = 'none',
    panel.grid = element_blank()
  )
# save for figure in main text
ggsave(
  'SU_global_plot.svg',
  p_summ,
  'svg',
  'results/figures_final/fig1_venn_global_trans_pattern/',
  width = 8,
  height = 11,
  unit = 'cm',
  bg = 'white'
)

# all substances, all conditions
p_summ_all = ci_diff_0_gam %>% 
  add_column(exp = 'Early exposure', substance = 'SU4312') %>% 
  rbind(ci_diff_24_gam %>% add_column(exp = 'Late exposure', substance = 'SU4312') %>% mutate(t = t + 24)) %>% 
  rbind(ci_diff_sora %>% add_column(exp = 'Late exposure', substance = 'Sorafenib') %>% mutate(t = t + 24)) %>% 
  rbind(ci_diff_rot %>% add_column(exp = 'Late exposure', substance = 'Rotenone') %>% mutate(t = t + 24)) %>% 
  add_column(direction = sign(.$diff)) %>% 
  group_by(substance, exp, t, contrast, direction) %>% 
  summarise(auci = sum(diff), .groups = 'drop') %>% 
  filter(direction != 0) %>% 
  mutate(substance = factor(.$substance, levels = c('SU4312', 'Sorafenib', 'Rotenone'))) %>% 
  ggplot(aes(x = t, y = auci, fill = factor(direction), colour = factor(direction))) +
  # geom_vline(xintercept = c(3, 6, 12, 24, 27, 30, 36, 48, 72, 96), colour = 'grey70') +
  geom_area() +
  geom_path() +
  facet_wrap(
    vars(substance, exp, contrast),
    ncol = 4,
    labeller = label_wrap_gen(multi_line = FALSE, width = 40)
  ) +
  geom_vline(data = cuts_all, mapping = aes(xintercept = xintercept), colour = 'grey70') +
  geom_text(data = set_names_all, mapping = aes(x = x, y = y + 1300, label = letter), inherit.aes = F) +
  scale_fill_manual(values = c('cornflowerblue', 'firebrick3'), name = 'Direction of regulation', labels = c('down', 'up')) +
  scale_colour_manual(values = c('cornflowerblue', 'firebrick3')) +
  guides(colour = 'none') +
  # ggtitle('Overall AUCI differences over time') +
  scale_x_continuous(breaks = c(0, 24, 48, 72, 96)) +
  xlab('time (hpf)') +
  ylab('cumulative AUCI over all genes') +
  theme(
    legend.position = 'none',
    panel.grid = element_blank()
  )

# all substances, late, lower concentration
p_summ_all_late_C1 = ci_diff_24_gam %>% 
  filter(contrast == 'C2') %>% 
  add_column(substance = 'SU4312') %>%
  mutate(t = t + 24) %>%
  rbind(ci_diff_sora %>% filter(contrast == 'C1') %>% add_column(substance = 'Sorafenib') %>% mutate(t = t + 24)) %>% 
  rbind(ci_diff_rot %>% filter(contrast == 'C1') %>% add_column(substance = 'Rotenone') %>% mutate(t = t + 24)) %>% 
  add_column(direction = sign(.$diff)) %>% 
  group_by(substance, t, contrast, direction) %>% 
  summarise(auci = sum(diff), .groups = 'drop') %>% 
  filter(direction != 0) %>% 
  mutate(substance = factor(.$substance, levels = c('SU4312', 'Sorafenib', 'Rotenone'))) %>% 
  ggplot(aes(x = t, y = auci, fill = factor(direction), colour = factor(direction))) +
  # geom_vline(xintercept = c(3, 6, 12, 24, 27, 30, 36, 48, 72, 96), colour = 'grey70') +
  geom_area() +
  geom_path() +
  facet_wrap(
    vars(substance, contrast),
    ncol = 1,
    labeller = label_wrap_gen(multi_line = FALSE, width = 40)
  ) +
  geom_vline(
    data = cuts_all %>% filter(exp == 'Late exposure' &
                                 ((substance == 'SU4312' &
                                     contrast == 'C2') |
                                    (substance != 'SU4312' &
                                       contrast == 'C1')
                                 )),
    mapping = aes(xintercept = xintercept),
    colour = 'grey70'
  ) +
  geom_text(
    data = set_names_all %>% filter(exp == 'Late exposure' &
                                      ((substance == 'SU4312' &
                                          contrast == 'C2') |
                                         (substance != 'SU4312' &
                                            contrast == 'C1')
                                      )),
    mapping = aes(x = x, y = y + 1300, label = letter),
    inherit.aes = F,
    size = 9 / .pt,
    family = 'Arial'
  )+
  scale_fill_manual(values = c('cornflowerblue', 'firebrick3'), name = 'Direction of regulation', labels = c('down', 'up')) +
  scale_colour_manual(values = c('cornflowerblue', 'firebrick3')) +
  guides(colour = 'none') +
  # ggtitle('Overall AUCI differences over time') +
  scale_x_continuous(breaks = c(0, 24, 48, 72, 96)) +
  scale_y_continuous(limits = c(-2700, 2500)) + 
  xlab('time (hpf)') +
  ylab(expression(cumulative~AUCI[95*'%'])) +
  theme(
    legend.position = 'none',
    panel.grid = element_blank()
  )
# all substances, late, higher concentration
p_summ_all_late_C2 = ci_diff_24_gam %>% 
  filter(contrast == 'C3') %>% 
  add_column(substance = 'SU4312') %>%
  mutate(t = t + 24) %>%
  rbind(ci_diff_sora %>% filter(contrast == 'C2') %>% add_column(substance = 'Sorafenib') %>% mutate(t = t + 24)) %>% 
  rbind(ci_diff_rot %>% filter(contrast == 'C2') %>% add_column(substance = 'Rotenone') %>% mutate(t = t + 24)) %>% 
  add_column(direction = sign(.$diff)) %>% 
  group_by(substance, t, contrast, direction) %>% 
  summarise(auci = sum(diff), .groups = 'drop') %>% 
  filter(direction != 0) %>% 
  mutate(substance = factor(.$substance, levels = c('SU4312', 'Sorafenib', 'Rotenone'))) %>% 
  ggplot(aes(x = t, y = auci, fill = factor(direction), colour = factor(direction))) +
  # geom_vline(xintercept = c(3, 6, 12, 24, 27, 30, 36, 48, 72, 96), colour = 'grey70') +
  geom_area() +
  geom_path() +
  facet_wrap(
    vars(substance, contrast),
    ncol = 1,
    labeller = label_wrap_gen(multi_line = FALSE, width = 40)
  ) +
  geom_vline(
    data = cuts_all %>% filter(exp == 'Late exposure' &
                                 ((substance == 'SU4312' &
                                     contrast == 'C3') |
                                    (substance != 'SU4312' &
                                       contrast == 'C2')
                                 )),
    mapping = aes(xintercept = xintercept),
    colour = 'grey70'
  )+
  geom_text(
    data = set_names_all %>% filter(exp == 'Late exposure' &
                                      ((substance == 'SU4312' &
                                          contrast == 'C3') |
                                         (substance != 'SU4312' &
                                            contrast == 'C2')
                                      )),
    mapping = aes(x = x, y = y + 1300, label = letter),
    inherit.aes = F,
    size = 9 / .pt,
    family = 'Arial'
  )+
  scale_fill_manual(values = c('cornflowerblue', 'firebrick3'), name = 'Direction of regulation', labels = c('down', 'up')) +
  scale_colour_manual(values = c('cornflowerblue', 'firebrick3')) +
  guides(colour = 'none') +
  # ggtitle('Overall AUCI differences over time') +
  scale_x_continuous(breaks = c(0, 24, 48, 72, 96)) +
  scale_y_continuous(limits = c(-2700, 2500)) + 
  xlab('time (hpf)') +
  ylab(expression(cumulative~AUCI[95*'%'])) +
  theme(
    legend.position = 'none',
    panel.grid = element_blank()
  )

p_summ_all
```

A plot contrasting the late exposure global transcriptional patterns
with the respective internal doses, additionally the time course
expression for kdrl is plotted. This figure is shown in the main text.
Additionally, a plot visualizing the kdrl time series expression,
including the SU4312 early exposure is shown.

```
t = theme(
    axis.text = element_text(family = 'Arial', size = 7),
    legend.text = element_text(family = 'Arial', size = 10),
    legend.position = 'none',
    axis.title = element_text(family = 'Arial', size = 8),
    strip.text = element_text(family = 'Arial', size = 8),
    axis.ticks = element_line(linewidth = 0.5),
    axis.ticks.length = unit(0.6, 'mm')
    # aspect.ratio = 1.3
  )

g = 'ENSDARG00000105215' # kdrl

data_kdrl = list(
  plot_gam(g, data_24, c('C2', 'C3'),  time = 'time_hpf', start_hpf = 24, return_data = T),
  plot_gam(g, voom_sora, c('C1', 'C2'),  time = 'time_hpf', start_hpf = 24, return_data = T),
  plot_gam(g, voom_rot, c('C1', 'C2'),  time = 'time_hpf', start_hpf = 24, return_data = T)
)

data_measured_kdrl = rbind(
  data_kdrl[[1]][[1]] %>% add_column(substance = 'SU4312'),
  data_kdrl[[2]][[1]] %>% add_column(substance = 'Sorafenib'),
  data_kdrl[[3]][[1]] %>% add_column(substance = 'Rotenone')
) %>% 
  mutate(substance = factor(.$substance, levels = c('SU4312', 'Sorafenib', 'Rotenone')))
data_fit_kdrl = rbind(
  data_kdrl[[1]][[2]] %>% add_column(substance = 'SU4312'),
  data_kdrl[[2]][[2]] %>% add_column(substance = 'Sorafenib'),
  data_kdrl[[3]][[2]] %>% add_column(substance = 'Rotenone')
) %>% 
  mutate(substance = factor(.$substance, levels = c('SU4312', 'Sorafenib', 'Rotenone')))


p_kdrl = data_measured_kdrl %>%
  ggplot(aes(
    x = jitter(t, 1.1),
    y = E,
    colour = concentration_level
  )) +
  geom_path(data = data_fit_kdrl,
            mapping = aes(x = t, y = p, colour = concentration_level)) +
  geom_ribbon(
    data = data_fit_kdrl,
    mapping = aes(
      x = t,
      ymin = p_lower,
      ymax = p_upper,
      colour = concentration_level
    ),
    inherit.aes = F,
    fill = alpha('black', 0.1),
    linetype = 1,
    linewidth = 0.1
  ) +
  geom_point(shape = 20, size = 1) +
  facet_wrap(.~substance, ncol = 1, scales = 'free_y') +
  scale_colour_manual(name = 'concentration level', values = conc_cols_vector) +
  scale_x_continuous(breaks = c(24, 48, 72, 96), limits = c(24, 100)) +
  scale_y_continuous(expand = expansion(mult = 0.5)) +
  xlab('time (hpf)') +
  ylab('log(Expression)') +
  t_theme()

p = cowplot::plot_grid(
  p_summ_all_late_C1 + t,
  internal_l_C1 + t,
  p_summ_all_late_C2 + t,
  internal_l_C2 + t,
  p_kdrl + t, 
  ncol = 5,
  align = 'hv'
)

ggsave('results/figures_final/internal_dose_trans_pattern_kdrl/internal_trans_pattern_kdrl.svg', plot = p, 'svg', width = 19.5, height = 12, units = 'cm', bg = 'white')

legend = ggpubr::get_legend(ggplot(data.frame(lvl = c('C0', 'C1', 'C2', 'C3'), x = 1:8, y = 1:8), aes(x = x, y = y, colour = lvl)) + geom_point() + geom_path() + scale_colour_manual(name = 'Concentration level', values = c(C0 = 'cornflowerblue', C1 = 'gold1', C2 = 'orangered2', C3 = 'darkred')) + t_theme(t_size = 10)) %>% ggpubr::as_ggplot()
svg(
  filename = 'results/figures_final/internal_dose_trans_pattern_kdrl/kdrl_legend.svg',
  width = 5, height = 2
)
legend
dev.off()
```

```
## png 
##   2
```

```
# a plot for kdrl including the early SU4312 exposure
p = cowplot::plot_grid(
  cowplot::plot_grid(
      plot_gam(g, data_0, c('C1', 'C2'), time = 'time_hpf', start_hpf = 0) +
        scale_x_continuous(breaks = c(0, 24, 48, 72, 96), limits = c(0, 97)) +
        scale_y_continuous(breaks = c(3, 6, 9), limits = c(2, 10)) +
        ggtitle('SU4312') +
        theme(legend.position = 'none'),
      plot_gam(g, data_24, c('C2', 'C3'),  time = 'time_hpf', start_hpf = 24) +
        scale_x_continuous(breaks = c(0, 24, 48, 72, 96), limits = c(0, 97)) +
        scale_y_continuous(breaks = c(7.5, 8.5, 9.5), limits = c(7, 10)) +
        ggtitle('SU4312') +
        theme(legend.position = 'none'),
      plot_gam(g, voom_sora, c('C1', 'C2'),  time = 'time_hpf', start_hpf = 24,
               col_scale = c(C0 = 'cornflowerblue', C1 = 'gold1', C2 = 'darkred')) +
        scale_x_continuous(breaks = c(0, 24, 48, 72, 96), limits = c(0, 97)) +
        scale_y_continuous(breaks = c(3.5, 4.5, 5.5), limits = c(3, 6)) +
        ggtitle('Sorafenib') +
        theme(legend.position = 'none'),
      plot_gam(g, voom_rot, c('C1', 'C2'),  time = 'time_hpf', start_hpf = 24,
               col_scale = c(C0 = 'cornflowerblue', C1 = 'gold1', C2 = 'darkred')) +
        scale_x_continuous(breaks = c(0, 24, 48, 72, 96), limits = c(0, 97)) +
        scale_y_continuous(breaks = c(4.5, 5.5), limits = c(4, 6)) +
        ggtitle('Rotenone') +
        theme(legend.position = 'none'),
      ncol = 2
    ),
  legend,
  ncol = 1,
  rel_heights = c(1, 0.1)
)
p
```

A plot contrasting the global transcriptional patterns of SU4312
against the internal doses over time for both exposure scenarios.

```
# summ vs internal dose
cowplot::plot_grid(
  p_summ,
  internal_dose_SU + geom_vline(
    data = cuts_SU,
    mapping = aes(xintercept = xintercept),
    colour = 'grey70'
  ),
  ncol = 2
)
```

## Enrichments

Enrichment terms are loaded. Here, genes are matched with assigned
term IDs and term IDs with descriptions/ names, as it is used in
`clusterprofileR`.

```
enrichment_terms = readRDS('data/enrichment_terms.rds')
```

### Over-representation analysis

#### SU4312

Gene sets of all genes that are differentially expressed in the
respective time periods. These sets have overlaps, as genes can be
differentially expressed over long periods of time.

```
gene_sets_SU = list()

gene_sets_SU$SU4312.e.A.C1 = ci_diff_0_gam %>% filter(contrast == 'C1' & t <= 24) %>% 
  group_by(gene, ensembl_gene_id, external_gene_name) %>%
  summarise(sum_ci = sum(diff), ci_per_h = sum(diff)/n(), .groups = 'drop') %>%
  filter(abs(ci_per_h) > c) %>% add_column(direction = sign(.$sum_ci))
gene_sets_SU$SU4312.e.D.C2 = ci_diff_0_gam %>% filter(contrast == 'C2' & t <= 24) %>% 
  group_by(gene, ensembl_gene_id, external_gene_name) %>%
  summarise(sum_ci = sum(diff), ci_per_h = sum(diff)/n(), .groups = 'drop') %>%
  filter(abs(ci_per_h) > c) %>% add_column(direction = sign(.$sum_ci))
gene_sets_SU$SU4312.e.AD.int = rbind(
  gene_sets_SU$SU4312.e.A.C1 %>% filter(gene %in% intersect(gene_sets_SU$SU4312.e.A.C1$gene, gene_sets_SU$SU4312.e.D.C2$gene)),
  gene_sets_SU$SU4312.e.D.C2 %>% filter(gene %in% intersect(gene_sets_SU$SU4312.e.A.C1$gene, gene_sets_SU$SU4312.e.D.C2$gene))
)

gene_sets_SU$SU4312.e.B.C1 = ci_diff_0_gam %>% filter(contrast == 'C1' & t > 24 & t <= 72) %>% 
  group_by(gene, ensembl_gene_id, external_gene_name) %>%
  summarise(sum_ci = sum(diff), ci_per_h = sum(diff)/n(), .groups = 'drop') %>%
  filter(abs(ci_per_h) > c) %>% add_column(direction = sign(.$sum_ci))
gene_sets_SU$SU4312.e.E.C2 = ci_diff_0_gam %>% filter(contrast == 'C2' & t > 24 & t <= 72) %>% 
  group_by(gene, ensembl_gene_id, external_gene_name) %>%
  summarise(sum_ci = sum(diff), ci_per_h = sum(diff)/n(), .groups = 'drop') %>%
  filter(abs(ci_per_h) > c) %>% add_column(direction = sign(.$sum_ci))
gene_sets_SU$SU4312.e.BE.int = rbind(
  gene_sets_SU$SU4312.e.B.C1 %>% filter(gene %in% intersect(gene_sets_SU$SU4312.e.B.C1$gene, gene_sets_SU$SU4312.e.E.C2$gene)),
  gene_sets_SU$SU4312.e.E.C2 %>% filter(gene %in% intersect(gene_sets_SU$SU4312.e.B.C1$gene, gene_sets_SU$SU4312.e.E.C2$gene))
)

gene_sets_SU$SU4312.e.C.C1 = ci_diff_0_gam %>% filter(contrast == 'C1' & t > 72) %>% 
  group_by(gene, ensembl_gene_id, external_gene_name) %>%
  summarise(sum_ci = sum(diff), ci_per_h = sum(diff)/n(), .groups = 'drop') %>%
  filter(abs(ci_per_h) > c) %>% add_column(direction = sign(.$sum_ci))
gene_sets_SU$SU4312.e.F.C2 = ci_diff_0_gam %>% filter(contrast == 'C2' & t > 72) %>% 
  group_by(gene, ensembl_gene_id, external_gene_name) %>%
  summarise(sum_ci = sum(diff), ci_per_h = sum(diff)/n(), .groups = 'drop') %>%
  filter(abs(ci_per_h) > c) %>% add_column(direction = sign(.$sum_ci))
gene_sets_SU$SU4312.e.CF.int = rbind(
  gene_sets_SU$SU4312.e.C.C1 %>% filter(gene %in% intersect(gene_sets_SU$SU4312.e.C.C1$gene, gene_sets_SU$SU4312.e.F.C2$gene)),
  gene_sets_SU$SU4312.e.F.C2 %>% filter(gene %in% intersect(gene_sets_SU$SU4312.e.C.C1$gene, gene_sets_SU$SU4312.e.F.C2$gene))
)


gene_sets_SU$SU4312.l.G.C2 = ci_diff_24_gam %>% filter(contrast == 'C2' & t <= 72-24) %>% 
  group_by(gene, ensembl_gene_id, external_gene_name) %>%
  summarise(sum_ci = sum(diff), ci_per_h = sum(diff)/n(), .groups = 'drop') %>%
  filter(abs(ci_per_h) > c) %>% add_column(direction = sign(.$sum_ci))
gene_sets_SU$SU4312.l.I.C3 = ci_diff_24_gam %>% filter(contrast == 'C3' & t <= 72-24) %>% 
  group_by(gene, ensembl_gene_id, external_gene_name) %>%
  summarise(sum_ci = sum(diff), ci_per_h = sum(diff)/n(), .groups = 'drop') %>%
  filter(abs(ci_per_h) > c) %>% add_column(direction = sign(.$sum_ci))
gene_sets_SU$SU4312.l.GI.int = rbind(
  gene_sets_SU$SU4312.l.G.C2 %>% filter(gene %in% intersect(gene_sets_SU$SU4312.l.G.C2$gene, gene_sets_SU$SU4312.l.I.C3$gene)),
  gene_sets_SU$SU4312.l.I.C3 %>% filter(gene %in% intersect(gene_sets_SU$SU4312.l.G.C2$gene, gene_sets_SU$SU4312.l.I.C3$gene))
)

gene_sets_SU$SU4312.l.H.C2 = ci_diff_24_gam %>% filter(contrast == 'C2' & t > 72-24) %>% 
  group_by(gene, ensembl_gene_id, external_gene_name) %>%
  summarise(sum_ci = sum(diff), ci_per_h = sum(diff)/n(), .groups = 'drop') %>%
  filter(abs(ci_per_h) > c) %>% add_column(direction = sign(.$sum_ci))
gene_sets_SU$SU4312.l.J.C3 = ci_diff_24_gam %>% filter(contrast == 'C3' & t > 72-24) %>% 
  group_by(gene, ensembl_gene_id, external_gene_name) %>%
  summarise(sum_ci = sum(diff), ci_per_h = sum(diff)/n(), .groups = 'drop') %>%
  filter(abs(ci_per_h) > c) %>% add_column(direction = sign(.$sum_ci))
gene_sets_SU$SU4312.l.HJ.int = rbind(
  gene_sets_SU$SU4312.l.H.C2 %>% filter(gene %in% intersect(gene_sets_SU$SU4312.l.H.C2$gene, gene_sets_SU$SU4312.l.J.C3$gene)),
  gene_sets_SU$SU4312.l.J.C3 %>% filter(gene %in% intersect(gene_sets_SU$SU4312.l.H.C2$gene, gene_sets_SU$SU4312.l.J.C3$gene))
)

gene_sets_SORO = list()

gene_sets_SORO$Sora.l.K.C1 = ci_diff_sora %>% filter(contrast == 'C1' & t <= 72-24) %>% 
  group_by(gene, ensembl_gene_id, external_gene_name) %>%
  summarise(sum_ci = sum(diff), ci_per_h = sum(diff)/n(), .groups = 'drop') %>%
  filter(abs(ci_per_h) > c) %>% add_column(direction = sign(.$sum_ci))
gene_sets_SORO$Sora.l.M.C2 = ci_diff_sora %>% filter(contrast == 'C2' & t <= 72-24) %>% 
  group_by(gene, ensembl_gene_id, external_gene_name) %>%
  summarise(sum_ci = sum(diff), ci_per_h = sum(diff)/n(), .groups = 'drop') %>%
  filter(abs(ci_per_h) > c) %>% add_column(direction = sign(.$sum_ci))
gene_sets_SORO$Sora.l.KM.int = rbind(
  gene_sets_SORO$Sora.l.K.C1 %>% filter(gene %in% intersect(gene_sets_SORO$Sora.l.K.C1$gene, gene_sets_SORO$Sora.l.M.C2$gene)),
  gene_sets_SORO$Sora.l.M.C2 %>% filter(gene %in% intersect(gene_sets_SORO$Sora.l.K.C1$gene, gene_sets_SORO$Sora.l.M.C2$gene))
)

gene_sets_SORO$Sora.l.L.C1 = ci_diff_sora %>% filter(contrast == 'C1' & t > 72-24) %>% 
  group_by(gene, ensembl_gene_id, external_gene_name) %>%
  summarise(sum_ci = sum(diff), ci_per_h = sum(diff)/n(), .groups = 'drop') %>%
  filter(abs(ci_per_h) > c) %>% add_column(direction = sign(.$sum_ci))
gene_sets_SORO$Sora.l.N.C2 = ci_diff_sora %>% filter(contrast == 'C2' & t > 72-24) %>% 
  group_by(gene, ensembl_gene_id, external_gene_name) %>%
  summarise(sum_ci = sum(diff), ci_per_h = sum(diff)/n(), .groups = 'drop') %>%
  filter(abs(ci_per_h) > c) %>% add_column(direction = sign(.$sum_ci))
gene_sets_SORO$Sora.l.LN.int = rbind(
  gene_sets_SORO$Sora.l.L.C1 %>% filter(gene %in% intersect(gene_sets_SORO$Sora.l.L.C1$gene, gene_sets_SORO$Sora.l.N.C2$gene)),
  gene_sets_SORO$Sora.l.N.C2 %>% filter(gene %in% intersect(gene_sets_SORO$Sora.l.L.C1$gene, gene_sets_SORO$Sora.l.N.C2$gene))
)

gene_sets_SORO$Rote.l.O.C1 = ci_diff_rot %>% filter(contrast == 'C1' & t <= 72-24) %>% 
  group_by(gene, ensembl_gene_id, external_gene_name) %>%
  summarise(sum_ci = sum(diff), ci_per_h = sum(diff)/n(), .groups = 'drop') %>%
  filter(abs(ci_per_h) > c) %>% add_column(direction = sign(.$sum_ci))
gene_sets_SORO$Rote.l.Q.C2 = ci_diff_rot %>% filter(contrast == 'C2' & t <= 72-24) %>% 
  group_by(gene, ensembl_gene_id, external_gene_name) %>%
  summarise(sum_ci = sum(diff), ci_per_h = sum(diff)/n(), .groups = 'drop') %>%
  filter(abs(ci_per_h) > c) %>% add_column(direction = sign(.$sum_ci))
gene_sets_SORO$Rote.l.OQ.int = rbind(
  gene_sets_SORO$Rote.l.O.C1 %>% filter(gene %in% intersect(gene_sets_SORO$Rote.l.O.C1$gene, gene_sets_SORO$Rote.l.Q.C2$gene)),
  gene_sets_SORO$Rote.l.Q.C2 %>% filter(gene %in% intersect(gene_sets_SORO$Rote.l.O.C1$gene, gene_sets_SORO$Rote.l.Q.C2$gene))
)

gene_sets_SORO$Rote.l.P.C1 = ci_diff_rot %>% filter(contrast == 'C1' & t > 72-24) %>% 
  group_by(gene, ensembl_gene_id, external_gene_name) %>%
  summarise(sum_ci = sum(diff), ci_per_h = sum(diff)/n(), .groups = 'drop') %>%
  filter(abs(ci_per_h) > c) %>% add_column(direction = sign(.$sum_ci))
gene_sets_SORO$Rote.l.R.C2 = ci_diff_rot %>% filter(contrast == 'C2' & t > 72-24) %>% 
  group_by(gene, ensembl_gene_id, external_gene_name) %>%
  summarise(sum_ci = sum(diff), ci_per_h = sum(diff)/n(), .groups = 'drop') %>%
  filter(abs(ci_per_h) > c) %>% add_column(direction = sign(.$sum_ci))
gene_sets_SORO$Rote.l.PR.int = rbind(
  gene_sets_SORO$Rote.l.P.C1 %>% filter(gene %in% intersect(gene_sets_SORO$Rote.l.P.C1$gene, gene_sets_SORO$Rote.l.R.C2$gene)),
  gene_sets_SORO$Rote.l.R.C2 %>% filter(gene %in% intersect(gene_sets_SORO$Rote.l.P.C1$gene, gene_sets_SORO$Rote.l.R.C2$gene))
)

# gene sets without threshold
gene_sets_SU_all = list()

gene_sets_SU_all$SU4312.e.A.C1 = ci_diff_0_gam %>% filter(contrast == 'C1' & t <= 24) %>% 
  group_by(gene, ensembl_gene_id, external_gene_name) %>%
  summarise(sum_ci = sum(diff), ci_per_h = sum(diff)/n(), .groups = 'drop')
gene_sets_SU_all$SU4312.e.A.C2 = ci_diff_0_gam %>% filter(contrast == 'C2' & t <= 24) %>% 
  group_by(gene, ensembl_gene_id, external_gene_name) %>%
  summarise(sum_ci = sum(diff), ci_per_h = sum(diff)/n(), .groups = 'drop')

gene_sets_SU_all$SU4312.e.B.C1 = ci_diff_0_gam %>% filter(contrast == 'C1' & t > 24 & t <= 72) %>% 
  group_by(gene, ensembl_gene_id, external_gene_name) %>%
  summarise(sum_ci = sum(diff), ci_per_h = sum(diff)/n(), .groups = 'drop')
gene_sets_SU_all$SU4312.e.B.C2 = ci_diff_0_gam %>% filter(contrast == 'C2' & t > 24 & t <= 72) %>% 
  group_by(gene, ensembl_gene_id, external_gene_name) %>%
  summarise(sum_ci = sum(diff), ci_per_h = sum(diff)/n(), .groups = 'drop')

gene_sets_SU_all$SU4312.e.C.C1 = ci_diff_0_gam %>% filter(contrast == 'C1' & t > 72) %>% 
  group_by(gene, ensembl_gene_id, external_gene_name) %>%
  summarise(sum_ci = sum(diff), ci_per_h = sum(diff)/n(), .groups = 'drop')
gene_sets_SU_all$SU4312.e.C.C2 = ci_diff_0_gam %>% filter(contrast == 'C2' & t > 72) %>% 
  group_by(gene, ensembl_gene_id, external_gene_name) %>%
  summarise(sum_ci = sum(diff), ci_per_h = sum(diff)/n(), .groups = 'drop')

gene_sets_SU_all$SU4312.l.B.C2 = ci_diff_24_gam %>% filter(contrast == 'C2' & t <= 72-24) %>% 
  group_by(gene, ensembl_gene_id, external_gene_name) %>%
  summarise(sum_ci = sum(diff), ci_per_h = sum(diff)/n(), .groups = 'drop')
gene_sets_SU_all$SU4312.l.B.C3 = ci_diff_24_gam %>% filter(contrast == 'C3' & t <= 72-24) %>% 
  group_by(gene, ensembl_gene_id, external_gene_name) %>%
  summarise(sum_ci = sum(diff), ci_per_h = sum(diff)/n(), .groups = 'drop')

gene_sets_SU_all$SU4312.l.C.C2 = ci_diff_24_gam %>% filter(contrast == 'C2' & t > 72-24) %>% 
  group_by(gene, ensembl_gene_id, external_gene_name) %>%
  summarise(sum_ci = sum(diff), ci_per_h = sum(diff)/n(), .groups = 'drop')
gene_sets_SU_all$SU4312.l.C.C3 = ci_diff_24_gam %>% filter(contrast == 'C3' & t > 72-24) %>% 
  group_by(gene, ensembl_gene_id, external_gene_name) %>%
  summarise(sum_ci = sum(diff), ci_per_h = sum(diff)/n(), .groups = 'drop')
```

```
wb = createWorkbook()
for (n in names(gene_sets_SU)) {
  add_data(wb, n, gene_sets_SU[[n]])
}
saveWorkbook(wb, 'results/top_tables/SU4312_DEGs.xlsx', overwrite = T)

wb = createWorkbook()
for (n in names(gene_sets_SORO)) {
  add_data(wb, n, gene_sets_SORO[[n]])
}
saveWorkbook(wb, 'results/top_tables/SORO_DEGs.xlsx', overwrite = T)
```

For the SU4312 DEG sets, a similarity matrix is calculated using the
overlap coefficient. This plot is shown in the main text.

```
SU_DEGs_matrix = gene_sets_SU[!str_detect(names(gene_sets_SU), 'int')]

overlap_coef = function(x, y) {
  length(intersect(x, y)) / min(c(length(x), length(y)))
}

matrix_to_df = function(matrix) {
  # matrix[lower.tri(matrix)] <- NA
  # diag(matrix) <- NA
  out <- na.omit(data.frame(as.table(matrix)))
  out[order(out$Var1), ]
}

df_overlap_coef = sapply(SU_DEGs_matrix, function(x) {
  sapply(SU_DEGs_matrix, function(y) {
    overlap_coef(x$ensembl_gene_id, y$ensembl_gene_id)
  })
}) %>%
  matrix_to_df() %>% 
  dplyr::rename(overlap_coef = Freq)
df_intersection = sapply(SU_DEGs_matrix, function(x) {
  sapply(SU_DEGs_matrix, function(y) {
    length(intersect(x$ensembl_gene_id, y$ensembl_gene_id))
  })
}) %>%
  matrix_to_df() %>% 
  dplyr::rename(intersection = Freq)

df_matrix = full_join(df_overlap_coef, df_intersection, by = c('Var1', 'Var2'))
df_matrix$is_diag = df_matrix$Var1 == df_matrix$Var2

p = df_matrix %>% ggplot(aes(
  x = Var1,
  y = Var2,
  fill = overlap_coef,
  label = intersection
)) +
  geom_tile() +
  geom_text(mapping = aes(colour = is_diag), size = 9/.pt, family = 'Arial') +
  geom_rect(
    data = data.frame(xmin = c(0.5, 6.5), xmax = c(6.5, 10.5), ymin = c(0.5, 6.5), ymax = c(6.5, 10.5)),
    mapping = aes(xmin = xmin, xmax = xmax, ymin = ymin, ymax = ymax),
    inherit.aes = F,
    fill = alpha('white', 0),
    colour = 'black',
    linewidth = 0.6,
    family = 'Arial'
  ) +
  scale_colour_manual(values = c('grey20', 'grey70'), guide = 'none') +
  scale_fill_distiller(
    palette = 'BuPu',
    values = c(0, .1, .2, .3, .4, .5, 1),
    breaks = c(0.2, 0.5, 0.8),
    direction = 1,
    name = 'Set similarity (Overlap coefficient)'
  ) +
  coord_fixed() +
  theme(
    axis.text.x = element_text(angle = 45, hjust = 1),
    axis.text.y = element_text(angle = 45, hjust = 1),
    axis.title = element_blank(),
    legend.text = element_text(size = 10, family = 'Arial')
  ) +
  theme(panel.border = element_blank())

ggsave(
  'similarity_matrix.svg',
  p,
  'svg',
  'results/figures_final/fig2_similarity_matrix/',
  width = 15,
  height = 16,
  unit = 'cm',
  bg = 'white'
)
```

A wrapper for the enrichment function is defined. This performs an
ORA of a set of genes in the databases defined in
`enrichment_terms` using `enrich_all`. Then,
additional columns are created that summarise AUCI95% values
of all genes in a gene set.

```
enrich_wrapper = function(gene_df, enrichment_terms, return_df = T, combine_result = T, universe = NULL) {
  enrichment_combined = enrich_all(gene_df$ensembl_gene_id, enrichment_terms, return_df = T, combine_result = T, universe = universe)
  
  enrichment_combined$mean_ci_per_h = lapply(enrichment_combined$geneID, function(genes) {
    genes = str_split(genes, '/') %>% unlist()
    gene_df[gene_df$ensembl_gene_id %in% genes, ] %>% dplyr::select(contains('ci_per_h')) %>% as.matrix() %>% mean(na.rm = T)
  }) %>% as.numeric()
  enrichment_combined$median_ci_per_h = lapply(enrichment_combined$geneID, function(genes) {
    genes = str_split(genes, '/') %>% unlist()
    gene_df[gene_df$ensembl_gene_id %in% genes, ] %>% dplyr::select(contains('ci_per_h')) %>% as.matrix() %>% median(na.rm = T)
  }) %>% as.numeric()
  enrichment_combined$sd_ci_per_h = lapply(enrichment_combined$geneID, function(genes) {
    genes = str_split(genes, '/') %>% unlist()
    gene_df[gene_df$ensembl_gene_id %in% genes, ] %>% dplyr::select(contains('ci_per_h')) %>% as.matrix() %>% sd(na.rm = T)
  }) %>% as.numeric()
  
  return(enrichment_combined)
}
```

The ORA is performed for the SU4312 data.

```
enr.SU4312.ORA = pbapply::pblapply(names(gene_sets_SU), function(name) {
  enrich_wrapper(gene_sets_SU[[name]], enrichment_terms)
}) %>% set_names(names(gene_sets_SU))

saveRDS(enr.SU4312.ORA, 'data/enrichments_SU4312/enrichments_SU4312_ORA.rds')
```

Saving the enrichment into an excel file

```
wb = createWorkbook()
for (n in names(enr.SU4312.ORA)) {
  add_data(wb, n, enr.SU4312.ORA[[n]])
}
saveWorkbook(wb, 'results/enrichments/enrichments_ORA_SU4312.xlsx', overwrite = T)
```

#### Sorafenib & Rotenone

ORA for the DESeq analysis.

```
DEGs = lapply(names(results_SR), function(n) {
  results_SR[[n]] %>% filter(padj < 0.05) %>% pull(ensembl_gene_id)
}) %>% set_names(names(results_SR))

enr.SR.ORA = lapply(names(DEGs), function(n) {
  ens = DEGs[[n]]
  if (length(ens) == 0) {return(NA)}
  
  enrich_all(ens, enrichment_terms)
}) %>% set_names(names(DEGs))

saveRDS(enr.SR.ORA, 'data/rotenone_sorafenib/enr_ORA_sora_rot.rds')
```

ORA for the Sorafenib and Rotenone DEGs from the GAM models.

```
enr.SR.ORA.gam = pbapply::pblapply(names(gene_sets_SORO), function(name) {
  enrich_wrapper(gene_sets_SORO[[name]], enrichment_terms)
}) %>% set_names(names(gene_sets_SORO))
enr.SR.ORA.gam$Sora.e.96.C2 = enr.SR.ORA$Sorafenib.e.96.C2
enr.SR.ORA.gam$Rote.e.96.C1 = enr.SR.ORA$Rotenone.e.96.C1

saveRDS(enr.SR.ORA.gam, 'data/rotenone_sorafenib/enrichments_SR_ORA_gam.rds')
```

Writing to excel files.

```
wb = createWorkbook()
for (n in names(enr.SR.ORA)) {
  if (is.data.frame(enr.SR.ORA[[n]])) {
    add_data(wb, n, enr.SR.ORA[[n]][1:1000, ])
  } else {
    add_data(wb, n, data.frame())
  }
}
saveWorkbook(wb, 'results/enrichments/enrichments_ORA_Rotenone_Sorafenib.xlsx', overwrite = T)
rm(wb)

wb = createWorkbook()
for (n in names(enr.SR.ORA.gam)) {
  if (is.data.frame(enr.SR.ORA.gam[[n]])) {
    add_data(wb, n, enr.SR.ORA.gam[[n]][1:1000, ])
  } else {
    add_data(wb, n, data.frame())
  }
}
saveWorkbook(wb, 'results/enrichments/enrichments_ORA_gam_Rotenone_Sorafenib.xlsx', overwrite = T)
rm(wb)
```

#### Intersection

Venn diagrams of all genes DE in any condition (substance, time frame
or concentration) and all genes DE in any late exposure condition are
made.

```
all_genes = list(
    SU4312 = lapply(gene_sets_SU, function(x) {
      x$gene
    }) %>% do.call('c', .),
    Sorafenib = c(
      lapply(gene_sets_SORO[1:6], function(x) {
        x$gene
      }) %>% do.call('c', .),
      results_SR$Sorafenib.e.96.C2 %>% filter(padj < 0.05) %>% pull(ensembl_gene_id)
    ),
    Rotenone = c(
      lapply(gene_sets_SORO[7:12], function(x) {
        x$gene
      }) %>% do.call('c', .),
      results_SR$Rotenone.e.96.C1 %>% filter(padj < 0.05) %>% pull(ensembl_gene_id)
    )
  )

all_genes_vector = do.call('c', all_genes) %>% unique()

df_all = data.frame(
  SU4312 = all_genes_vector %in% all_genes$SU4312,
  Sorafenib = all_genes_vector %in% all_genes$Sorafenib,
  Rotenone = all_genes_vector %in% all_genes$Rotenone
)

all_genes_late = list(
    SU4312 = lapply(gene_sets_SU[10:15], function(x) {
      x$gene
    }) %>% do.call('c', .),
    Sorafenib =
      lapply(gene_sets_SORO[1:6], function(x) {
        x$gene
      }) %>% do.call('c', .),
    Rotenone =
      lapply(gene_sets_SORO[7:12], function(x) {
        x$gene
      }) %>% do.call('c', .)
  )

all_genes_late_vector = do.call('c', all_genes_late) %>% unique()
df_late = data.frame(
  SU4312 = all_genes_late_vector %in% all_genes_late$SU4312,
  Sorafenib = all_genes_late_vector %in% all_genes_late$Sorafenib,
  Rotenone = all_genes_late_vector %in% all_genes_late$Rotenone
)


euler(df_all) %>% plot(quantities = T)
```

```
euler(df_late) %>% plot(quantities = T)
```

```
# svg(
#   filename = 'results/figures_final/SUSORO_venn/SUSORO_venn_all.svg',
#   width = 7/2.54, height = 7/2.54,
#   pointsize = 10, family = 'sans',
#   bg = 'white'
# )
# euler(df_all) %>% plot(
#   quantities = T,
#   edges = list(lwd = 1.5),
#   fills = substance_cols %>% alpha(0.6)
# )
# dev.off()
# 
# svg(
#   filename = 'results/figures_final/SUSORO_venn/SUSORO_venn_late.svg',
#   width = 7/2.54, height = 7/2.54,
#   pointsize = 10, family = 'sans',
#   bg = 'white'
# )
# euler(df_late) %>% plot(
#   quantities = T,
#   edges = list(lwd = 1.5),
#   fills = substance_cols %>% alpha(0.6)
# )
# dev.off()


inner_all = 
  intersect(
    lapply(gene_sets_SU, function(x) {x$gene}) %>% do.call('c', .),
    c(
      lapply(gene_sets_SORO[1:6], function(x) {
        x$gene
      }) %>% do.call('c', .),
      results_SR$Sorafenib.e.96.C2 %>% filter(padj < 0.05) %>% pull(ensembl_gene_id)
    )
  ) %>% intersect(
    c(
      lapply(gene_sets_SORO[7:12], function(x) {
        x$gene
      }) %>% do.call('c', .),
      results_SR$Rotenone.e.96.C1 %>% filter(padj < 0.05) %>% pull(ensembl_gene_id)
    )
  )

inner_late = 
  intersect(
    lapply(gene_sets_SU[10:15], function(x) {
      x$gene
    }) %>% do.call('c', .),
    lapply(gene_sets_SORO[1:6], function(x) {
        x$gene
      }) %>% do.call('c', .)
  ) %>% 
  intersect(
    lapply(gene_sets_SORO[7:12], function(x) {
        x$gene
      }) %>% do.call('c', .)
  )

inner_late_top400 =
  list(
    sig_summ_24_p,
    sig_summ_rot,
    sig_summ_sora
  ) %>%
  lapply(function(x) {
    x %>% filter(gene %in% inner_late) %>% dplyr::select(gene, auci)
  }) %>% 
  do.call(rbind, .) %>% 
  group_by(gene) %>% 
  summarise(cumulative_auci = sum(auci)) %>% 
  arrange(desc(abs(cumulative_auci))) %>% 
  slice_head(n = 400) %>% 
  pull(gene)
```

For the top 400 genes from the intersection of all substances in the
late expoure scenario, an ORA is performed.

```
enrich_inner_late_top400 = enrich_all(
  inner_late_top400, enrichment_terms
) %>% filter(p.adjust < 0.05) %>% arrange(p.adjust)

enrich_inner_late_top400 %>% write_tsv('results/enrichments/SUSORO_late_intersection_top400_enrichment.tsv')
```

### Figures

#### Enrichments summary

ORA results are loaded and rearranged into lists for plotting later
on.

```
enr.SU4312.ORA = readRDS('data/enrichments_SU4312/enrichments_SU4312_ORA.rds')

enr.SR.ORA = readRDS('data/rotenone_sorafenib/enr_ORA_sora_rot.rds')
enr.SR.ORA.gam = readRDS('data/rotenone_sorafenib/enrichments_SR_ORA_gam.rds')

# all enrichments
enrichments_ORA_gam_all = list(
  SU4312.e.A.C1 = enr.SU4312.ORA$SU4312.e.A.C1,
  SU4312.e.D.C2 = enr.SU4312.ORA$SU4312.e.D.C2,
  SU4312.e.AD.int = enr.SU4312.ORA$SU4312.e.AD.int,
  SU4312.e.B.C1 = enr.SU4312.ORA$SU4312.e.B.C1,
  SU4312.e.E.C2 = enr.SU4312.ORA$SU4312.e.E.C2,
  SU4312.e.BE.int = enr.SU4312.ORA$SU4312.e.BE.int,
  SU4312.e.C.C1 = enr.SU4312.ORA$SU4312.e.C.C1,
  SU4312.e.F.C2 = enr.SU4312.ORA$SU4312.e.F.C2,
  SU4312.e.CF.int = enr.SU4312.ORA$SU4312.e.CF.int,
  SU4312.l.G.C2 = enr.SU4312.ORA$SU4312.l.G.C2,
  SU4312.l.I.C3 = enr.SU4312.ORA$SU4312.l.I.C3,
  SU4312.l.GI.int = enr.SU4312.ORA$SU4312.l.GI.int,
  SU4312.l.H.C2 = enr.SU4312.ORA$SU4312.l.H.C2,
  SU4312.l.J.C3 = enr.SU4312.ORA$SU4312.l.J.C3,
  SU4312.l.HJ.int = enr.SU4312.ORA$SU4312.l.HJ.int,
  Sorafenib.e.96.C2 = enr.SR.ORA.gam$Sora.e.96.C2,
  Sorafenib.l.K.C1 = enr.SR.ORA.gam$Sora.l.K.C1,
  Sorafenib.l.M.C2 = enr.SR.ORA.gam$Sora.l.M.C2,
  Sorafenib.l.KM.int = enr.SR.ORA.gam$Sora.l.KM.int,
  Sorafenib.l.L.C1 = enr.SR.ORA.gam$Sora.l.L.C1,
  Sorafenib.l.N.C2 = enr.SR.ORA.gam$Sora.l.N.C2,
  Sorafenib.l.LN.int = enr.SR.ORA.gam$Sora.l.LN.int,
  Rotenone.e.96.C1 = enr.SR.ORA.gam$Rote.e.96.C1,
  Rotenone.l.O.C1 = enr.SR.ORA.gam$Rote.l.O.C1,
  Rotenone.l.Q.C2 = enr.SR.ORA.gam$Rote.l.Q.C2,
  Rotenone.l.OQ.int = enr.SR.ORA.gam$Rote.l.OQ.int,
  Rotenone.l.P.C1 = enr.SR.ORA.gam$Rote.l.P.C1,
  Rotenone.l.R.C2 = enr.SR.ORA.gam$Rote.l.R.C2,
  Rotenone.l.PR.int = enr.SR.ORA.gam$Rote.l.PR.int
)
# only enrichments from intersections of DEGs from concentrations in the same time frame
enrichments_ORA_gam_int = list(
  SU4312.e.AD.int = enr.SU4312.ORA$SU4312.e.AD.int,
  SU4312.e.BE.int = enr.SU4312.ORA$SU4312.e.BE.int,
  SU4312.e.CF.int = enr.SU4312.ORA$SU4312.e.CF.int,
  SU4312.l.GI.int = enr.SU4312.ORA$SU4312.l.GI.int,
  SU4312.l.HJ.int = enr.SU4312.ORA$SU4312.l.HJ.int,
  Sorafenib.e.96.C2 = enr.SR.ORA.gam$Sora.e.96.C2,
  Sorafenib.l.KM.int = enr.SR.ORA.gam$Sora.l.KM.int,
  Sorafenib.l.LN.int = enr.SR.ORA.gam$Sora.l.LN.int,
  Rotenone.e.96.C1 = enr.SR.ORA.gam$Rote.e.96.C1,
  Rotenone.l.OQ.int = enr.SR.ORA.gam$Rote.l.OQ.int,
  Rotenone.l.PR.int = enr.SR.ORA.gam$Rote.l.PR.int
)
```

A summary of numbers of DEGs and numbers of significantly
over-represented terms is calculated.

```
df_summary = list(
  lapply(names(enr.SU4312.ORA), function(n) {
    data.frame(
      name = n,
      size_enr = enr.SU4312.ORA[[n]] %>% filter(p.adjust < 0.05) %>% nrow(),
      size_geneset = gene_sets_SU[[n]]$gene %>% n_distinct(),
      type = 'SU4312 ora'
    )
  }) %>% do.call(rbind, .),
  lapply(names(enr.SR.ORA.gam), function(n) {
    data.frame(
      name = n,
      size_enr = enr.SR.ORA.gam[[n]] %>% filter(p.adjust < 0.05) %>% nrow(),
      size_geneset = gene_sets_SORO[[n]]$gene %>% n_distinct(),
      type = 'SR ora gam'
    )
  }) %>% do.call(rbind, .),
  lapply(names(enr.SR.ORA[1:2]), function(n) {
    if (is.data.frame(enr.SR.ORA[[n]])) {
      data.frame(
        name = n,
        size_enr = enr.SR.ORA[[n]] %>% filter(p.adjust < 0.05) %>% nrow(),
        size_geneset = results_SR[[n]] %>% filter(padj < 0.05) %>% nrow(),
        type = 'SR ora'
      )
    } else {
      data.frame(
        name = n,
        size_enr = 0,
        size_geneset = results_SR[[n]] %>% filter(padj < 0.05) %>% nrow(),
        type = 'SR ora'
      )
    }
  }) %>% do.call('rbind', .)
) %>% do.call('rbind', .)

df_summary
```

```
# df_summary %>% write_tsv('results/SUSORO_degs_detailed.tsv')

df_summary_2 = data.frame(
  SU_all = lapply(gene_sets_SU, function(x) {x$gene}) %>% do.call('c', .) %>% n_distinct(),
  SU_early = lapply(gene_sets_SU[1:9], function(x) {x$gene}) %>% do.call('c', .) %>% n_distinct(),
  SU_late = lapply(gene_sets_SU[10:15], function(x) {x$gene}) %>% do.call('c', .) %>% n_distinct(),
  SO_all = lapply(gene_sets_SORO[1:6], function(x) {x$gene}) %>% do.call('c', .) %>% n_distinct() +
    results_SR$Sorafenib.e.96.C2 %>% filter(padj < 0.05) %>% nrow(),
  SO_early = results_SR$Sorafenib.e.96.C2 %>% filter(padj < 0.05) %>% nrow(),
  SO_late = lapply(gene_sets_SORO[1:6], function(x) {x$gene}) %>% do.call('c', .) %>% n_distinct(),
  RO_all = lapply(gene_sets_SORO[7:12], function(x) {x$gene}) %>% do.call('c', .) %>% n_distinct() +
    results_SR$Rotenone.e.96.C1 %>% filter(padj < 0.05) %>% nrow(),
  RO_early = results_SR$Rotenone.e.96.C1 %>% filter(padj < 0.05) %>% nrow(),
  RO_late = lapply(gene_sets_SORO[7:12], function(x) {x$gene}) %>% do.call('c', .) %>% n_distinct()
) %>% t() %>% as.data.frame() %>% set_names('DEGs') %>% as_tibble(rownames = 'condition')

df_summary_2 %>% write_tsv('results/SUSORO_degs.tsv')
```

`term_overview_combined()` takes a list of ORAs, from each
ORA it takes the top n terms (by lowest adj. p, top n determined by
`top_n` argument), combines them and aggregates a data frame
with adjusted p-values and general trend of regulation
(median(AUCI95%) across all genes in a gene set) for all
terms and conditions.

```
term_overview_data = list()
term_overview_data[['SUSORO.mean.ORA.gam']] = term_overview_combined(enrichments_ORA_gam_int, enrichment_terms, top_n = 7)

term_overview_data[['SUSORO.all.ORA.gam']] = term_overview_combined(enrichments_ORA_gam_all, enrichment_terms, top_n = 5)

term_overview_data[['SU4312.ORA']] = term_overview_combined(enr.SU4312.ORA, enrichment_terms, top_n = 7)

saveRDS(term_overview_data, 'data/term_overview_data.rds')
```

The data is plotted in a bubble plot, where the size of a bubble
corresponds to the adjusted p-value and fill to the
median(AUCI95%).

```
term_overview_data = readRDS('data/term_overview_data.rds')

# SUSORO intersections of concentrations only
p = plot_term_overview_pval(term_overview_data$SUSORO.mean.ORA.gam, enrichments_ORA_gam_int, lim = 0.25, legend_title = 'median difference\nto control', text_w = Inf) +
  geom_vline(xintercept = c(3.5, 6.5)) +
  geom_vline(xintercept = c(1.5, 4.5, 9.5), colour = 'grey70') +
  # ggtitle('SU4312, Sorafenib, Rotenone mean ORA') +
  scale_size(limits = c(0, NA), range = c(0, 5), breaks = -log(c(1 ,0.05, 1e-2, 1e-5, 1e-20))) +
  theme(
    legend.position = 'none',
    axis.text = element_text(family = 'Arial', size = 8, lineheight = 4),
    axis.text.y = element_text(lineheight = 0.5),
    axis.text.x = element_blank()
  )
ggsave(
  'bubble_plot_wo_legend.svg',
  p,
  'svg',
  'results/figures_final/SUSORO_venn_bubble_blood_vasc/',
  width = 21.5,
  height = 15,
  unit = 'cm',
  bg = 'white'
)

p = plot_term_overview_pval(term_overview_data$SUSORO.mean.ORA.gam, enrichments_ORA_gam_int, lim = 0.25, legend_title = 'median difference\nto control', text_w = 40) +
  geom_vline(xintercept = c(3.5, 6.5)) +
  geom_vline(xintercept = c(1.5, 4.5, 9.5), colour = 'grey70') +
  # ggtitle('SU4312, Sorafenib, Rotenone mean ORA') +
  scale_size(limits = c(0, NA), range = c(0, 6), breaks = -log(c(1 ,0.05, 1e-2, 1e-5, 1e-20))) +
  theme(
    legend.text = element_text(size = 10, family = 'Arial'),
    legend.direction = 'vertical'
  )
p = get_legend(p)
ggsave(
  'bubble_plot_legend.svg',
  p,
  'svg',
  'results/figures_final/SUSORO_venn_bubble_blood_vasc/',
  width = 10,
  height = 10,
  unit = 'cm',
  bg = 'white'
)


# SUSORO all conditions + intersections of concentrations
plot_term_overview_pval(term_overview_data$SUSORO.all.ORA.gam, enrichments_ORA_gam_all, lim = 0.25, legend_title = 'median difference\nto control') +
  geom_vline(xintercept = c(7.5, 14.5)) +
  geom_vline(xintercept = c(1.5, 8.5, 23.5), colour = 'grey70') +
  ggtitle('SU4312, Sorafenib, Rotenone mean ORA')
```

```
# SU all conditions + intersections of concentrations
plot_term_overview_pval(term_overview_data$SU4312.ORA, enr.SU4312.ORA, lim = 0.5, legend_title = 'median difference\nto control') +
  geom_vline(xintercept = c(12.5)) +
  geom_vline(xintercept = c(3.5, 6.5, 9.5, 15.5, 18.5), , colour = 'grey70') +
  ggtitle('SU4312 all ORA')
```

```
# # SO all conditions + intersections of concentrations
# plot_term_overview_pval(term_overview_data$Sorafenib.ORA, enr.SR.ORA.gam, lim = 0.5, legend_title = 'median difference\nto control') +
#   geom_vline(xintercept = c(1.5)) +
#   geom_vline(xintercept = c(4.5, 7.5), colour = 'grey70') +
#   ggtitle('Sorafenib all ORA')
# 
# # RO all conditions + intersections of concentrations
# plot_term_overview_pval(term_overview_data$Rotenone.ORA, enr.SR.ORA.gam, lim = 0.5, legend_title = 'median difference\nto control') +
#   geom_vline(xintercept = c(1.5)) +
#   geom_vline(xintercept = c(4.5, 7.5), colour = 'grey70') +
#   ggtitle('Rotenone all ORA')
```

#### Gene set overviews

`gene_set_overview_spline()` summarises expression
patterns for gene sets. It takes a gene set as input and displays the
temporal expression patterns in a heatmap for the selected substances
and exposure scenarios.

```
p = gene_set_overview_spline(
  genes = enrichment_terms$ZFIN$term2gene %>%
    filter(term == 'ZFA:0001079') %>%
    pull(gene) %>% unique(),
  ci_diff_list = list(ci_diff_0_gam, ci_diff_24_gam, ci_diff_rot, ci_diff_sora),
  term = 'ZFA:0001079',
  take_mean = T,
  cluster = T,
  limits = c(-1.5, 1.5)
) +
  theme(
    axis.text.y = element_text(family = 'Arial', size = 7),
    axis.text.x = element_text(family = 'Arial', size = 8),
    legend.text = element_text(family = 'Arial', size = 8),
    legend.position = 'none',
    axis.title = element_text(family = 'Arial', size = 8),
    strip.text = element_text(family = 'Arial', size = 8),
    panel.spacing = unit(1.75, 'mm')
  )
p
```

```
ggsave('results/figures_final/SUSORO_venn_bubble_blood_vasc/blood_vasc.tiff', plot = p, 'tiff', width = 9.5, height = 13.75, units = 'cm', bg = 'white')
ggsave('results/figures_final/SUSORO_venn_bubble_blood_vasc/blood_vasc.svg', plot = p, 'svg', width = 9.5, height = 13.75, units = 'cm', bg = 'white')
```

```
gene_set_overview_spline(
  genes = inner_late,
  ci_diff_list = list(ci_diff_24_gam, ci_diff_rot, ci_diff_sora),
  take_mean = T,
  cluster = T,
  limits = c(-1.5, 1.5)
) +
  theme(
    axis.text = element_text(family = 'Arial', size = 10),
    legend.text = element_text(family = 'Arial', size = 10),
    axis.title = element_text(family = 'Arial', size = 11),
    strip.text = element_text(family = 'Arial', size = 11),
    axis.ticks = element_line(linewidth = 0.5),
    axis.ticks.length = unit(0.6, 'mm')
  )
```

For the top 250 SU4312 DEGS from the early exposure and the top 250
DEGs from the late exposure, a heatmap is created. The heatmap is
clustered into 19 clusters, for them ORAs are performed. This figure is
shown in the main text.

```
ORA_clusters = function(clust, matrix, k) {
  clusters = make_cluster_heatmap(clust, matrix, k, return_clusters = T)
  clusters_list = lapply(unique(clusters$cluster_name), function(c) {
    clusters$gene[clusters$cluster_name == c]
  }) %>% set_names(unique(clusters$cluster_name))
  enr_clusters = lapply(clusters_list, function(genes) {
    if (length(genes) >= 5) {
      enrich_all(genes, enrichment_terms) %>%
        add_column(clustersize = length(genes)) %>%
        filter(p.adjust < 0.1)
    } else {
      data.frame(ensembl_gene_id = genes) %>%
        left_join(data_0$genes[, c('ensembl_gene_id',
                                   'external_gene_name',
                                   'description',
                                   'name_1006')], by = 'ensembl_gene_id')
    }
  })
  return(enr_clusters)
}

write_excel_ORA_clusters = function(ORA, filename) {
  wb = createWorkbook()
  for (n in names(ORA)) {
    add_data(wb, n, ORA[[n]])
  }
  saveWorkbook(wb, filename, overwrite = T)
}

g_cumu_auci_early = list(gene_sets_SU[1:9]) %>% unlist(recursive = F) %>% 
  do.call(rbind, .) %>% 
  group_by(ensembl_gene_id) %>% 
  summarise(sum_AUCI_h = sum(ci_per_h)) %>% 
  arrange(desc(abs(sum_AUCI_h))) %>% 
  pull(ensembl_gene_id)

g_cumu_auci_late = list(gene_sets_SU[10:15]) %>% unlist(recursive = F) %>% 
  do.call(rbind, .) %>% 
  group_by(ensembl_gene_id) %>% 
  summarise(sum_AUCI_h = sum(ci_per_h)) %>% 
  arrange(desc(abs(sum_AUCI_h))) %>% 
  pull(ensembl_gene_id)

g_500 = c(g_cumu_auci_early[1:250], g_cumu_auci_late[1:250]) %>% unique()

clust = gene_set_overview_spline(g_500,
                         list(ci_diff_0_gam, ci_diff_24_gam),
                         return_hclust = T)

matrix = gene_set_overview_spline(g_500,
                         list(ci_diff_0_gam, ci_diff_24_gam),
                         return_matrix = T)

# plot using ggplot
p = gene_set_overview_spline(
  genes = g_500,
  ci_diff_list = list(ci_diff_0_gam, ci_diff_24_gam),
  take_mean = F,
  cluster = T,
  limits = c(-4.5, 4.5)
) +
  theme(
    axis.text = element_text(family = 'Arial', size = 10),
    legend.position = 'none',
    axis.text.y = element_blank(),
    axis.title = element_text(family = 'Arial', size = 11),
    strip.text = element_text(family = 'Arial', size = 11),
    axis.ticks = element_line(linewidth = 0.5),
    axis.ticks.length = unit(0.6, 'mm'),
    axis.ticks.y = element_blank(),
    strip.background = element_blank()
  )

ggsave('results/figures_final/fig3_SU_top500_heatmap/SU_top500_heatmap_ggplot.tiff', plot = p, 'tiff', width = 13, height = 19, units = 'cm', bg = 'white', dpi = 600)

# plot using gplots
tiff(filename = 'results/figures_final/fig3_SU_top500_heatmap/SU_top500_heatmap.tiff',
     width = 26, height = 40, units = 'cm',
     compression = 'lzw',
     res = 600, bg = 'white')
make_cluster_heatmap(clust, matrix, 19, colsep = c(0, 94, 94*2, (94*2+70), (94*2+70*2)))
dev.off()

ora = ORA_clusters(clust, matrix, 19)
write_excel_ORA_clusters(ora, '../results/enrichments/SU4312_top500_cluster_enrichments.xlsx')

g_500_DEG_table = gene_set_overview_spline(
  genes = g_500,
  ci_diff_list = list(ci_diff_0_gam, ci_diff_24_gam),
  take_mean = F,
  return_df = T
)
g_500_DEG_table = g_500_DEG_table %>% 
  add_column(time_frame = cut(.$t, breaks = c(0, 24, 72, 100), labels = c('0-24hpf', '24-72hpf', '72-96hpf'))) %>% 
  group_by(ensembl_gene_id, contrast, time_frame) %>% 
  summarise(
    auci_per_h = sum(diff)/n()
  ) %>% 
  ungroup() %>% 
  add_column(condition = paste(.$time_frame, .$contrast, sep = '.')) %>% 
  mutate(
    time_frame = NULL,
    contrast = NULL
  ) %>% 
  pivot_wider(
    names_from = condition,
    values_from = auci_per_h,
    names_prefix = 'auci_per_h.'
  ) %>% 
  left_join(data_0$genes[, c('ensembl_gene_id', 'external_gene_name')], by = 'ensembl_gene_id') %>% 
  relocate(external_gene_name, .after = 1)
  
write_tsv(g_500_DEG_table, 'results/top_tables/SU_top500_DEGs.tsv')
```

For the top 400 DEGs DE in all three substances in the late
exposures, a heatmnap is made.

```
ci_list = list(
  ci_diff_24_gam %>% filter(contrast == 'C3'),
  ci_diff_sora %>% filter(contrast == 'C2'),
  ci_diff_rot %>% filter(contrast == 'C2')
)

# clust = gene_set_overview_spline(inner_late_top400,
#                          ci_list,
#                          return_hclust = T)
# 
# matrix = gene_set_overview_spline(inner_late_top400,
#                          ci_list,
#                          return_matrix = T)
# 
# n_clusters = 20
# make_cluster_heatmap(clust, matrix, n_clusters)

p = gene_set_overview_spline(
  genes = inner_late_top400,
  ci_diff_list = ci_list,
  take_mean = F,
  cluster = T,
  limits = c(-2.5, 2.5)
) +
  theme(
    axis.text = element_text(family = 'Arial', size = 10),
    # legend.position = 'none',
    axis.text.y = element_text(family = 'Arial', size = 4),
    axis.title = element_text(family = 'Arial', size = 11),
    strip.text = element_text(family = 'Arial', size = 11),
    axis.ticks = element_line(linewidth = 0.5),
    axis.ticks.length = unit(0.6, 'mm'),
    axis.ticks.y = element_blank(),
    strip.background = element_blank()
  )
p
```

```
ggsave('results/figures_final/SUSORO_intersection_heatmap/SUSORO_intersection_heatmap_ggplot.tiff', plot = p, 'tiff', width = 15, height = 40, units = 'cm', bg = 'white', dpi = 600)

inner_late_DEG_table = gene_set_overview_spline(
  genes = inner_late,
  ci_diff_list = list(ci_diff_24_gam,
                      ci_diff_sora,
                      ci_diff_rot),
  take_mean = F,
  return_df = T
)
inner_late_DEG_table = inner_late_DEG_table %>% 
  add_column(time_frame = cut(.$t, breaks = c(0, 72, 100), labels = c('24-72hpf', '72-96hpf'))) %>% 
  group_by(substance, ensembl_gene_id, contrast, time_frame) %>% 
  summarise(
    auci_per_h = sum(diff)/n()
  ) %>% 
  ungroup() %>% 
  add_column(condition = paste(.$substance, .$contrast, sep = '.')) %>% 
  mutate(
    substance = NULL,
    contrast = NULL
  ) %>% 
  pivot_wider(
    names_from = condition,
    values_from = auci_per_h,
    names_prefix = 'auci_per_h.'
  )
  
write_tsv(inner_late_DEG_table, 'results/top_tables/SUSORO_intersection_DEGs.tsv')
```

Legends are generated and saved to be inserted into figures
later.

```
# generating a legend
limits = c(-4.5, 4.5)
p = data.frame(x = 1:2, y = 1:2, z = limits) %>%
  ggplot(aes(x = x, y = y, colour = limits)) +
  geom_point() +
  scale_colour_gradient2(
      low = 'dodgerblue3',
      mid = 'white',
      high = 'firebrick3',
      name = 'logFC',
      midpoint = 0,
      limits = limits,
      oob = scales::squish
    ) +
  theme(legend.position = 'bottom')
leg = ggpubr::get_legend(p) %>% ggpubr::as_ggplot()

svg(
  filename = 'results/figures_final/fig3_SU_top500_heatmap/heatmap_legend_4.5.svg',
  width = 3, height = 2
)
leg
dev.off()
```

```
## png 
##   2
```

```
limits = c(-2.5, 2.5)
p = data.frame(x = 1:2, y = 1:2, z = limits) %>%
  ggplot(aes(x = x, y = y, colour = limits)) +
  geom_point() +
  scale_colour_gradient2(
      low = 'dodgerblue3',
      mid = 'white',
      high = 'firebrick3',
      name = 'logFC',
      midpoint = 0,
      limits = limits,
      oob = scales::squish
    ) +
  theme(legend.position = 'bottom')
leg = ggpubr::get_legend(p) %>% ggpubr::as_ggplot()

svg(
  filename = 'results/figures_final/SUSORO_intersection_heatmap/heatmap_legend_2.5.svg',
  width = 3, height = 2
)
leg
dev.off()
```

```
## png 
##   2
```

```
# legend for blood vasc figure
limits = c(-1.5, 1.5)
p = data.frame(x = 1:2, y = 1:2, z = limits) %>%
  ggplot(aes(x = x, y = y, colour = limits)) +
  geom_point() +
  scale_colour_gradient2(
      low = 'dodgerblue3',
      mid = 'white',
      high = 'firebrick3',
      name = 'logFC',
      midpoint = 0,
      limits = limits,
      oob = scales::squish
    ) +
  theme(legend.position = 'bottom')
leg = ggpubr::get_legend(p) %>% ggpubr::as_ggplot()

svg(
  filename = 'results/figures_final/SUSORO_venn_bubble_blood_vasc/heatmap_legend_1.5.svg',
  width = 3, height = 2
)
leg
dev.off()
```

```
## png 
##   2
```

## Information

This document was created with the following command:

```
rmarkdown::render(
    input = 'SUSORO_analysis.Rmd',
    output_format = 'html_document',
    output_file = 'supplementary_methods.html'
)
```

**R version 4.3.0 (2023-04-21)**

**Platform:** x86\_64-pc-linux-gnu (64-bit)

**attached base packages:**

- stats4
- splines
- stats
- graphics
- grDevices
- utils
- datasets
- methods
- base

**other attached packages:**

- org.Dr.eg.db(v.3.18.0)
- AnnotationDbi(v.1.64.1)
- clusterProfiler(v.4.10.1)
- MASS(v.7.3-60.0.1)
- eulerr(v.7.0.2)
- cowplot(v.1.1.3)
- heatmap3(v.1.1.9)
- gplots(v.3.1.3.1)
- PoiClaClu(v.1.0.2.1)
- ggh4x(v.0.2.8)
- openxlsx(v.4.2.5.2)
- DESeq2(v.1.42.1)
- SummarizedExperiment(v.1.32.0)
- Biobase(v.2.62.0)
- MatrixGenerics(v.1.14.0)
- matrixStats(v.1.3.0)
- GenomicRanges(v.1.54.1)
- GenomeInfoDb(v.1.38.8)
- IRanges(v.2.36.0)
- S4Vectors(v.0.40.2)
- BiocGenerics(v.0.48.1)
- lubridate(v.1.9.3)
- forcats(v.1.0.0)
- stringr(v.1.5.1)
- dplyr(v.1.1.4)
- purrr(v.1.0.2)
- readr(v.2.1.5)
- tidyr(v.1.3.1)
- tibble(v.3.2.1)
- ggplot2(v.3.5.0)
- tidyverse(v.2.0.0)
- reticulate(v.1.35.0)
- RCy3(v.2.22.1)
- igraph(v.2.0.3)
- data.table(v.1.15.4)
- enrichplot(v.1.22.0)
- mgcv(v.1.9-1)
- nlme(v.3.1-164)
- limma(v.3.58.1)

**loaded via a namespace (and not attached):**

- fs(v.1.6.3)
- bitops(v.1.0-7)
- sf(v.1.0-16)
- HDO.db(v.0.99.1)
- httr(v.1.4.7)
- RColorBrewer(v.1.1-3)
- repr(v.1.1.7)
- tools(v.4.3.0)
- backports(v.1.4.1)
- utf8(v.1.2.4)
- R6(v.2.5.1)
- lazyeval(v.0.2.2)
- withr(v.3.0.0)
- gridExtra(v.2.3)
- cli(v.3.6.2)
- textshaping(v.0.3.7)
- bcrypt(v.1.1)
- scatterpie(v.0.2.2)
- labeling(v.0.4.3)
- sass(v.0.4.9)
- proxy(v.0.4-27)
- pbapply(v.1.7-2)
- askpass(v.1.2.0)
- systemfonts(v.1.0.6)
- yulab.utils(v.0.1.4)
- pbdZMQ(v.0.3-11)
- gson(v.0.1.0)
- DOSE(v.3.28.2)
- svglite(v.2.1.3)
- rstudioapi(v.0.16.0)
- RSQLite(v.2.3.6)
- generics(v.0.1.3)
- gridGraphics(v.0.5-1)
- gtools(v.3.9.5)
- vroom(v.1.6.5)
- car(v.3.1-2)
- zip(v.2.3.1)
- GO.db(v.3.18.0)
- Matrix(v.1.6-5)
- fansi(v.1.0.6)
- abind(v.1.4-5)
- lifecycle(v.1.0.4)
- yaml(v.2.3.8)
- edgeR(v.4.0.16)
- carData(v.3.0-5)
- qvalue(v.2.34.0)
- SparseArray(v.1.2.4)
- grid(v.4.3.0)
- blob(v.1.2.4)
- crayon(v.1.5.2)
- lattice(v.0.22-6)
- KEGGREST(v.1.42.0)
- pillar(v.1.9.0)
- knitr(v.1.46)
- fgsea(v.1.28.0)
- codetools(v.0.2-20)
- fastmatch(v.1.1-4)
- glue(v.1.7.0)
- ggvenn(v.0.1.10)
- ggfun(v.0.1.4)
- vctrs(v.0.6.5)
- png(v.0.1-8)
- treeio(v.1.26.0)
- gtable(v.0.3.4)
- cachem(v.1.0.8)
- xfun(v.0.43)
- ggpattern(v.1.0.1)
- S4Arrays(v.1.2.1)
- tidygraph(v.1.3.1)
- units(v.0.8-5)
- statmod(v.1.5.0)
- ggtree(v.3.10.1)
- bit64(v.4.0.5)
- bslib(v.0.7.0)
- KernSmooth(v.2.23-22)
- colorspace(v.2.1-0)
- DBI(v.1.2.2)
- tidyselect(v.1.2.1)
- bit(v.4.0.5)
- compiler(v.4.3.0)
- graph(v.1.80.0)
- DelayedArray(v.0.28.0)
- plotly(v.4.10.4)
- shadowtext(v.0.1.3)
- scales(v.1.3.0)
- caTools(v.1.18.2)
- classInt(v.0.4-10)
- digest(v.0.6.35)
- rmarkdown(v.2.26)
- XVector(v.0.42.0)
- htmltools(v.0.5.8.1)
- pkgconfig(v.2.0.3)
- base64enc(v.0.1-3)
- highr(v.0.10)
- fastmap(v.1.1.1)
- rlang(v.1.1.3)
- htmlwidgets(v.1.6.4)
- farver(v.2.1.1)
- jquerylib(v.0.1.4)
- jsonlite(v.1.8.8)
- BiocParallel(v.1.36.0)
- GOSemSim(v.2.28.1)
- RCurl(v.1.98-1.14)
- magrittr(v.2.0.3)
- GenomeInfoDbData(v.1.2.11)
- ggplotify(v.0.1.2)
- patchwork(v.1.2.0)
- IRkernel(v.1.3.2)
- munsell(v.0.5.1)
- Rcpp(v.1.0.12)
- RPostgreSQL(v.0.7-6)
- ape(v.5.8)
- viridis(v.0.6.5)
- stringi(v.1.8.3)
- ggraph(v.2.2.1)
- RJSONIO(v.1.3-1.9)
- zlibbioc(v.1.48.2)
- plyr(v.1.8.9)
- parallel(v.4.3.0)
- ggrepel(v.0.9.5)
- Biostrings(v.2.70.3)
- graphlayouts(v.1.1.1)
- IRdisplay(v.1.1)
- pander(v.0.6.5)
- hms(v.1.1.3)
- polylabelr(v.0.2.0)
- locfit(v.1.5-9.9)
- ggpubr(v.0.6.0)
- uuid(v.1.2-0)
- fastcluster(v.1.2.6)
- ggsignif(v.0.6.4)
- base64url(v.1.4)
- reshape2(v.1.4.4)
- XML(v.3.99-0.16.1)
- evaluate(v.0.23)
- tzdb(v.0.4.0)
- tweenr(v.2.0.3)
- openssl(v.2.1.1)
- polyclip(v.1.10-6)
- ggforce(v.0.4.2)
- broom(v.1.0.5)
- e1071(v.1.7-14)
- tidytree(v.0.4.6)
- rstatix(v.0.7.2)
- viridisLite(v.0.4.2)
- class(v.7.3-22)
- ragg(v.1.3.0)
- aplot(v.0.2.2)
- memoise(v.2.0.1)
- gridpattern(v.1.1.1)
- toxprofileR2(v.0.3.0)
- timechange(v.0.3.0)
