# Supplementary figures and images for: Dynamics in zebrafish development define transcriptomic specificity after angiogenesis inhibitor exposure
Source: Arch Toxicol. 2025 Jan 9;99(4):1561–78. doi: 10.1007/s00204-024-03944-7 (PMC11968557; doi:10.1007/s00204-024-03944-7)

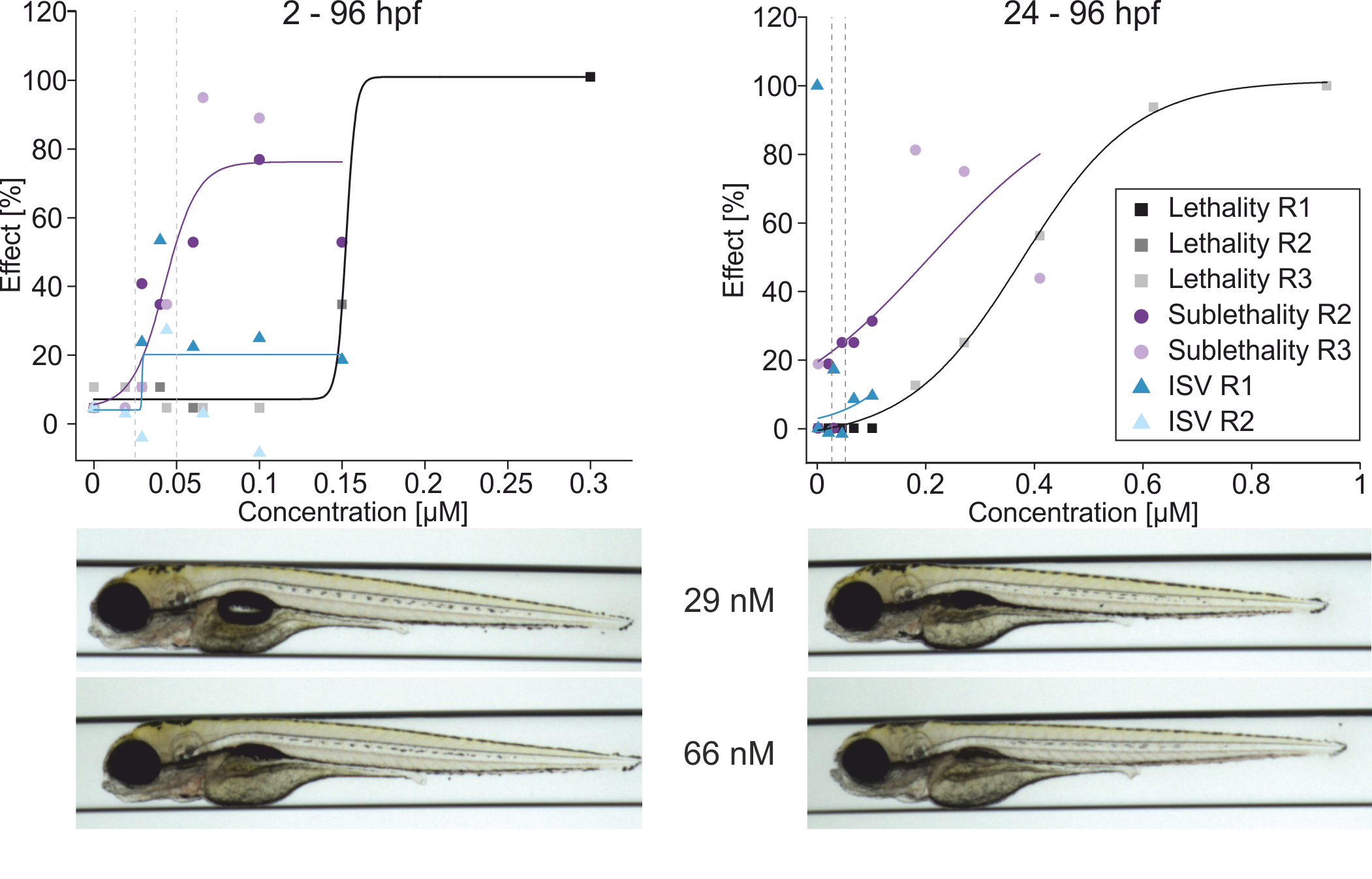

Supplement: Supplementary file 2 — Supplementary file2 (PNG 1632 KB) [file 204_2024_3944_MOESM2_ESM.png]

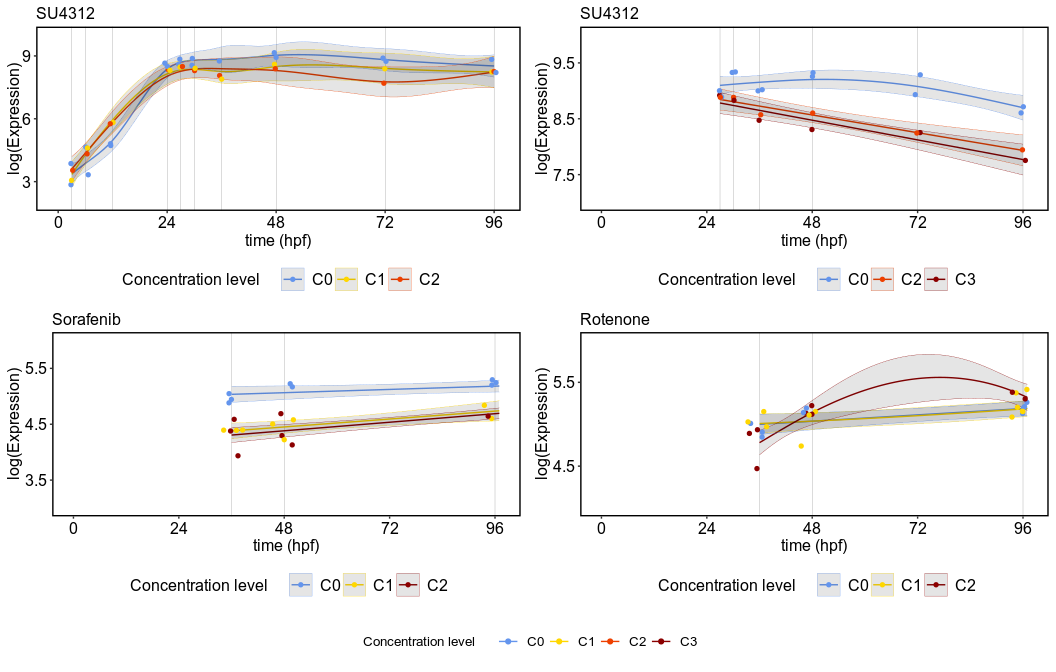

Supplement: Supplementary file 3 — Supplementary file3 (PNG 92 KB) [file 204_2024_3944_MOESM3_ESM.png]

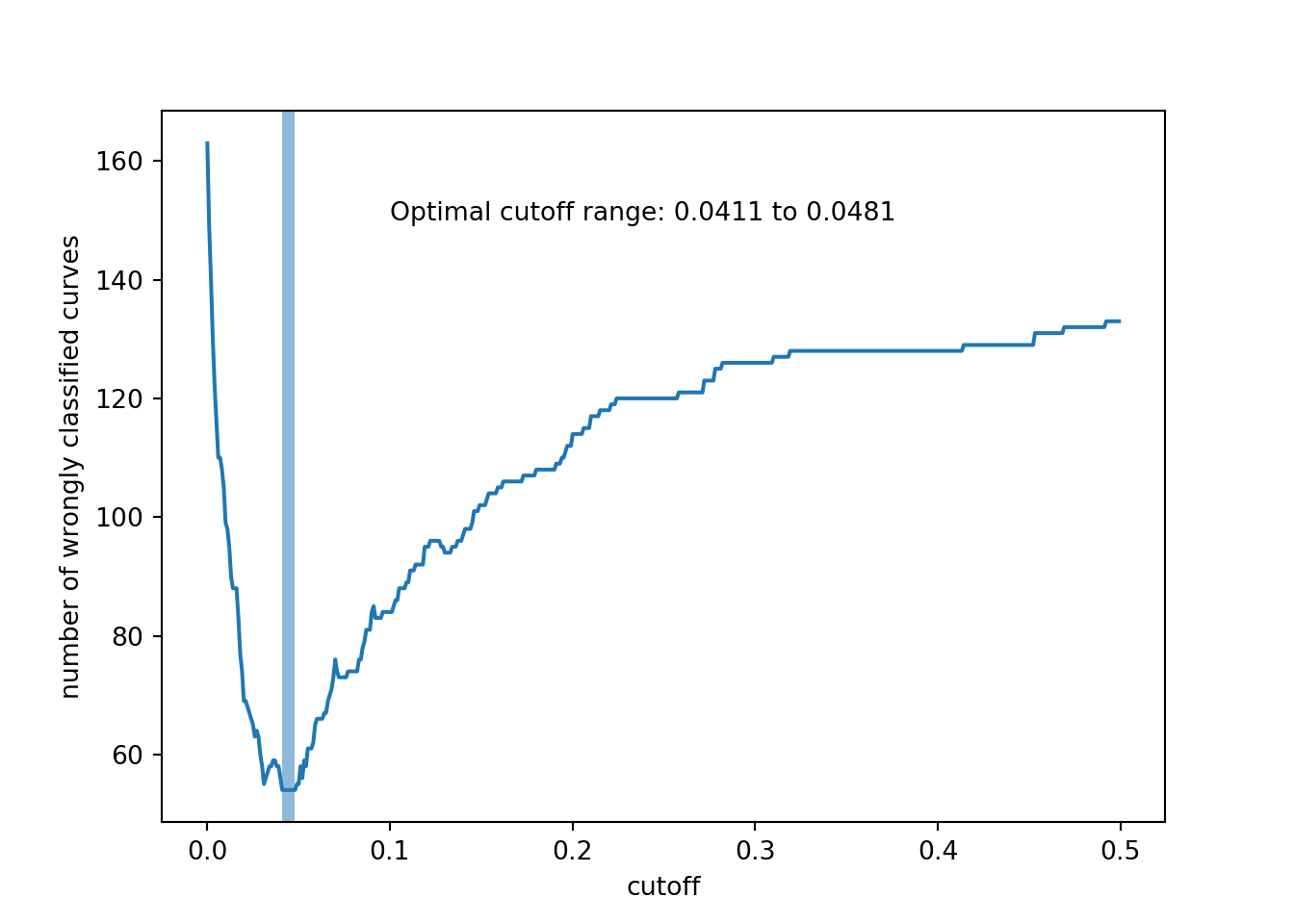

Supplement: Supplementary file 4 — Supplementary file4 (PNG 58 KB) [file 204_2024_3944_MOESM4_ESM.png]

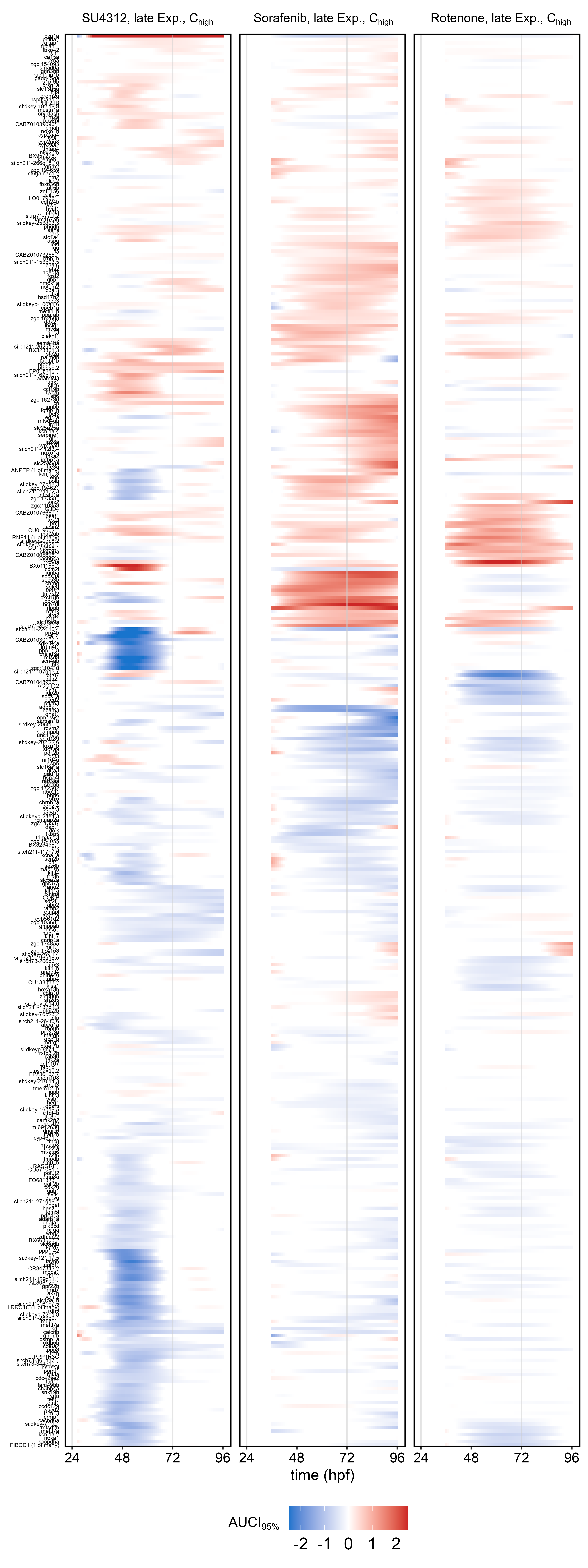

Supplement: Supplementary file 12 — Supplementary file12 (PNG 1472 KB) [file 204_2024_3944_MOESM12_ESM.png]
